# Supplementary material for: MicroBundleCompute: Automated segmentation, tracking, and analysis of subdomain deformation in cardiac microbundles
Source: PLoS One. 2024 Mar 26;19(3):e0298863. doi: 10.1371/journal.pone.0298863 (PMC10965069; doi:10.1371/journal.pone.0298863)
Supplement: S1 Appendix — Generating synthetic examples of both “Type 1” and “Type 2” as well as validating “MicroBundleCompute” against these synthetic data in their original form and with added Perlin noise are described and discussed in more detail. Table S1_1. Summary of validating tracked displacement against synthetic data of “Type 1” without any added Perlin noise. Tracked mean absolute displacement is compared against a known ground truth. Table S1_2. Summary of validating Ecc strain against synthetic data of “Type 1” without any added Perlin noise. Computed Ecc strain from tracked displacement data is compared against a known ground truth. Table S1_3. Tabulated summary of validating Ecc strain outputs of “MicroBundleCompute” against all synthetic data of “Type 1” without any added Perlin noise. Computed Ecc strain from tracked displacement data is compared against a known ground truth. Table S1_4. Summary of validating tracked displacement against synthetic data of “Type 1” based on homogeneous activation with added Perlin noise. Tracked mean absolute displacement is compared against a known ground truth and the “best” and “worst” results are reported. Table S1_5. Summary of validating Ecc strain from tracked displacement data against synthetic data of “Type 1” based on homogeneous activation with added Perlin noise. Computed Ecc strain from tracked displacement data is compared against a known ground truth and the “best,” “average,” and “worst” findings are reported. Fig S1_1. Typical microbundle dimensions of “Type 1.” Schematic representation of the “Type 1” microbundle mesh implemented in our Finite Element simulations. Fig S1_2. Convergence study of the grid size used to warp the microbundle textures to generate synthetic data. Both x and y positions of the mesh cell centers are compared to a ground truth with respect to grid size. Fig S1_3. A tabulated summary of the implemented conditions to obtain 400 noisy synthetic examples. Main differences between the generated synthetic [file pone.0298863.s001.pdf]

MicroBundleCompute: Automated segmentation, tracking, and analysis of subdomain deformation in cardiac microbundles
S1 Appendix: Validation pipeline

In this Supplementary Document, we elaborate on the "Synthetic data generation" Section and provide further details on our approach to validate the output of our cardiac microbundle tracking software. In the "Methods" Section, we describe the steps to generate Finite Element Analysis (FEA)-informed synthetic movies of beating microbundles with a known ground truth. We also provide details on our approach to collect ground truth data via manual tracking performed on real examples. Then in the "Results" Section, we present the results obtained by comparing the tracked output generated by the software and the ground truth data, and comment on the accuracy and robustness of our computational framework.

Methods

Synthetic dataset

In this Section, we describe additional details of our approach to creating realistic brightfield movies of beating cardiac microbundles, as depicted in Fig 2 of the main document. We note that our synthetic dataset is primarily derived from "Type 1" examples, since "Type 1" cardiac microbundle data is a more common approach in the literature.

For the "Type 1" synthetic data, we first start by identifying frames from which we can extract tissue textures. We systematically choose frames that represent valley and peak positions of the beating microbundle (i.e., the most relaxed or most contracted states) in order to sample multiple distinct textures (Fig 2a). We then manually trace the microbundle element in a valley frame. From the obtained mask, we extract the coordinates of the mask contour and crop out the background of the movie frame to obtain an image that contains just the tissue texture (Fig 2b).

For the Finite Element (FE) simulations, we generate a microbundle model based on the contour coordinates of a mask extracted from a single representative valley frame using Gmsh 4.10.5 [1] (Fig 2c & Fig S1_1). By connecting the points representing the mask contour, we obtain a 2D tissue surface, which we then extrude to a representative thickness for our real "Type 1" data. To simulate pillars, we follow the design and dimensions detailed in [2], which corresponds to a commonly implemented platform design. The generated mesh consists of 205, 524 tetrahedral elements.

In Fig 2d, we briefly summarize the main components of the FE model as implemented in FEniCS 2019.1.0 [3,4]. Specifically, we model the cardiac microbundle as a nearly-incompressible hyperelastic material, following recent work in the literature [5–7]. We describe the incompressible hyperelastic material with the strain energy density function Ψ written as:

$$\Psi = \Psi(\mathbf{F}) - p(J-1) \tag{1}$$

where $\mathbf{F}$ is the deformation gradient tensor, p is a Lagrange multiplier to impose the incompressibility constraint, and $J = det(\mathbf{F})$ is the volume ratio and should be equal to unity for full incompressibility [8]. The scalar p acts as a reaction hydrostatic pressure resisting any volume change and can only be determined from the equilibrium equations and the boundary conditions.

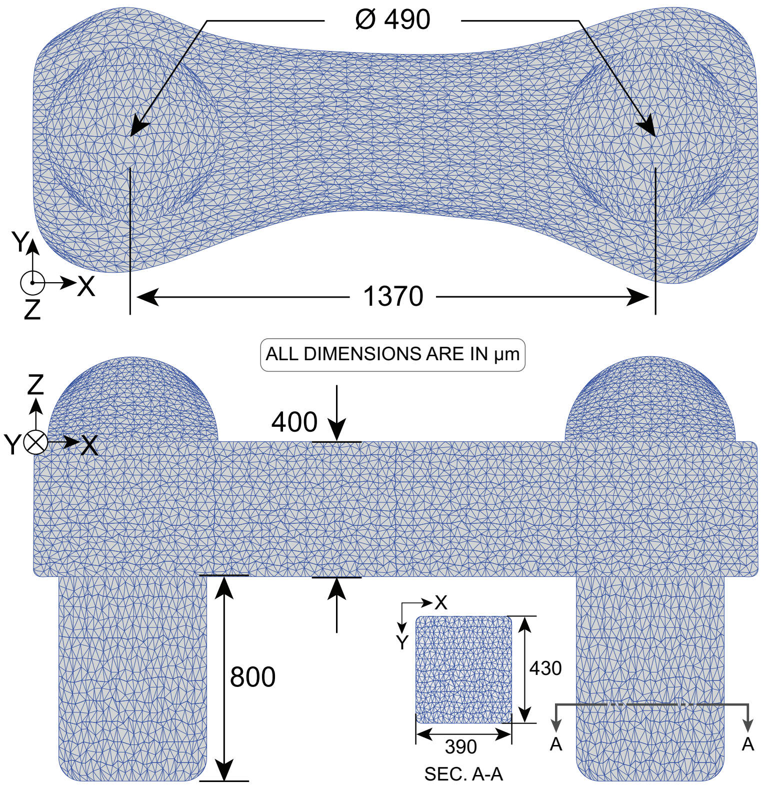

Fig S1_1. Schematic representation of the "Type 1" microbundle mesh implemented in our Finite Element simulations.

To relax the incompressibility constraint, we adopt the standard volumetric-isochoric decomposition of the deformation gradient tensor to obtain a more robust mathematical formulation of the constitutive model equations and avoid numerical difficulties such as mesh locking that arise when finite element methods are implemented in the analysis of incompressible materials [5, 9]. This representation can be derived following the multiplicative decomposition of the deformation gradient tensor into purely isochoric $\mathbf{F_{iso}}$ and purely volumetric $\mathbf{F_{vol}}$ components where $\mathbf{F} = \mathbf{F_{iso}}\mathbf{F_{vol}}$. As such we can write $\mathbf{F_{iso}} = J^{-1/3}\mathbf{I}$ and $\mathbf{F_{vol}} = J^{1/3}\mathbf{I}$.

The two components of the deformation gradient tensor contribute in an additive manner to the strain energy density function, where we can write:

$$\Psi(\mathbf{F}) = \Psi_{iso}(\mathbf{F_{iso}}) + \Psi_{vol}(\mathbf{F_{vol}}) \tag{2}$$

The isochoric component of the strain energy density function can be defined as any hyperelastic model. Here, we consider a Neo-Hookean material model:

$$\Psi_{iso} = \frac{1}{2}\mu\big[tr\,(\mathbf{C_{iso}}) - 3\big] \tag{3}$$

where μ is the shear modulus and $\mathbf{C_{iso}} = \mathbf{F_{iso}^T}\mathbf{F_{iso}} = J^{-2/3}\mathbf{F^T}\mathbf{F}$ with $\mathbf{C}$ being the right Cauchy-Green tensor. As for the volumetric strain energy, several forms are cited in the literature. In our simulation, we define $\Psi_{vol} = \frac{\kappa}{2}ln(J)^2$ where κ is the bulk modulus of the material, which is orders of magnitude higher than the nearly-incompressible material's shear modulus. Finally, for nearly-incompressible formulation, incompressibility is implemented by the constraint relating the Lagrange multiplier p and Ψ_{vol} where $p = -d\Psi_{vol}(J)/dJ$.

In the numerical setting, we employ a mixed formulation finite element method with Taylor-Hood tetrahedral elements [10], where the displacement field is represented by a continuous piecewise quadratic Lagrange shape function ($P2$) while the pressure field is represented by a continuous piecewise linear Lagrange shape function ($P1$).

Finally, to model the active properties of the cardiac tissue and its ability to actively contract and generate force without external loads, we implement the active strain approach [11]. In brief, this approach is based on a multiplicative decomposition of the deformation gradient tensor $\mathbf{F}$ into an active component $\mathbf{F_a}$ and an elastic component $\mathbf{F_e}$ [5, 7]. For a transversely isotropic activation, $\mathbf{F_a}$ is defined as:

$$\mathbf{F_a} = (1-\gamma)\,\mathbf{f_0} \otimes \mathbf{f_0} + \frac{1}{\sqrt{1-\gamma}}\,(\mathbf{I} - \mathbf{f_0} \otimes \mathbf{f_0}) \tag{4}$$

where γ is the activation in the fiber direction and $\mathbf{f_0}$ is the fiber axis. The elastic part $\mathbf{F_e}$ is defined as $\mathbf{F_e} = \mathbf{F}\mathbf{F_a^{-1}}$. Applying this to the isochoric deformation gradient, we get $(\mathbf{F_{iso}})_\mathbf{e} = \mathbf{F_{iso}}\mathbf{F_a^{-1}}$. Finally, the strain energy density will be a function of $\mathbf{F_e}$ only, and following our previous decomposition, the Neo-Hookean material model can be written as:

$$\Psi_{iso} = \frac{1}{2}\mu\,[tr\,(\mathbf{C_{iso}})_\mathbf{e} - 3] \tag{5}$$

where $(\mathbf{C_{iso}})_\mathbf{e} = (\mathbf{F^T_{iso}})_\mathbf{e}(\mathbf{F_{iso}})_\mathbf{e}$.

We define a uniform periodic time series activation ranging between 0 and a maximum activation value (see Fig 2d - lower right inset) along the fiber axis as:

$$\gamma = \begin{cases} -A\cos(\omega t) & \gamma \geq 0.008 \\ 0 & \gamma < 0.008 \end{cases} \qquad \text{with} \quad 0 \leq \omega \leq 2\pi \tag{6}$$

To introduce slight variability to the periodic function, we add correlated Brownian (red) noise [12]. The lower left and middle insets in Fig 2d provide a general depiction of the defined fiber direction. In our simulations, we linearly vary the fiber angle α defined with the global X-axis (horizontal) from a lower value of 9.33° to a maximum of 15.33° in two different tissue dimensions: 1) with respect to tissue depth (global Z-axis, into the page), where the fiber angle is 9.33° on the top surface of the tissue ($Z = 0$) and becomes 15.33° at the tissue's bottom ($Z = -400\mu$m) and 2) with respect to tissue length (global X-axis, horizontal), where the fiber angle is 9.33° on the left side of the tissue ($X = 0$) and becomes 15.33° at the tissue's right end.

We also simulate spatially heterogeneous activation by including a circular passive region in the middle of the tissue where the activation is zero and increases gradually with distance from the periphery of the circle to reach the maximum defined activation value at the current step. We implement this by defining an inclusion ratio as:

$$r = \begin{cases} 1 & d < r_{min} \\ \frac{r^2_{min}}{\beta r^2_{min} + (1-\beta)d} & d \geq r_{min} \end{cases} \qquad \text{with} \quad d = S_\alpha(\|P - p_1\|, ..., \|P - p_n\|) \tag{7}$$

where r_{min} is the radius of the circular inclusion, $\alpha = -80$, $\beta = 0.9$, P is the center of the inclusion, $p_1, ..., p_n$ are the tissue spatial mesh coordinates, $\|\quad\|$ denotes the Euclidean distance, and S_α is the smooth maximum approximation function given by:

$$S_\alpha(x_1, ...x_n) = \frac{\sum_{i=1}^{n} x_i e^{\alpha x_i}}{\sum_{i=1}^{n} e^{\alpha x_i}} \qquad \text{where} \quad S_\alpha \to min \quad \text{as} \quad \alpha \to -\infty \tag{8}$$

and calculate the heterogeneous activation as $\gamma_h = \gamma(1 - r)$.

As for the pillars, we define a passive compressible Neo-Hookean constitutive model as:

$$\Psi = \frac{1}{2}\,\mu\left[\mathbf{F}:\mathbf{F} - 3 - 2\ln(\det\mathbf{F})\right] + \frac{1}{2}\,\lambda\left[\frac{1}{2}[(\det\mathbf{F})^2 - 1] - \ln(\det\mathbf{F})\right] \tag{9}$$

where $\mathbf{F}$ is the deformation gradient, and μ and λ are the Lamé parameters equivalent to Young's modulus E and Poisson's ratio ν as $E = \mu\,(3\lambda + 2\mu)/(\lambda + \mu)$ and $\nu = \lambda/(2(\lambda + \mu))$. We specify a value of 0.47 for the Poisson's ratio, and a value of 1.6 MPa for the Young's modulus.

From these Finite Element simulations, we extract the X, Y, and Z, positions of the mesh cell centers at the top surface of the microbundle for each step. We then estimate a projective transformation based on the initial and deformed positions of the mesh cell centers and warp the image texture accordingly using the "warp" transform function in the scikit-image 0.19.3 Python library [13]. To deform the synthetic texture with a heterogeneous transformation, we divide the domain into smaller slightly overlapping subdomains and deform each one according to a projective transformation calculated specifically for the subdomain. We perform a convergence study to decide on an appropriate number of subdomains and choose a grid size that results in the lowest error between the deformation gradient mapping obtained from FE simulations $\mathbf{F_{FEA}}$ and its equivalent that is obtained from approximating a projective transformation $\mathbf{F_{projective}}$ (Fig S1_2). In our final implementation, we divide each synthetic texture domain into 16×16 subdomains.

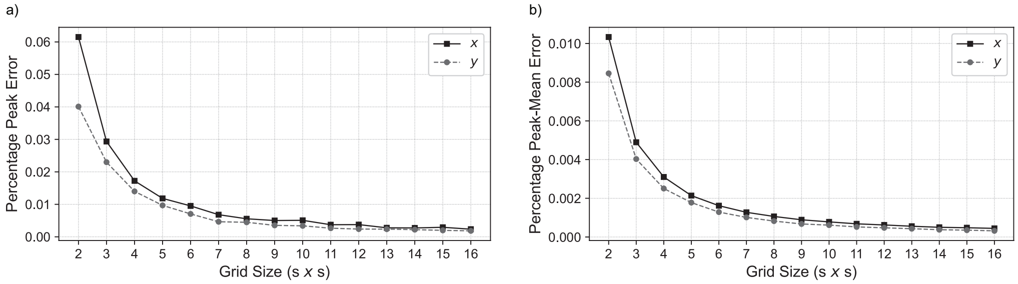

Fig S1_2. Convergence of the percentage error of the x and y positions of the mesh cell centers in comparison to a ground truth with respect to grid size: (a) The peak (maximum) percentage error in all the $s \times s$ grids for each respective grid size; (b) The peak (maximum) of the mean percentage error in each grid of the total $s \times s$ grids.

Overall, our synthetic dataset consists of 60 generated movies of beating microbundle textures. We extract 90×90 pixel regions for the tissue textures from 3 different frames per movie (2 valleys and 1 peak) and employ for this purpose, 5 experimental movies from "Type 1." Thus, we obtain $3 \times 5 = 15$ different base texture images. We then deform these extracted textures with FE results from 4 different FE simulations: 1) homogeneous activation across the whole microbundle domain with the fiber direction varying linearly in the X direction, 2) homogeneous activation across the whole microbundle domain with the fiber direction varying linearly in the Z direction, 3) heterogeneous activation where the active microbundle domain has a passive inclusion in the middle with the fiber direction varying linearly in the X direction, and finally, 4) heterogeneous activation where the active microbundle domain has a passive inclusion in the middle with the fiber direction varying linearly in the Z direction. We briefly note that all the files required to reproduce our complete synthetic dataset of "Type 1" along with the dataset files are made available on Github (https://github.com/HibaKob/SyntheticMicroBundle).

As a final step, we add spatially correlated Perlin noise [14], to both make the

dataset more realistic and to create more challenging examples to test the robustness of our computational framework. We add Perlin noise at varying levels of magnitude defined as a ratio of the maximum image intensity, and with a varying number of octaves as shown in Fig S1_4. From these examples, we see that the addition of noise to the synthetic images leads to accrued drift that is similar to what we observe when tracking displacements in real data.

For code testing and validation, we select a subset of the entire synthetic dataset consisting of 16 original synthetic movies, with half of this testing data corresponding to homogeneous activation and the other half to heterogeneous activation, as depicted in Fig S1_3. For each of these 16 examples, we add Perlin noise of 5 different magnitude ratios and 5 different octaves (Fig S1_3). In total, we run our code on 400 noisy test examples with known ground truth. We provide the results in the "Results" Section.

	Homogeneous activation							
**Name**	ST_1	ST_5	ST_14	ST_18	ST_29	ST_33	ST_42	ST_46

	Heterogeneous activation							
**Name**	ST_3	ST_7	ST_16	ST_20	ST_51	ST_52	ST_55	ST_56

	Perlin noise parameters				
**Magnitude ratio**	4%	8%	12%	16%	20%
**Octaves**	8	24	40	56	72

Total: 16 (test movies) **x 5** (magnitude ratios) **x 5** (octaves) **= 400** (test examples)

Fig S1_3. A tabulated summary of the synthetic data along with the added Perlin noise parameters implemented for code validation. Here, "ST" refers to "synthetic texture."

Finally, we include a single synthetic example with known ground truth based on "Type 2" data. The main steps to generate this example are similar to the ones followed when generating "Type 1" synthetic data; that is: 1) extracting the microbundle texture, 2) running a FE simulation mimicking the microbundle behavior, and finally, 3) warping the extracted texture with displacement results obtained from the FE simulations. These FE simulations are based on a tissue-specific model that has a detailed representation of fibers and cardiomyocytes as described in Jilberto et al. [15], which makes the kinematic behavior similar to that of real tissues. We provide this synthetic video with the supplementary material as "S1 Movie" and the performance of our framework on this example in the "Results" Section.

a)

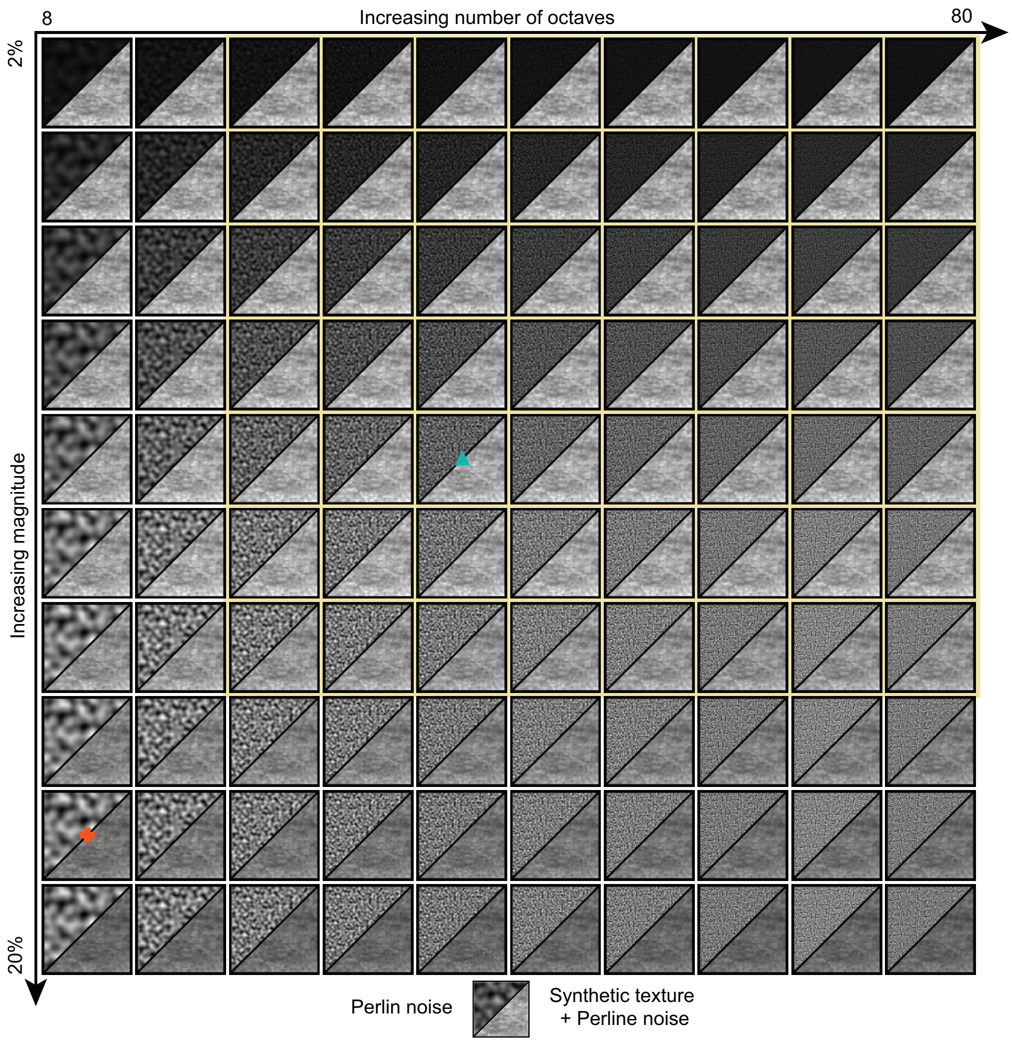
8
Increasing number of octaves
80
2%
Increasing magnitude
20%
Perlin noise
Synthetic texture
+ Perline noise

b)

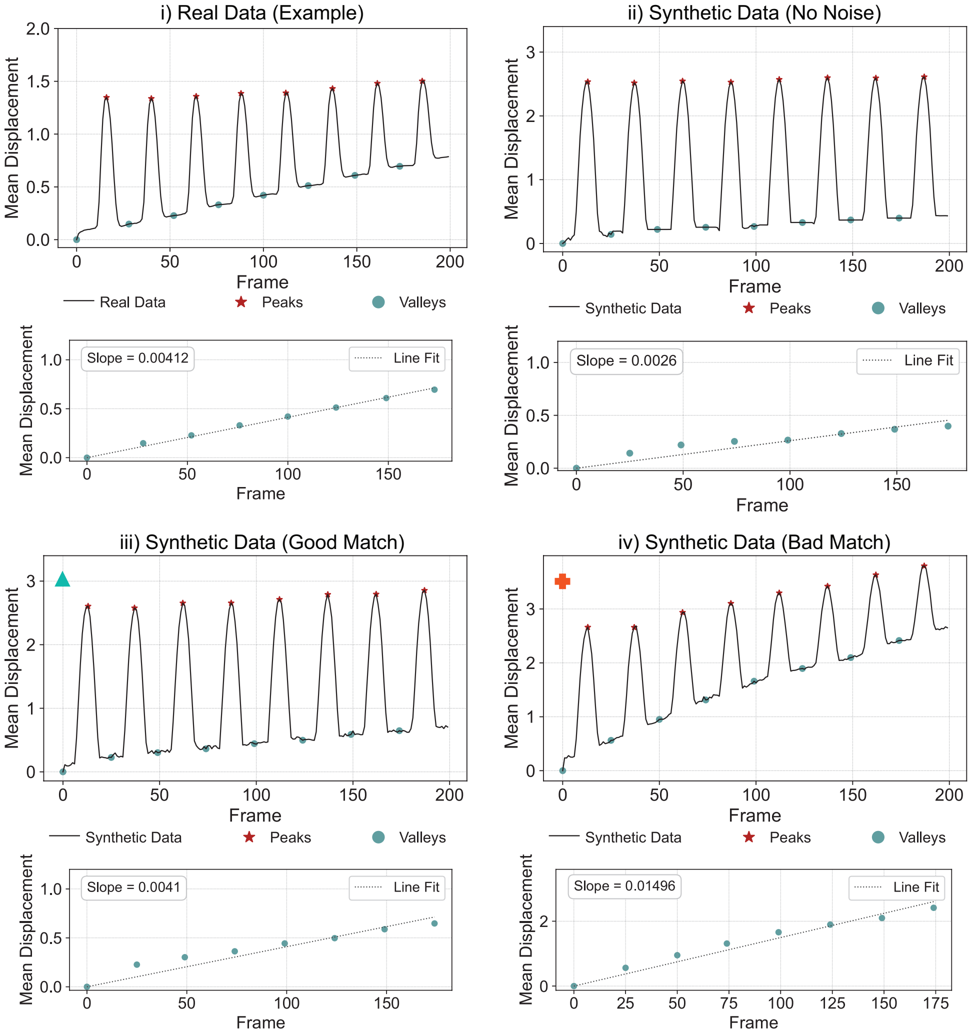

i) Real Data (Example)
ii) Synthetic Data (No Noise)
iii) Synthetic Data (Good Match)
iv) Synthetic Data (Bad Match)
Mean Displacement
Frame
Real Data
Synthetic Data
Peaks
Valleys
Line Fit
Slope = 0.00412
Slope = 0.0026
Slope = 0.0041
Slope = 0.01496

c)

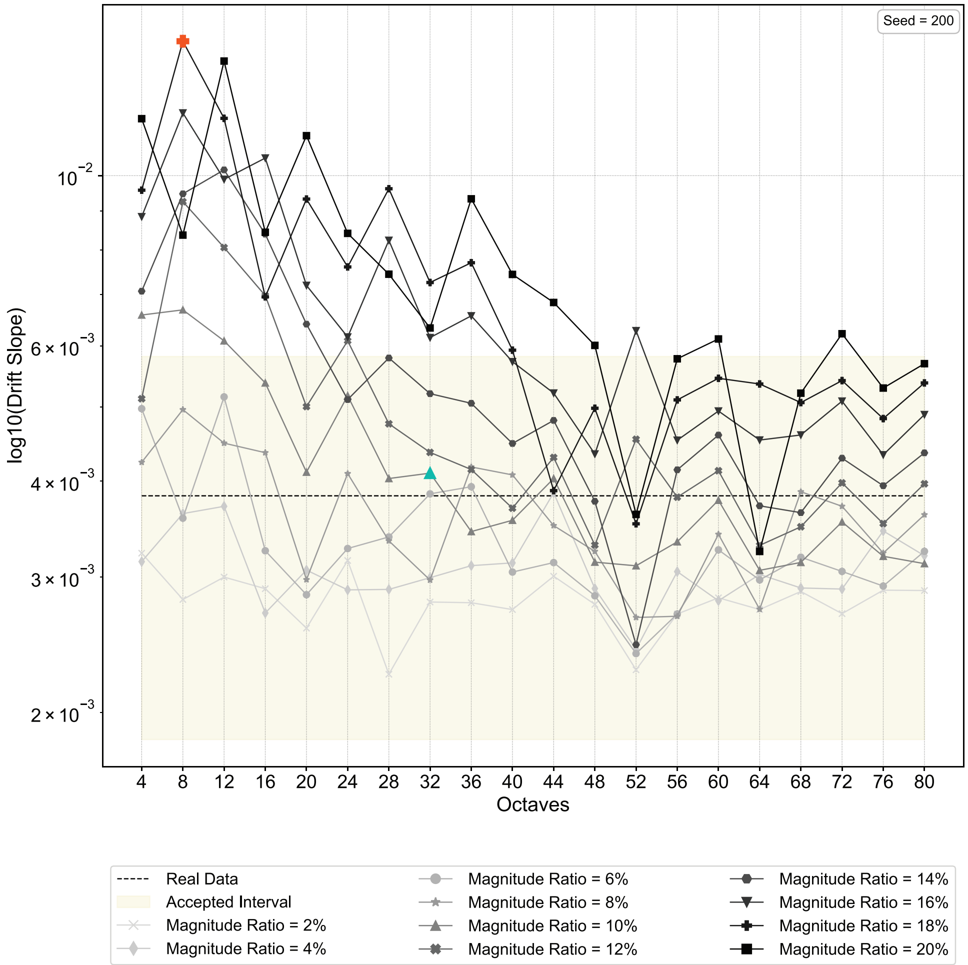

Fig S1_4. To make our synthetic dataset more challenging and more representative of real experimental data, we add Perlin noise to the synthetic microbundle textures: (a) the upper triangle shows an illustration of Perlin noise (a function of magnitude and octave), and the lower triangle shows the Perlin noise superimposed on the synthetic microbundle texture; (b) time series plots of (i) an example of tracked real data showing the drift in valleys along with the value of the slope of the linear fit of the valleys, (ii) an example of synthetic data without the addition of Perlin noise where it is clear that the drift in valleys is negligible, (iii) noisy synthetic data generated with 32 octaves and 10% magnitude ratio for which the slope of the drift is a good match to real data, (iv) an example of excessive distortion to the time series caused by the addition of Perlin noise generated with 18 octaves and 8% magnitude ratio; (c) a quantitative comparison of the drift in valleys (measured by the base 10 logarithm of the slope of the drift) with respect to the octave and magnitude ratio. From this analysis, we observe that there are a range of Perlin noise parameters (highlighted with a yellow background) that lead to drift behavior that is consistent with the average observed in 5 real examples of "Type 1" data. From this investigation, we: 1) show that adding Perlin noise to our synthetic data leads to synthetic data that better recapitulates the challenges associated with the real experimental data, and 2) identify the characteristics of the Perlin noise that we will use in our validation dataset.

Manual tracking

Another approach to validate our computational pipeline is to track the displacements of manually selected points and compare the results to those obtained by our tracking software. Specifically, we track the positions of 30 points across a single beat. We perform the manual tracking on two different examples of "Type 2" data, where each example is tracked by 2 different users (Fig S1_27 "Case 1" & "Case 2"). We include the results of this manual tracking validation the "Results" in Section.

Results

Validation against synthetic data

Here, we provide the results of validating "MicroBundleCompute" against synthetic data of beating microbundles. For the generated data based on "Type 1" data without any added Perlin noise, we show the computed errors for both mean absolute displacement (MAD) (Figs S1_5 and S1_6) and subdomain-averaged E_{cc} strain (strain in the column-column or horizontal-horizontal direction) (Table S1_3 and Figs S1_7 – S1_22) obtained by comparing the tracking software output to the known ground truth. For the 16 original validation examples, we base our error analysis on 4 basic metrics: 1) percentage error at the peak MAD, 2) coefficient of determination (R^2) between tracked and ground truth MAD for each beat, 3) coefficient of determination (R^2) between tracked and ground truth subdomain-averaged E_{cc} for each beat per subdomain, and 4) the mean absolute error (MAE) in E_{cc} per subdomain per beat.

For synthetic examples that are based on homogeneous activation simulations, where the resulting microbundle contractions exceed a single pixel, the percentage errors at peak MAD fall between 2% and 7% for all examples (Table S1_1). Overall, as shown in Table S1_1, the R^2 values indicate good agreement between the tracked and ground truth mean absolute displacement profiles, with 0.992 being the lowest R^2 value at the maximum error. For *sub*-pixel displacements, however, the peak MAD error range rises to 5% – 15% and the lowest $R2$ value drops to 0.939 (Table S1_1). As such, we consider tracked *sub*-pixel displacements to be of lower fidelity and advise the user of "MicroBundleCompute" software to interpret results derived from *sub*-pixel displacements with caution.

For the E_{cc} strain outputs, the computed errors are subdomain dependent. Overall, consistent with the behavior observed for MAD errors, synthetic examples based on homogeneous activation simulations exhibit significantly lower strain errors, with a maximum MAE of 9.2% of the peak ground truth E_{cc} in the corresponding subdomain versus an extreme value of 98% for *sub*-pixel displacement simulations (Table S1_2). In Table S1_3, we present the complete summary of the MAE E_{cc} strain validation performed on all synthetic examples.

As anticipated, the introduction of Perlin noise to "Type 1" synthetic dataset results in higher tracking errors as shown in Figs S1_23 and S1_24 and summarized in Tables S1_4 and S1_5 for the homogeneous activation cases. Again, movies with *sub*-pixel displacements are adversely affected by noise addition to a point where the code fails to identify beats to track. Such cases are indicated by missing data points in Fig S1_24. Overall, the errors that arise from the addition of Perlin noise decrease as the number of octaves increases, and the magnitude ratio decreases. For homogeneously activated data, the maximum MAD remains less than 14.5% (Table S1_4) for all tested cases, even those marked as extremely noisy compared to realistic data, as shown in Fig S1_4. However, this is not the case with heterogeneously activated examples where the MAD errors become unreasonably high in most cases (on the order of $10^3 - 10^4$).

Table S1_1. Summary of the mean absolute displacement validation errors for synthetic data without any added Perlin noise.

			Mean absolute displacement	
		Synthetic data example	Minimum R²	Maximum peak %error
Homogeneous activation	Best finding	ST_42	0.998	2%
	Worst finding	ST_29	0.992	7%
Heterogeneous activation	Best finding	ST_16	0.996	5%
	Worst finding	ST_56	0.970	15%
	Worst finding	ST_20	0.939	13%

Table S1_2. Summary of the mean absolute E_{cc} strain validation errors for synthetic data without any added Perlin noise.

			Subdomain Ecc strain				
		Synthetic data example	Minimum R²	Maximum MAE	Subdomain	Maximum subdomain Ecc	%MAE/ GT Ecc
Homogeneous activation	Best finding	ST_14	0.994	0.0020	1	| 0.0827 |	2.43%
	Worst finding	ST_1	0.908	0.0073	0	| 0.0796 |	9.18%
Heterogeneous activation	Best finding	ST_52	0.903	0.00064	2	| 0.0062 |	10.23%
	Worst finding	ST_3	-12.863	0.0060	0	| 0.0061 |	97.44%

In general, for E_{cc}, the MAE is below 36% but higher than 15% of the peak ground truth E_{cc} for all synthetic cases corresponding to homogeneous activation with added Perlin noise, except for the extreme case where the errors exceed 100% as shown in Table S1_5.

For "Type 2" synthetic data, we plot the analytical displacements in both X (horizontal) and Y (vertical) against their tracked equivalents obtained by running our optical flow pipeline (Fig S1_25). The results reveal good agreement between the two with R^2 values of 0.998 and 0.984 for displacements in X and Y, respectively. In Fig S1_26, we compare subdomain-averaged E_{cc} strain obtained via our tracking software to the known ground truth for a single beat. Overall, R^2 values (Fig S1_26a, c & d) reveal good agreement between tracked and ground truth subdomain-averaged E_{cc} per subdomain, with R^2 values being greater than 0.9. Furthermore, the mean absolute error in E_{cc} per subdomain (Fig S1_26b) indicates that the maximum error which occurs in subdomain `A4` is less than 7.4% of the peak ground truth E_{cc} in the respective subdomain.

Validation against manual tracking

In Fig S1_27, we show the comparison between the displacements in the X (horizontal) and Y (vertical) directions obtained via manual tracking to those obtained by "MicroBundleCompute" at the highlighted marker points. In both cases, the R^2 values indicate a good correlation between manual tracking results and the results from our computational tracking pipeline. Notably, the difference in tracking results between users 1 and 2 not only provides a range of values for comparison to our pipeline, but also indicates the fallibility of the manual tracking and the need for automated tools that lead to reproducible results.

Table S1_3. Summary of the mean absolute E_{cc} strain validation errors for all synthetic data without any added Perlin noise. (Corresponds to Figs S1_7–S1_22.)

Synthetic data example	Maximum MAE	R2	Subdomain	Maximum subdomain Ecc	%MAE/ GT Ecc
ST_1	7.30E-03	0.9135	0	|7.96E-02|	9.18%
ST_3*	5.96E-03	-12.6354	0	|6.12E-03|	97.44%
ST_5	6.22E-03	0.9274	2	|8.25E-02|	7.53%
ST_7*	3.27E-03	-3.2843	2	|6.51E-03|	50.23%
ST_14	2.01E-03	0.9941	1	|8.27E-02|	2.43%
ST_16*	7.22E-04	0.7900	3	|6.02E-03|	11.99%
ST_18	2.87E-03	0.9911	2	|8.23E-02|	3.49%
ST_20*	2.72E-03	-1.9509	0	|5.95E-03|	45.70%
ST_29	2.92E-03	0.9908	1	|8.21E-02|	3.56%
ST_33	4.04E-03	0.9754	3	|7.83E-02|	5.15%
ST_42	3.06E-03	0.9887	0	|8.11E-02|	3.77%
ST_46	3.53E-03	0.9852	0	|8.11E-02|	4.35%
ST_51*	7.61E-04	0.8430	2	|6.46E-03|	11.78%
ST_52*	6.36E-04	0.9035	2	|6.22E-03|	10.23%
ST_55*	1.49E-03	0.3419	1	|7.17E-03|	20.73%
ST_56*	1.65E-03	0.1886	1	|7.12E-03|	23.20%

Examples marked by an asterisk "*" indicate heterogeneous activation functions. We note that the R^2 values reported here correspond to the subdomain and beat for which the highest MAE for E_{cc} is observed.

Table S1_4. Summary of the mean absolute displacement validation errors for synthetic data with added Perlin noise.

		Synthetic data example	Maximum peak %error
**Homogeneous activation**	Best finding	ST_42	7.5%
	Worst finding	ST_18	14.4%

Table S1_5. Summary of the mean absolute $\mathbf{E_{cc}}$ strain validation errors for synthetic data with added Perlin noise.

			**Subdomain Ecc strain**			**Perlin noise parameters**	
		Synthetic data example	Maximum MAE	Maximum subdomain Ecc	%MAE/ GT Ecc	Magnitude ratio	Octaves
**Homogeneous activation**	Best finding	ST_42	0.0124	| 0.0811 |	15.29%	20	24
	Worst finding	ST_18	0.0915	| 0.0823 |	111.18%	16	8
	Less extreme	ST_1	0.0420	| 0.0796 |	52.76%	12	24
	General		0.0286	| 0.0813 |	35.18%		

The "Worst finding" example corresponds to the largest obtained error whereas the "Less extreme" finding corresponds to the second largest error. For the "General" case, we consider the mean of the maximum MAE values for the remaining synthetic examples based on homogeneous activation excluding examples ST_1, ST_18, and ST_42, as well as the mean of the maximum absolute ground truth subdomain E_{cc}.

Final remarks

Here, we have presented our two approaches to software validation: 1) comparison against synthetic data with known ground truth, and 2) comparison against manually labeled data ("Methods" Section). From the results shown in the "Results" Section, we see that our computational pipeline produces fairly accurate displacement results and slightly less accurate Green-Lagrange strain results, with the peak errors for both outputs falling below 10% for noiseless data exhibiting microbundle contractions that exceed a single pixel. For these examples with relatively large deformation, our software proves to be robust even against significant added noise. However, for *sub*-pixel deformation examples, the results are less reliable, with added Perlin noise drastically affecting their accuracy. This is important context for highlighting the applicability and limitations of our pipeline.

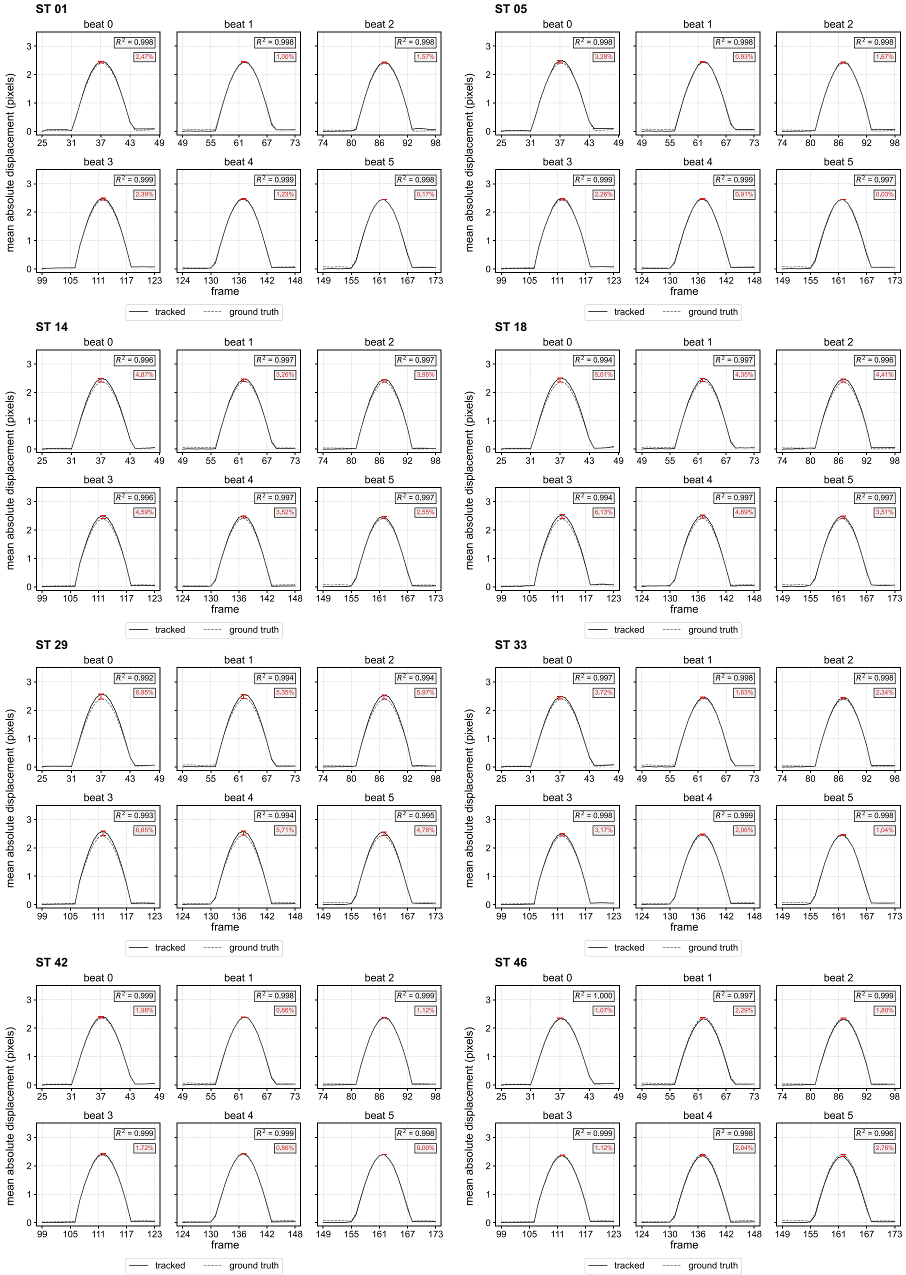

Fig S1_5. Error in mean absolute displacement for synthetic data of "Type 1" based on FE simulations with homogeneous activation.

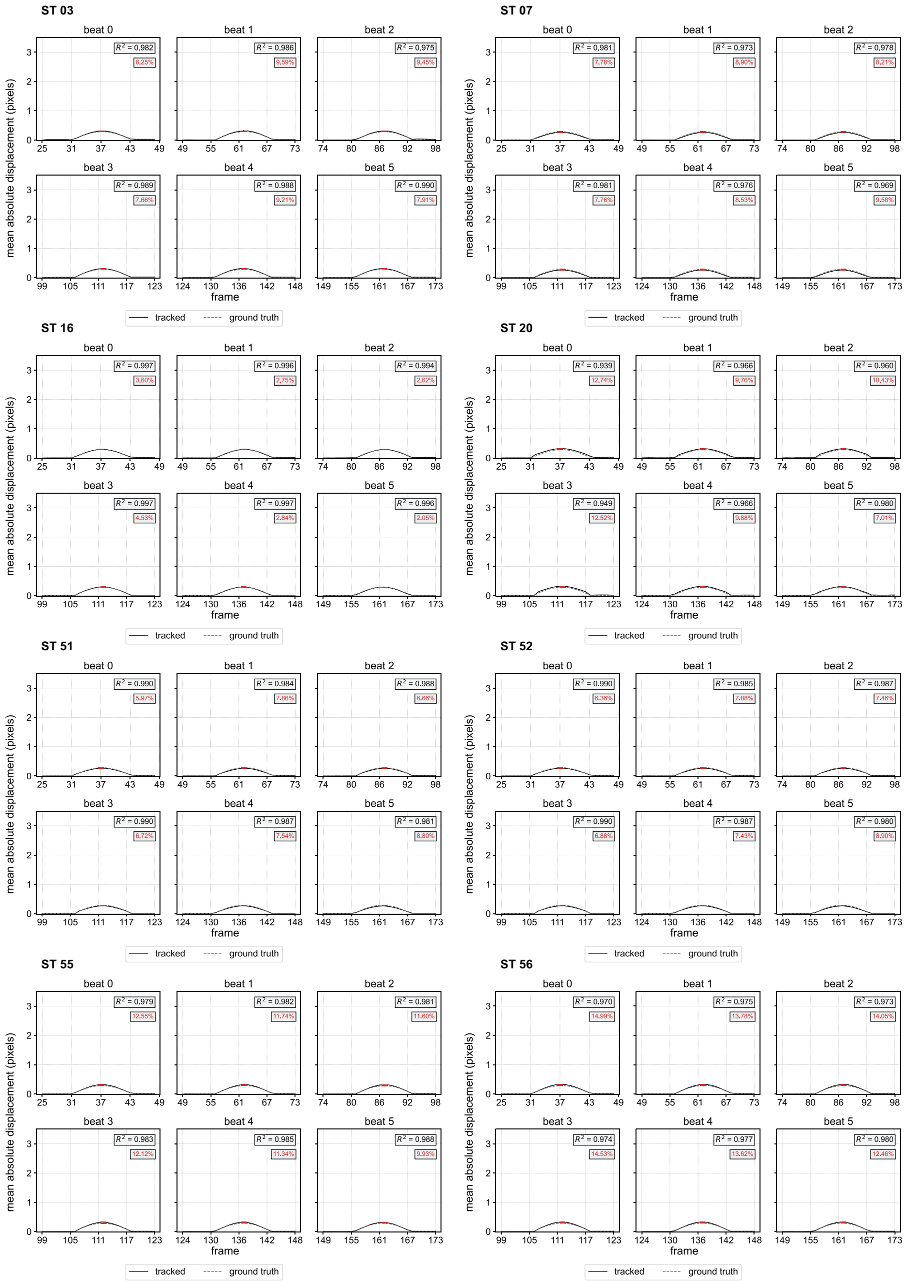

Fig S1_6. Error in mean absolute displacement for synthetic data of "Type 1" based on FE simulations with heterogeneous activation.

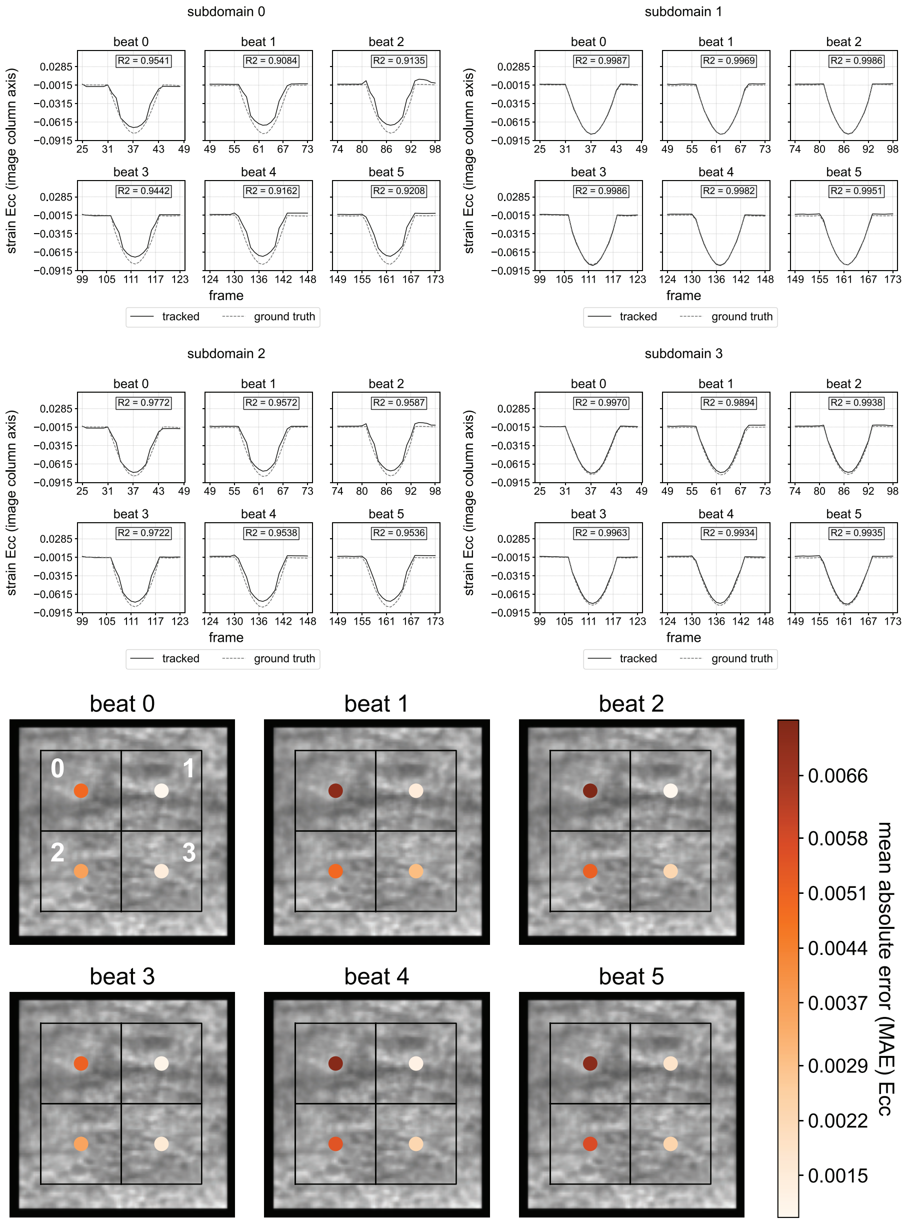

Fig S1_7. Error in E_{cc} strain for ``SyntheticTextures_1''. The numbers $0, 1, 2, 3$ shown on beat 0 MAE plot indicate the subdomain number.

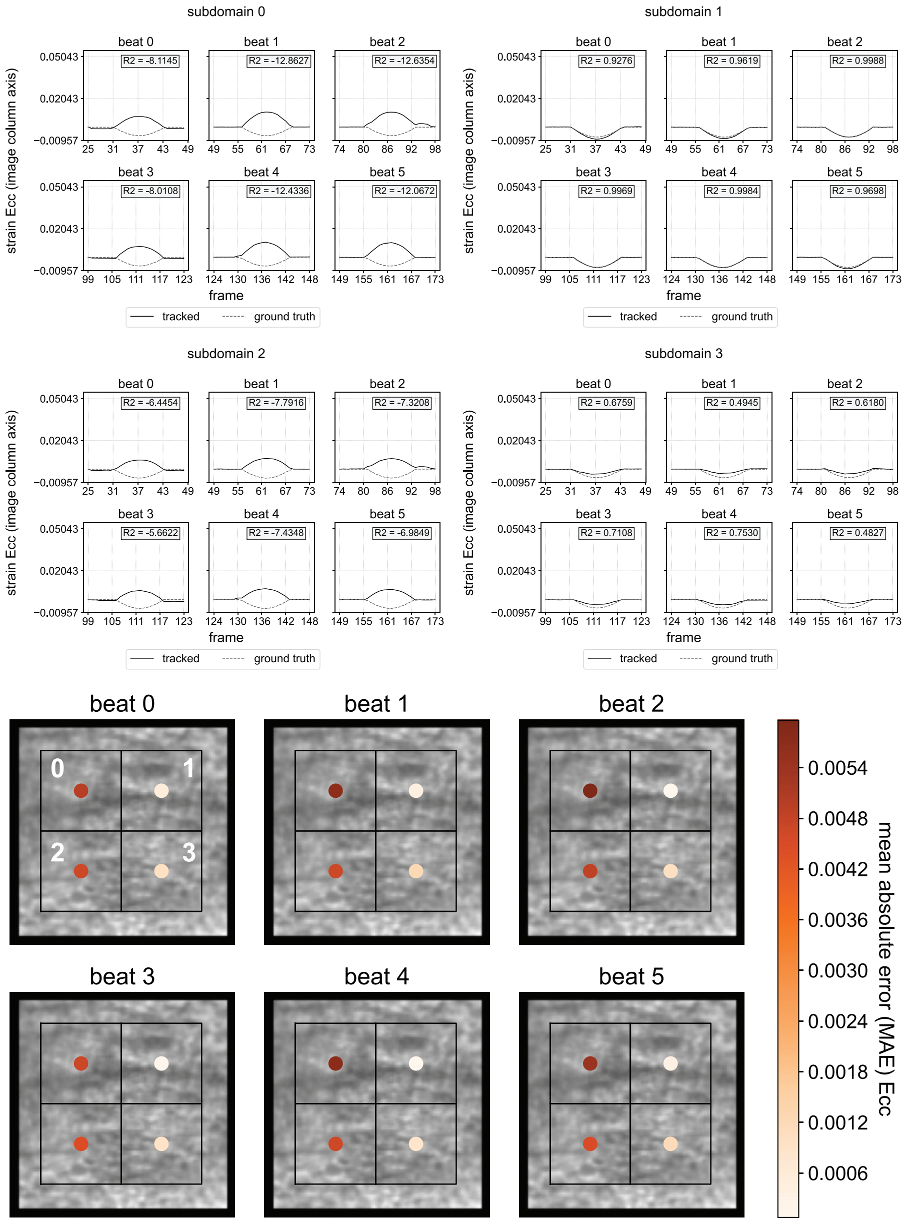

Fig S1_8. Error in E_{cc} strain for ``SyntheticTextures_3''. The numbers $0, 1, 2, 3$ shown on beat 0 MAE plot indicate the subdomain number.

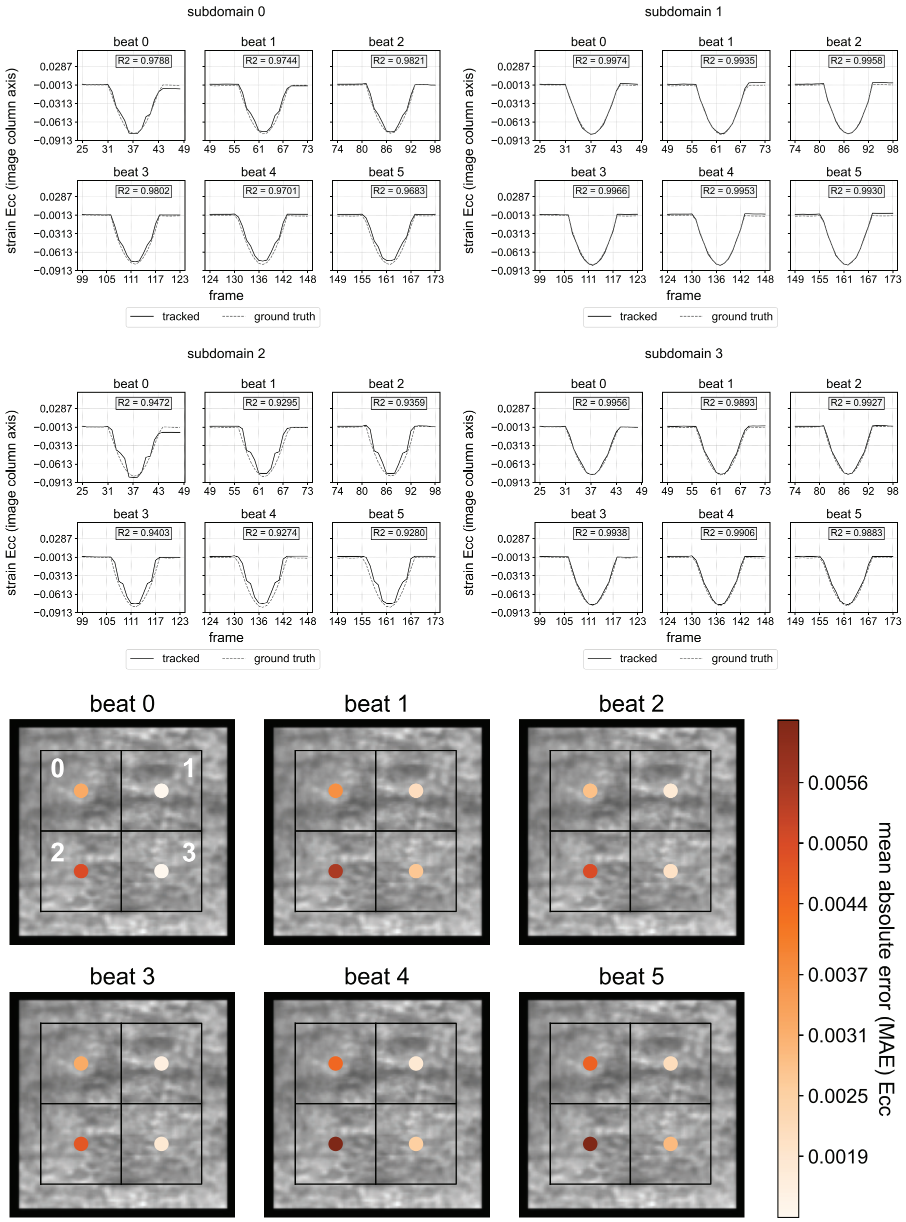

Fig S1_9. Error in E_{cc} strain for ``SyntheticTextures_5''. The numbers $0, 1, 2, 3$ shown on beat 0 MAE plot indicate the subdomain number.

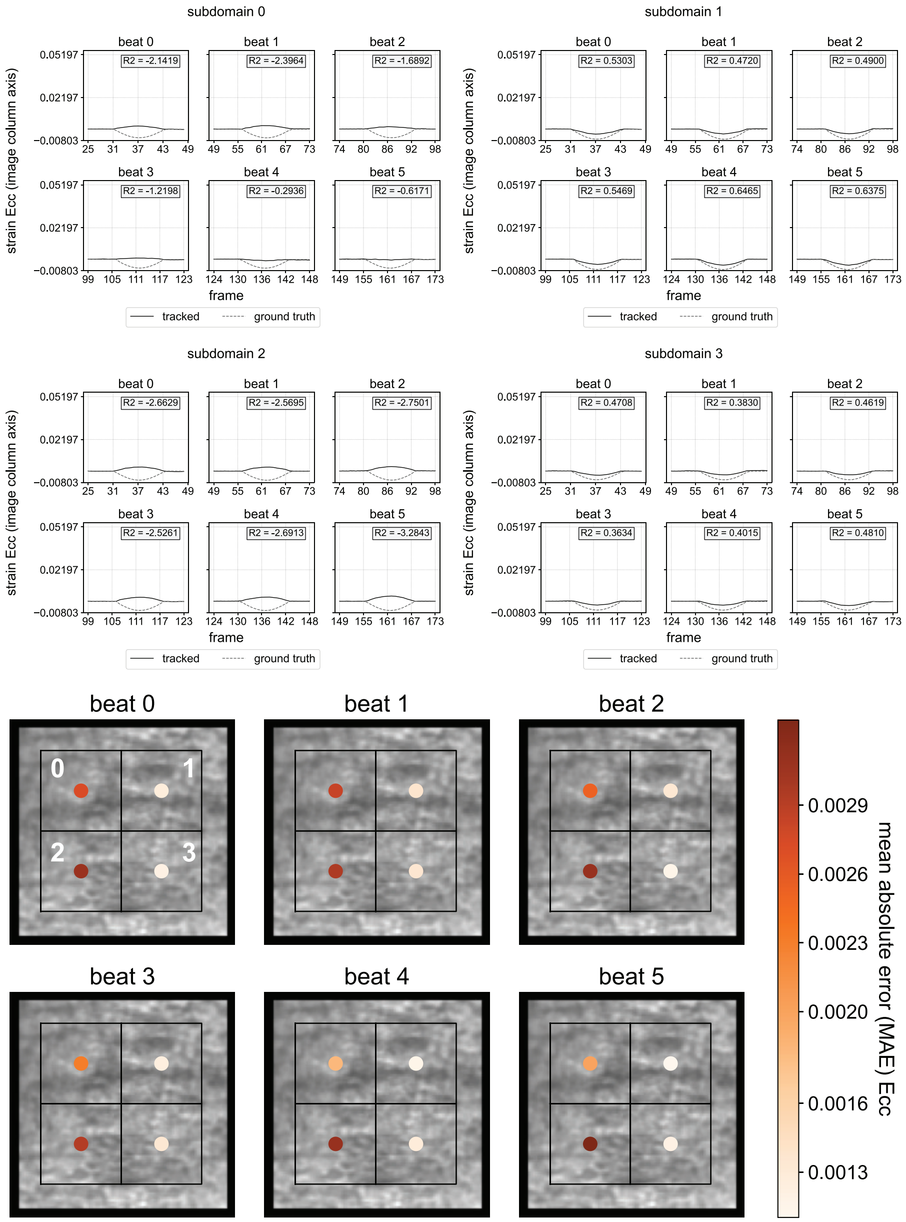

Fig S1_10. Error in E_{cc} strain for ``SyntheticTextures_7''. The numbers $0, 1, 2, 3$ shown on beat 0 MAE plot indicate the subdomain number.

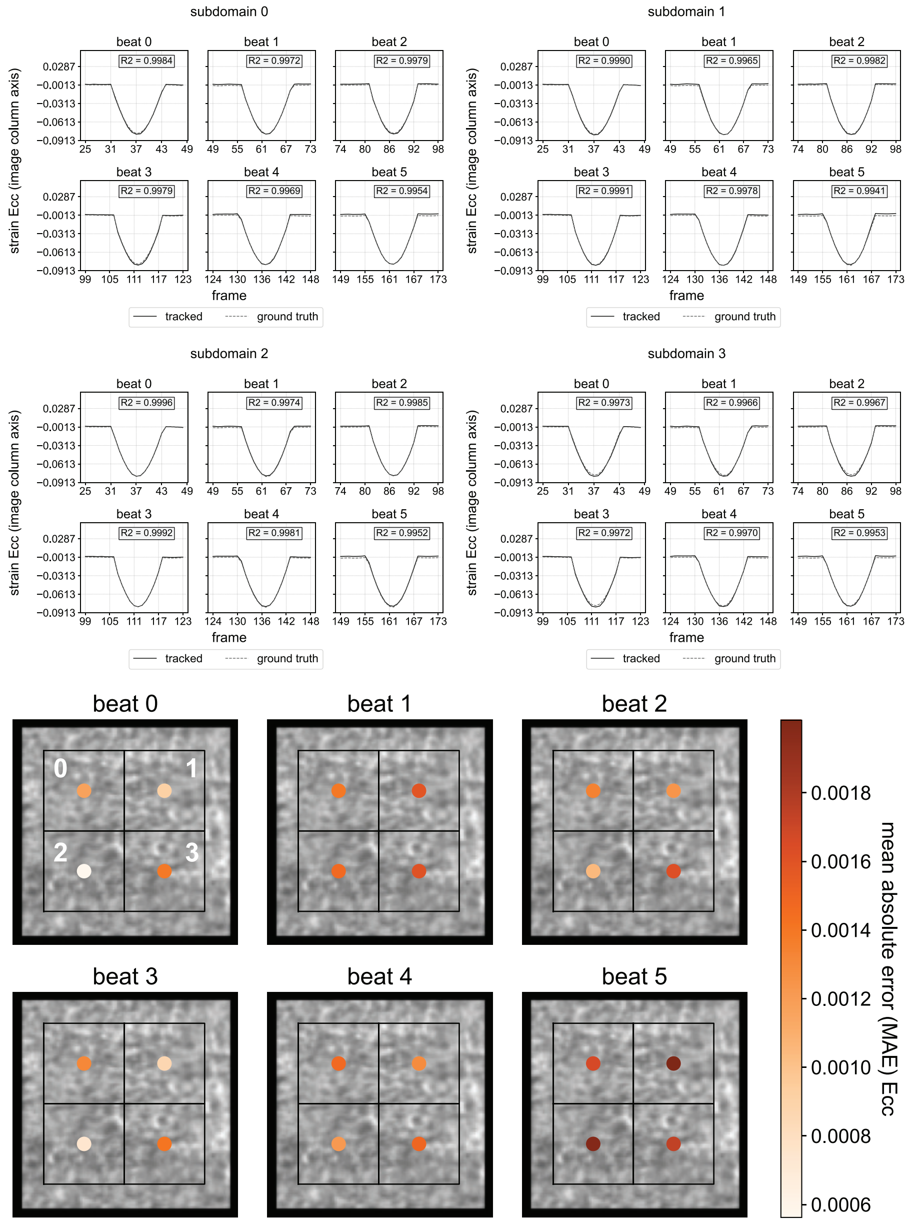

Fig S1_11. Error in E_{cc} strain for ``SyntheticTextures_14''. The numbers $0, 1, 2, 3$ shown on beat 0 MAE plot indicate the subdomain number.

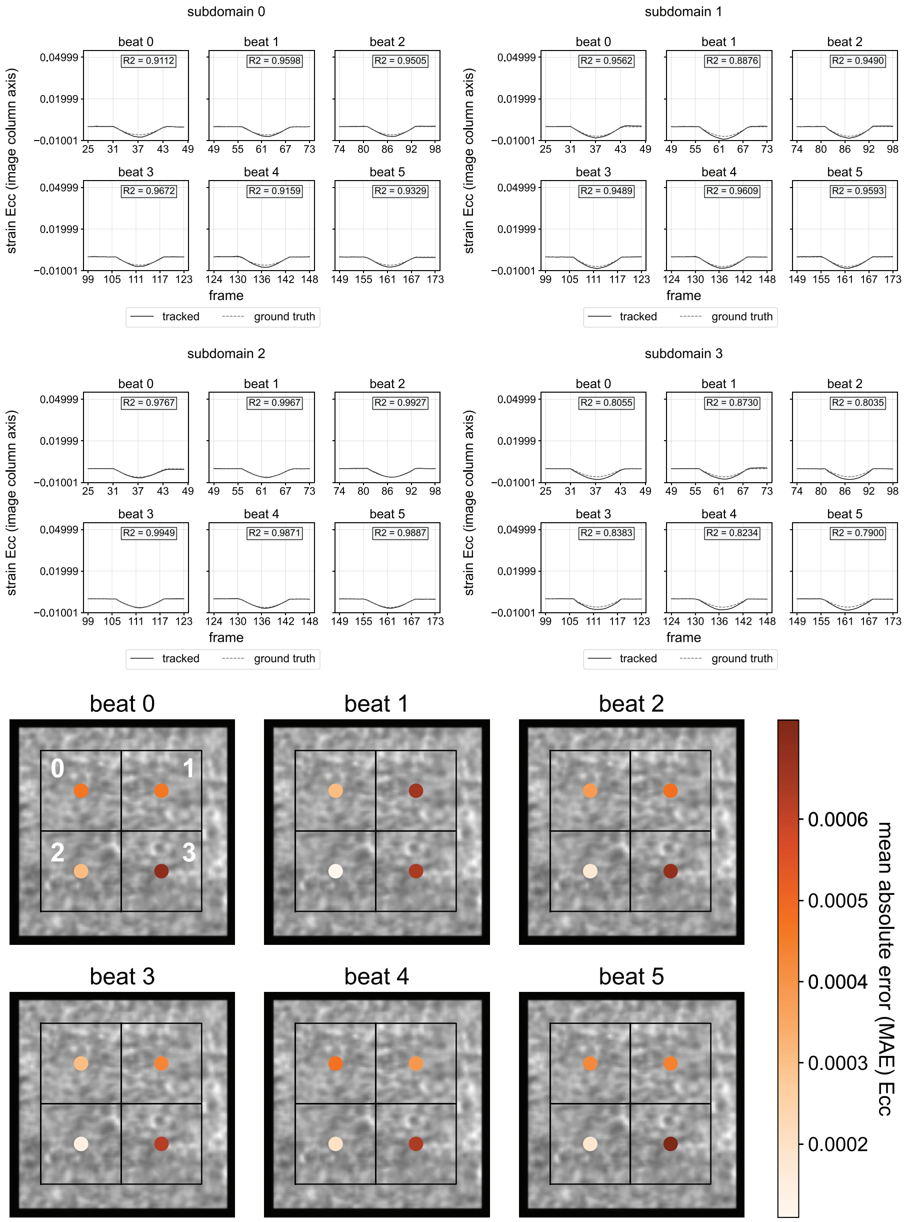

Fig S1_12. Error in E_{cc} strain for ``SyntheticTextures_16''. The numbers $0, 1, 2, 3$ shown on beat 0 MAE plot indicate the subdomain number.

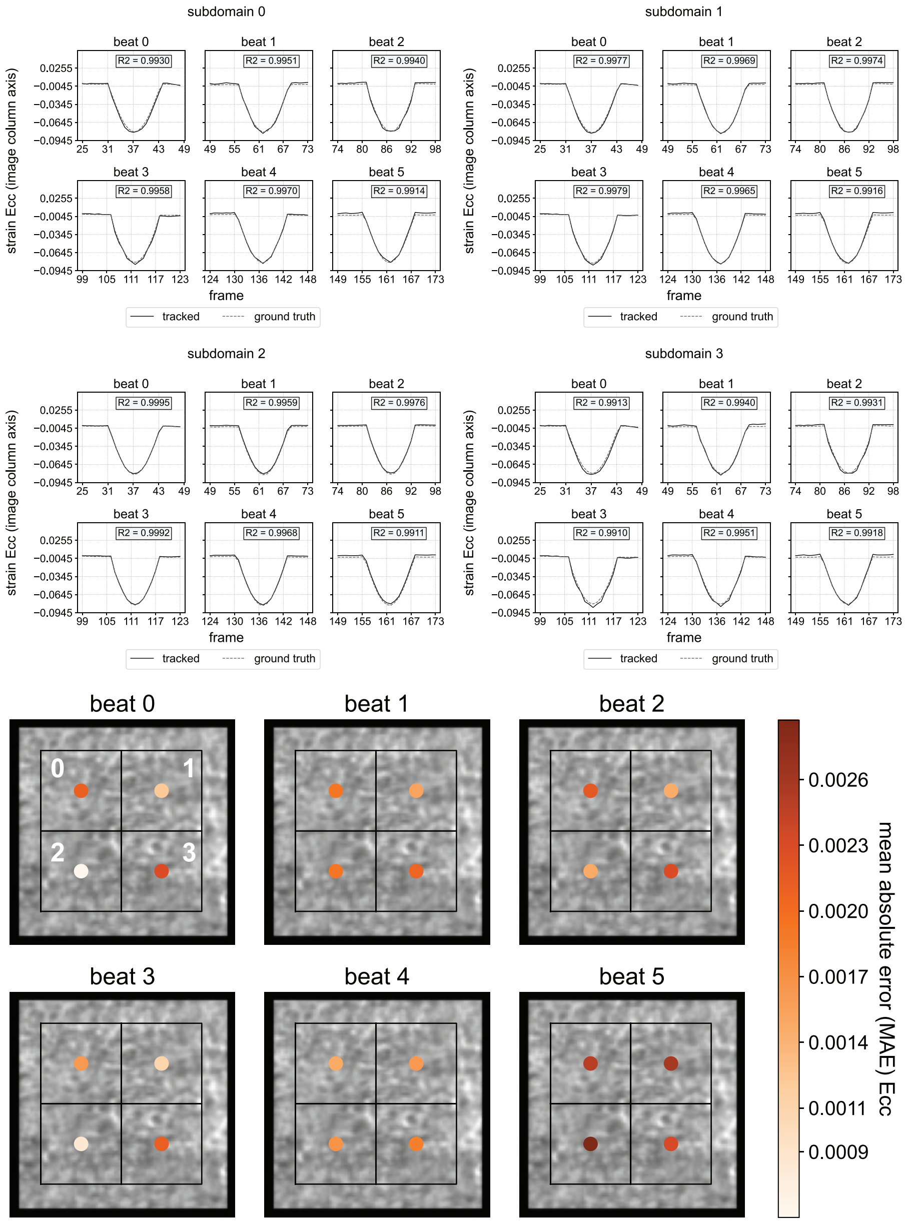

Fig S1_13. Error in E_{cc} strain for ``SyntheticTextures_18''. The numbers $0, 1, 2, 3$ shown on beat 0 MAE plot indicate the subdomain number.

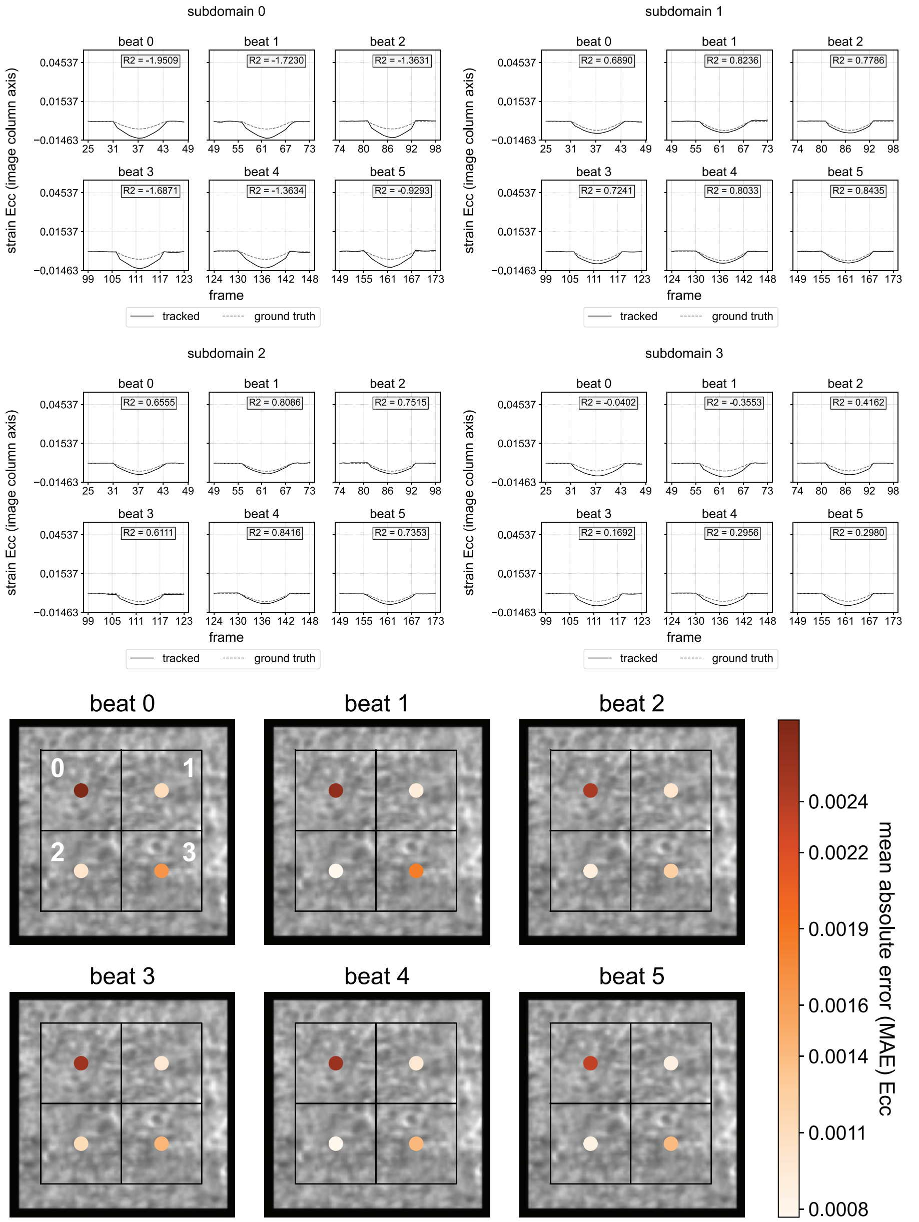

Fig S1_14. Error in E_{cc} strain for ``SyntheticTextures_20''. The numbers $0, 1, 2, 3$ shown on beat 0 MAE plot indicate the subdomain number.

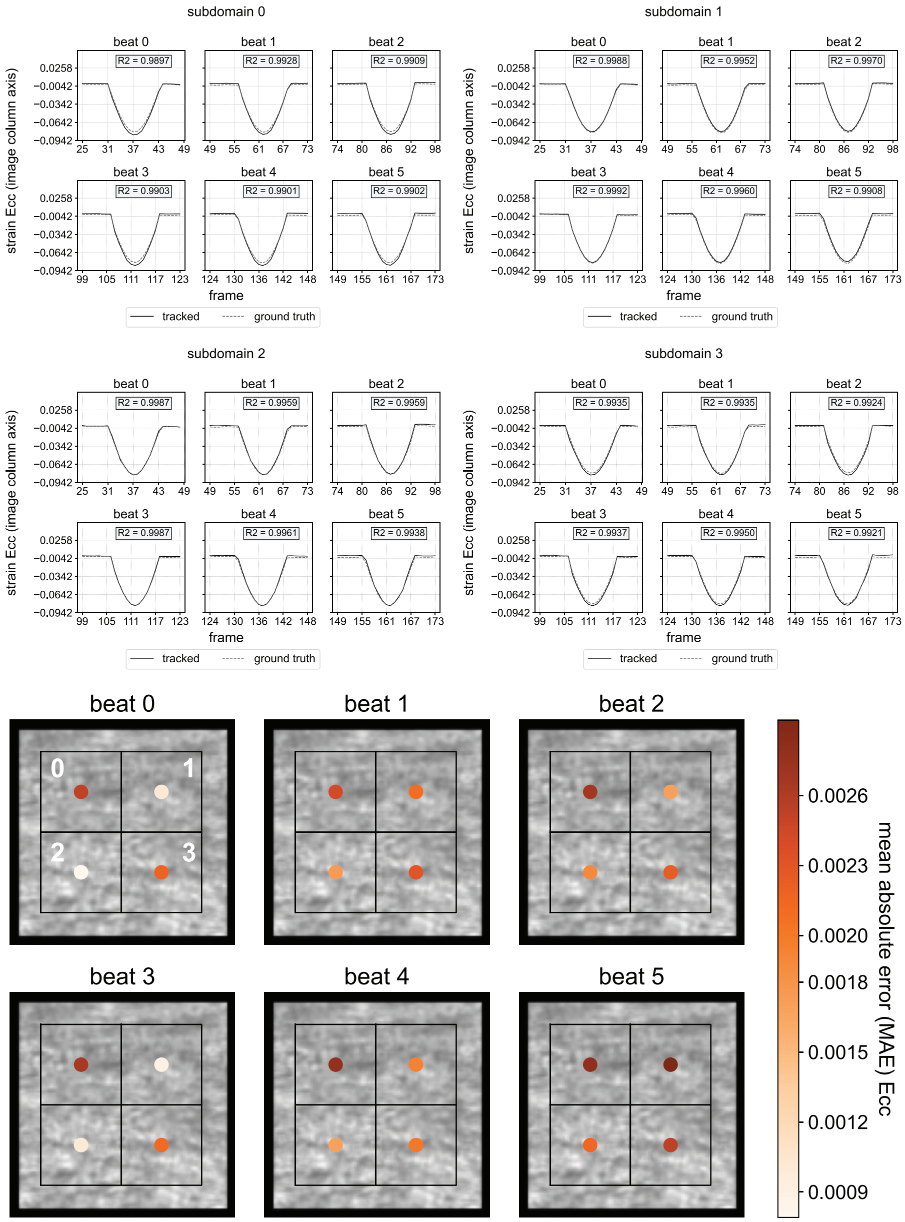

Fig S1_15. Error in E_{cc} strain for ``SyntheticTextures_29''. The numbers $0, 1, 2, 3$ shown on beat 0 MAE plot indicate the subdomain number.

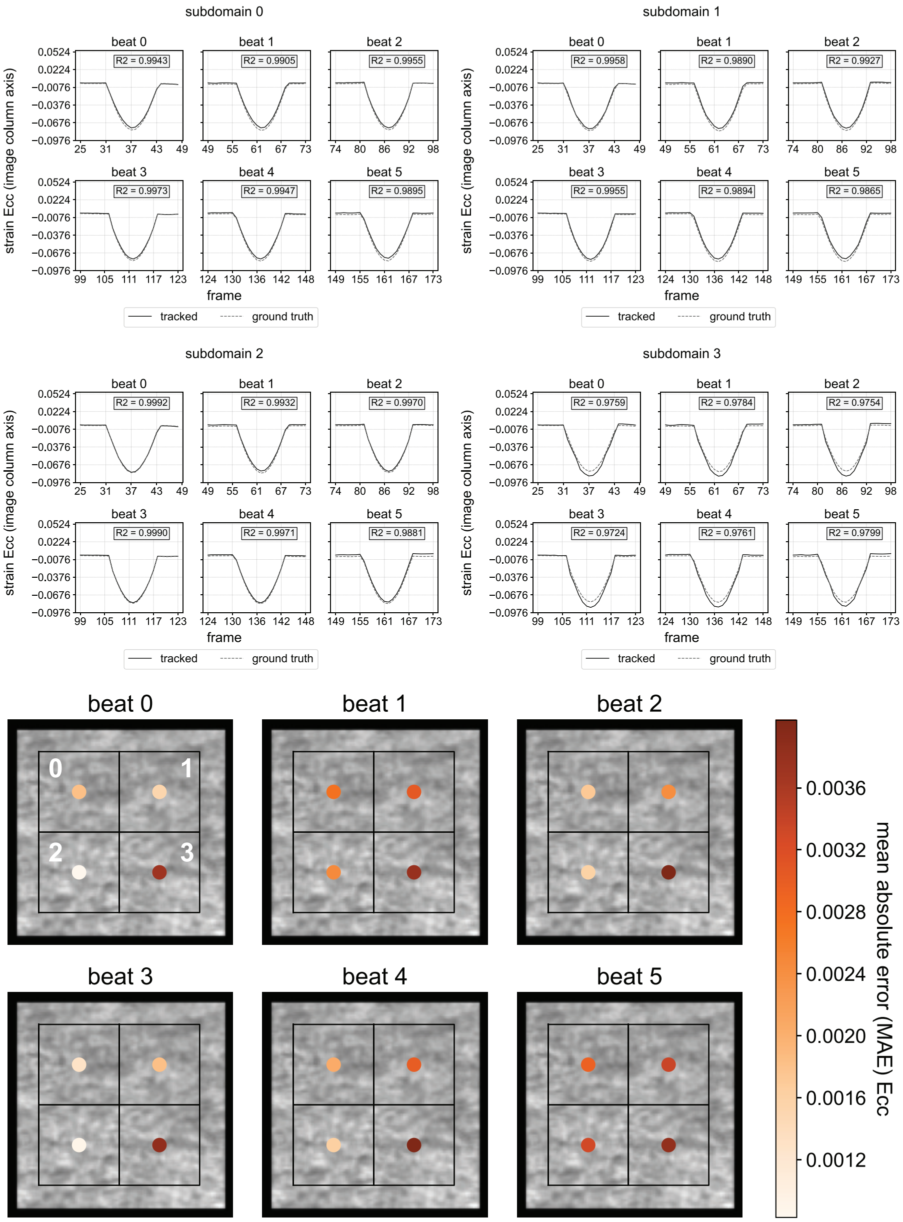

Fig S1_16. Error in E_{cc} strain for ``SyntheticTextures_33''. The numbers $0, 1, 2, 3$ shown on beat 0 MAE plot indicate the subdomain number.

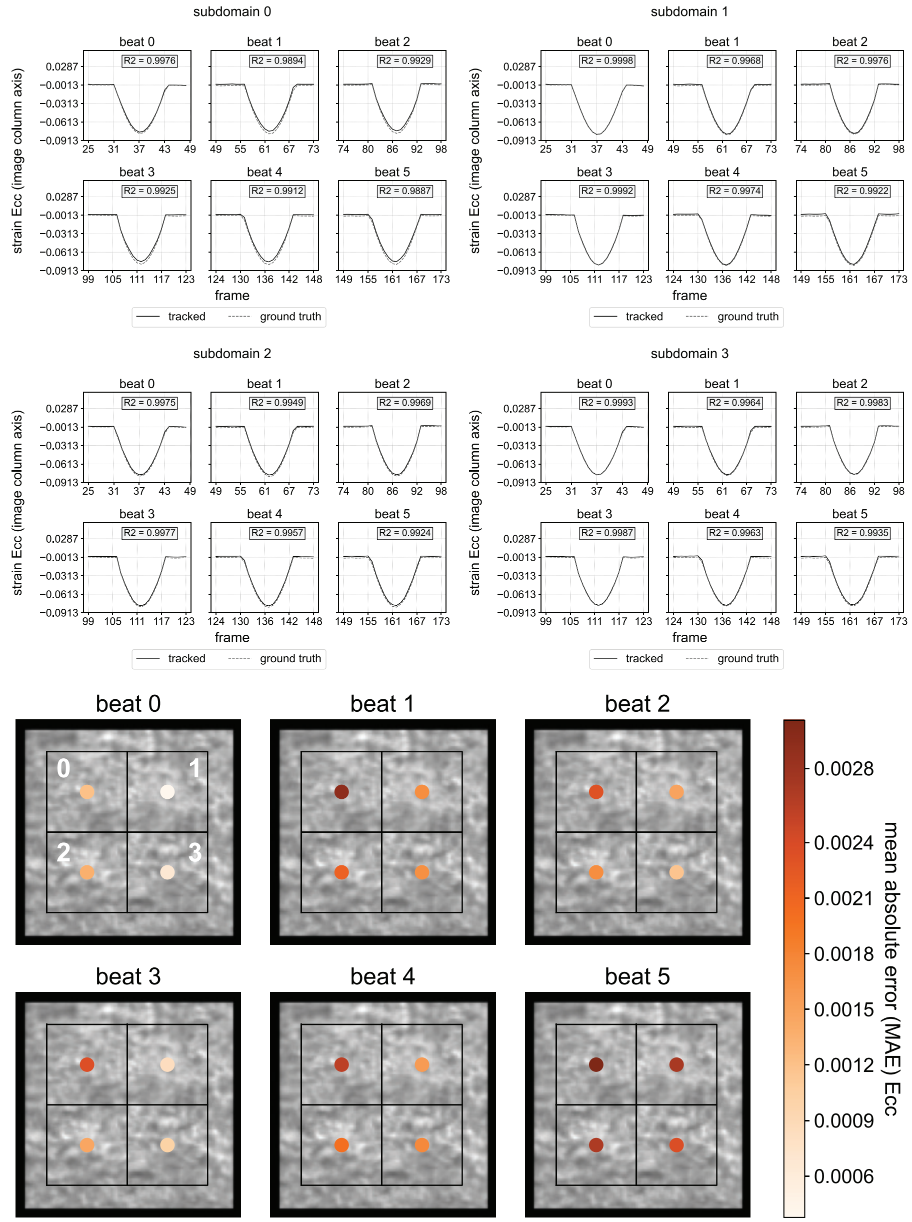

Fig S1_17. Error in E_{cc} strain for ``SyntheticTextures_42''. The numbers 0, 1, 2, 3 shown on beat 0 MAE plot indicate the subdomain number.

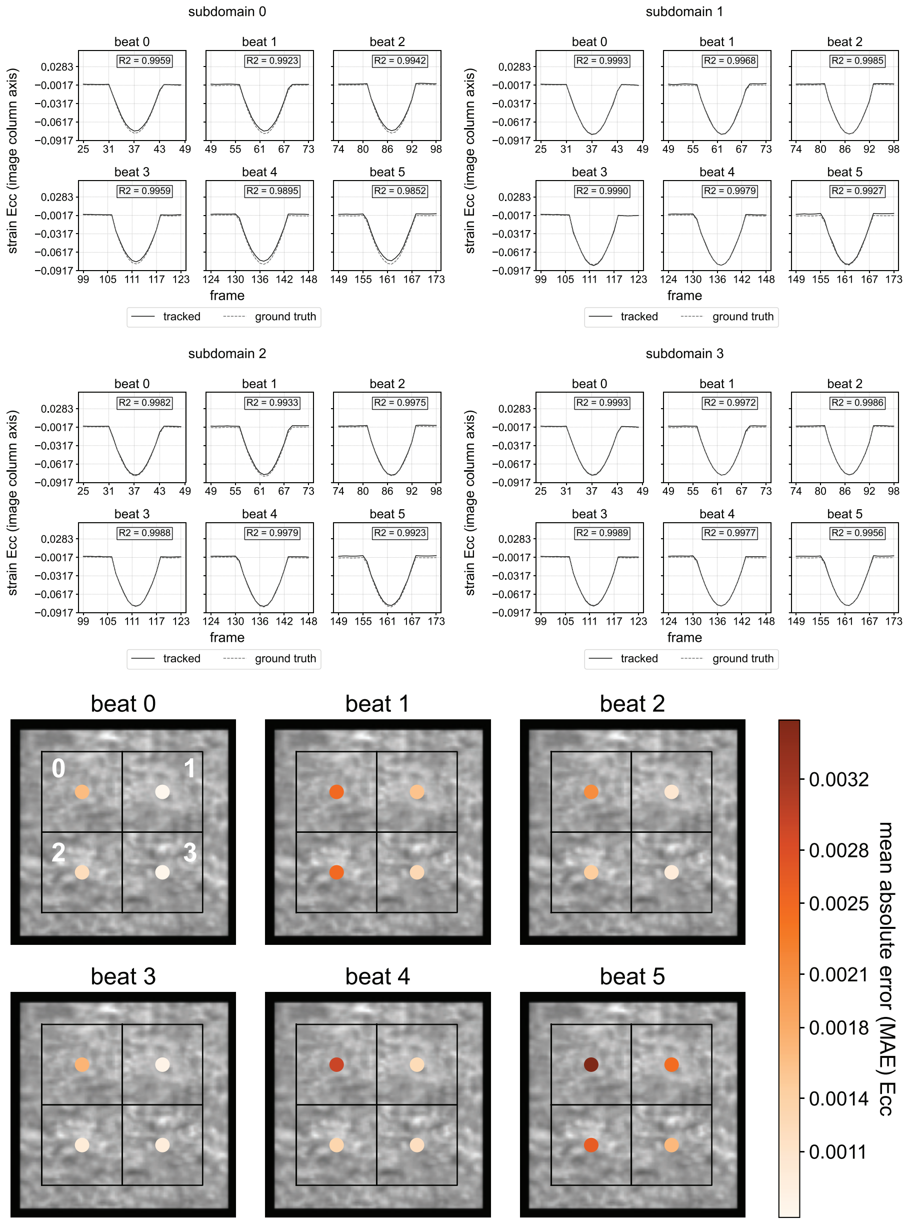

Fig S1_18. Error in E_{cc} strain for ``SyntheticTextures_46''. The numbers $0, 1, 2, 3$ shown on beat 0 MAE plot indicate the subdomain number.

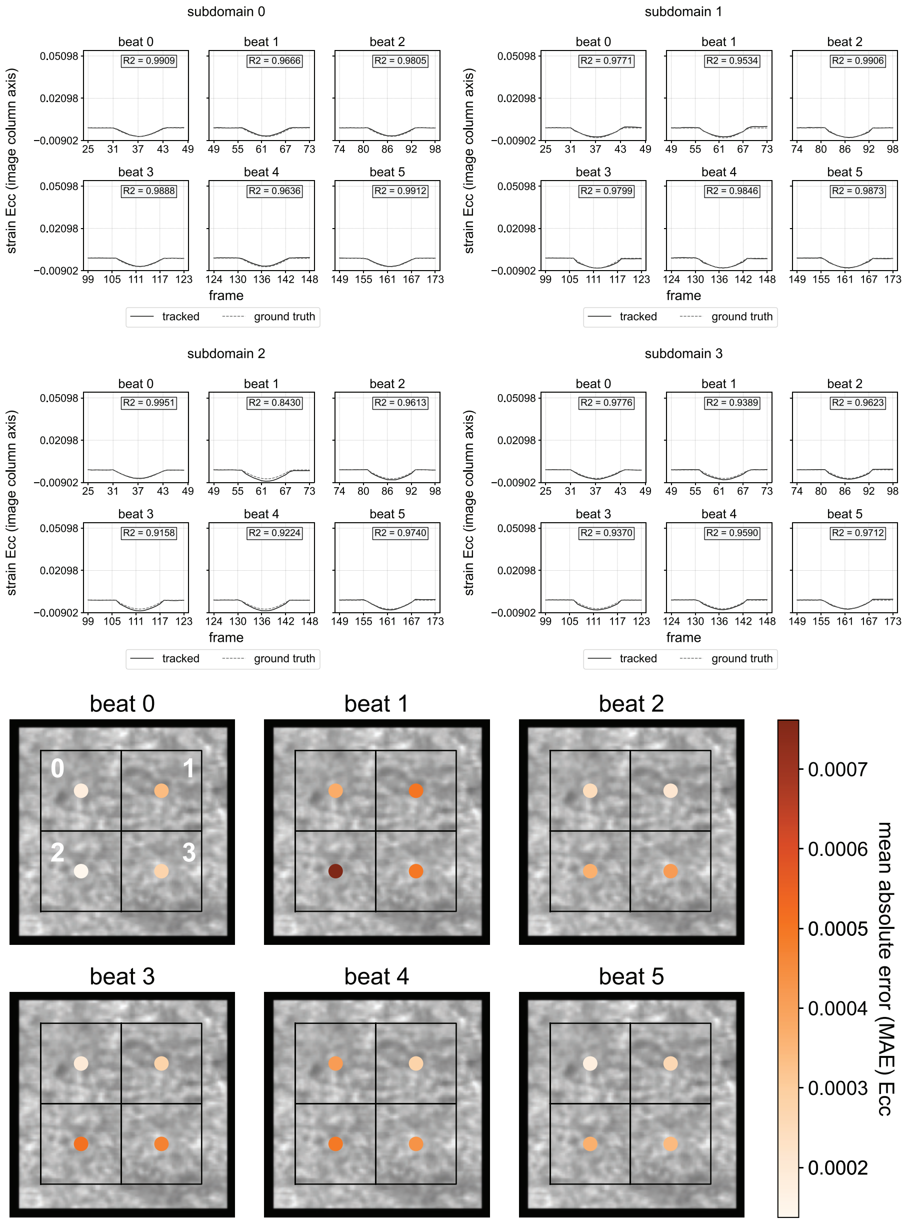

Fig S1_19. Error in E_{cc} strain for ``SyntheticTextures_51''. The numbers $0, 1, 2, 3$ shown on beat 0 MAE plot indicate the subdomain number.

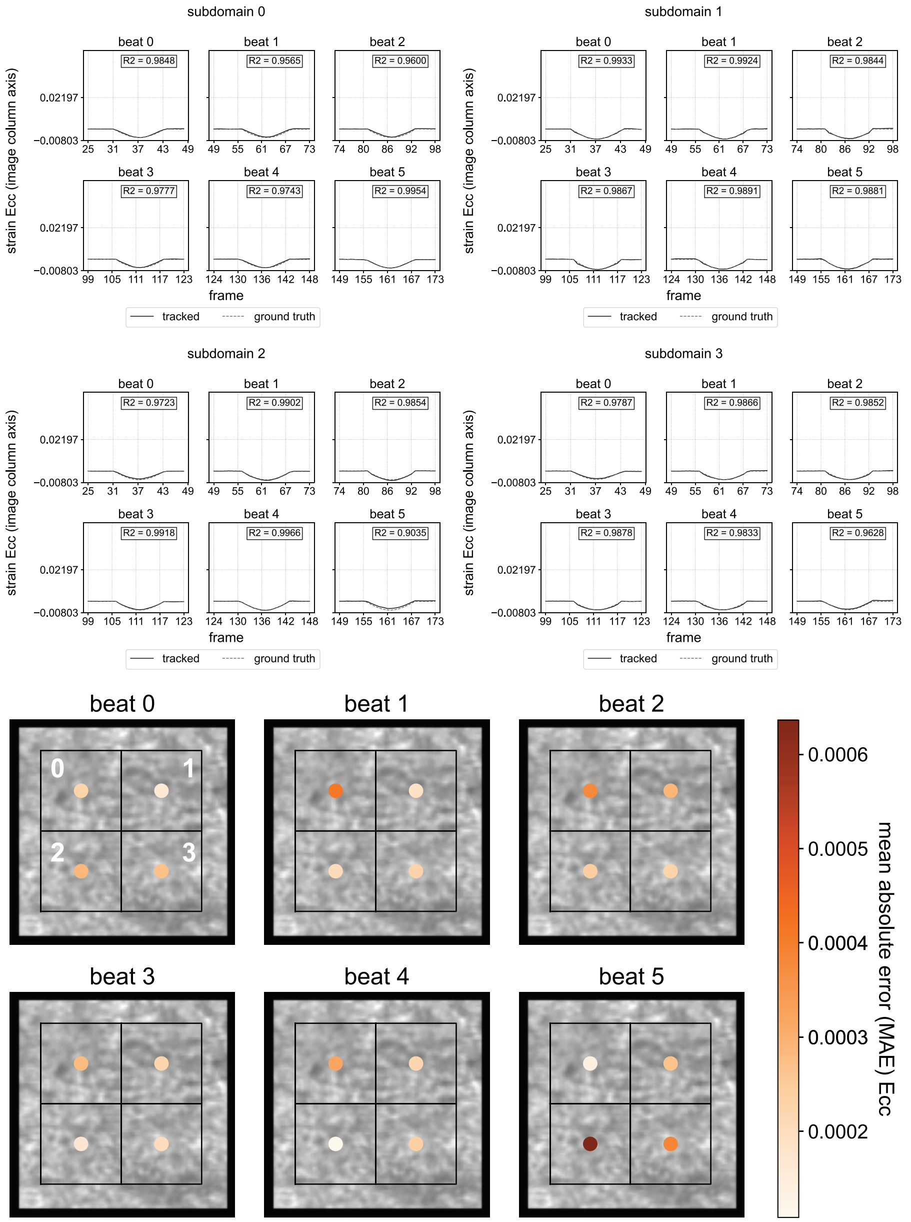

Fig S1_20. Error in E_{cc} strain for ``SyntheticTextures_52''. The numbers $0, 1, 2, 3$ shown on beat 0 MAE plot indicate the subdomain number.

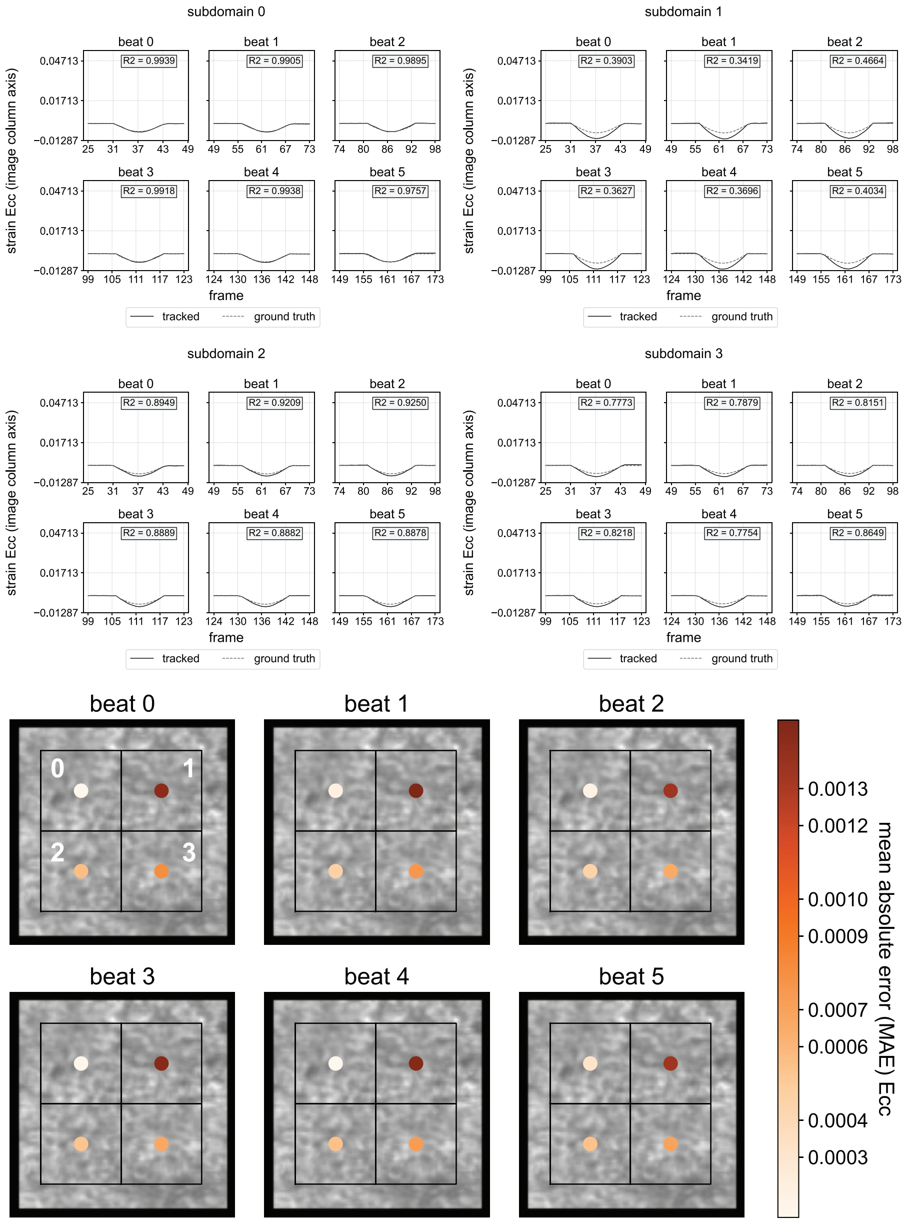

Fig S1_21. Error in E_{cc} strain for ``SyntheticTextures_55''. The numbers $0, 1, 2, 3$ shown on beat 0 MAE plot indicate the subdomain number.

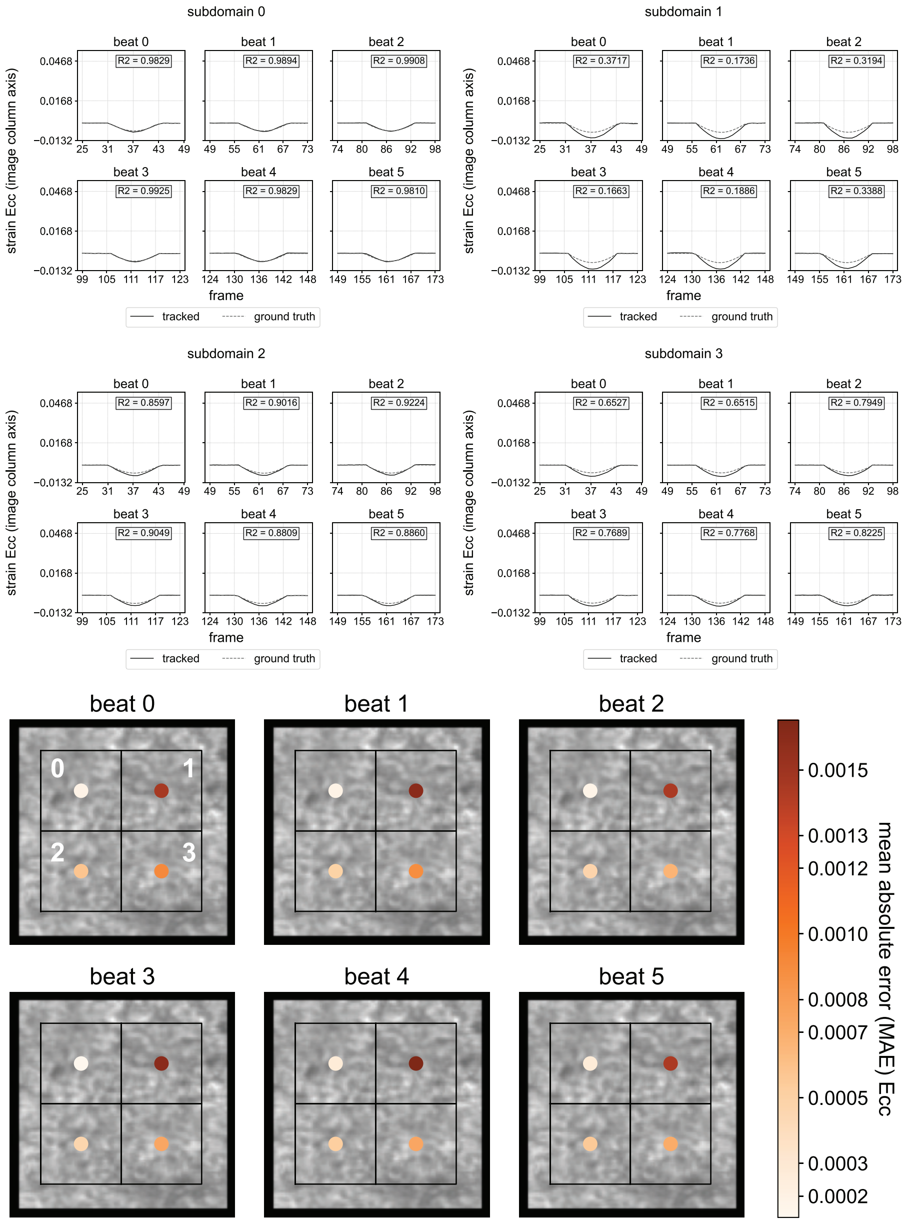

Fig S1_22. Error in E_{cc} strain for ``SyntheticTextures_56''. The numbers $0, 1, 2, 3$ shown on beat 0 MAE plot indicate the subdomain number.

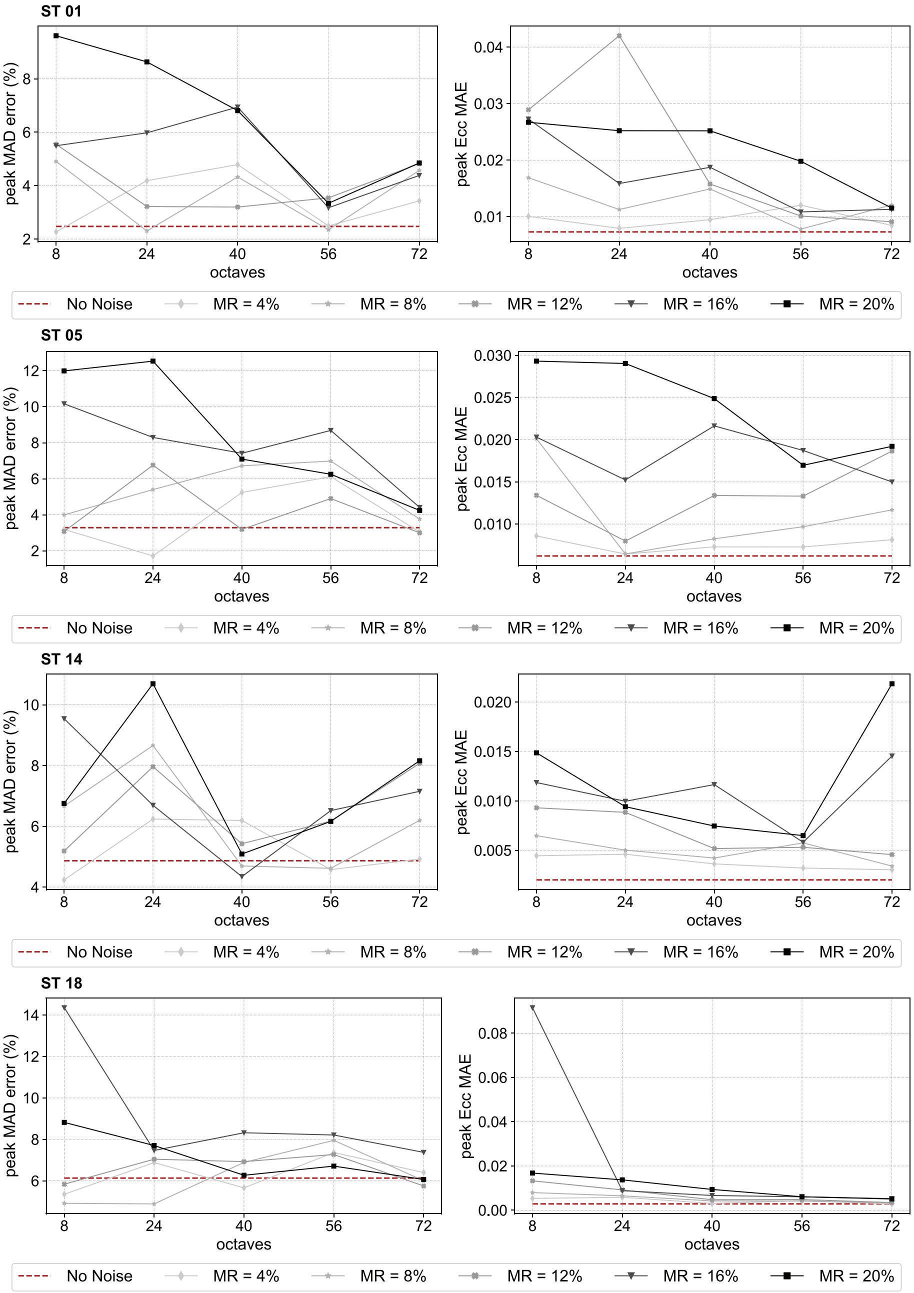
ST 01
peak MAD error (%)
peak Ecc MAE
octaves
No Noise
MR = 4%
MR = 8%
MR = 12%
MR = 16%
MR = 20%
ST 05
ST 14
ST 18

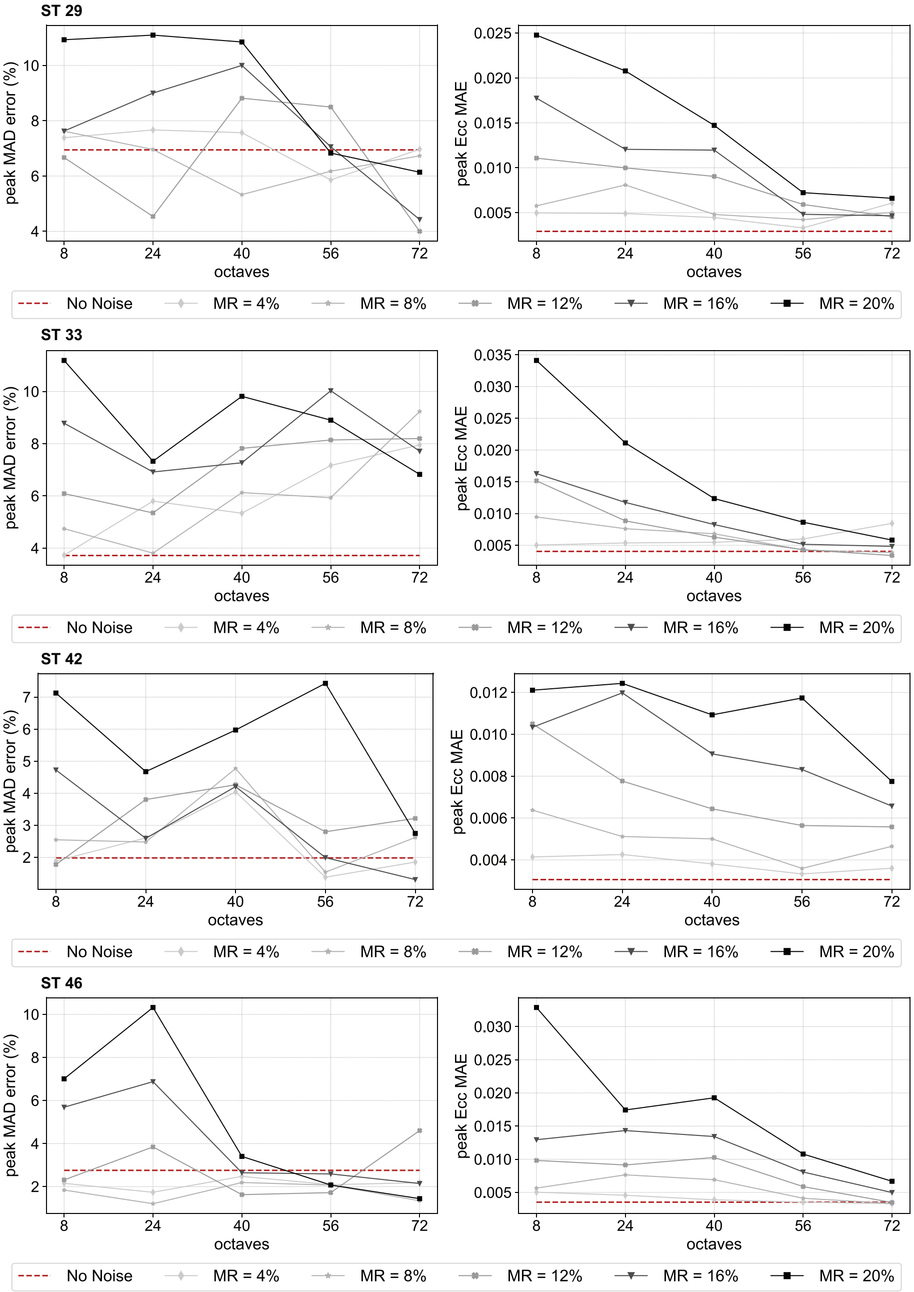

Fig S1_23. Results of our pipeline validation against noisy synthetic data of "Type 1" based on FE simulations with homogeneous activation for different Perlin noise octaves and magnitude ratios (MR).

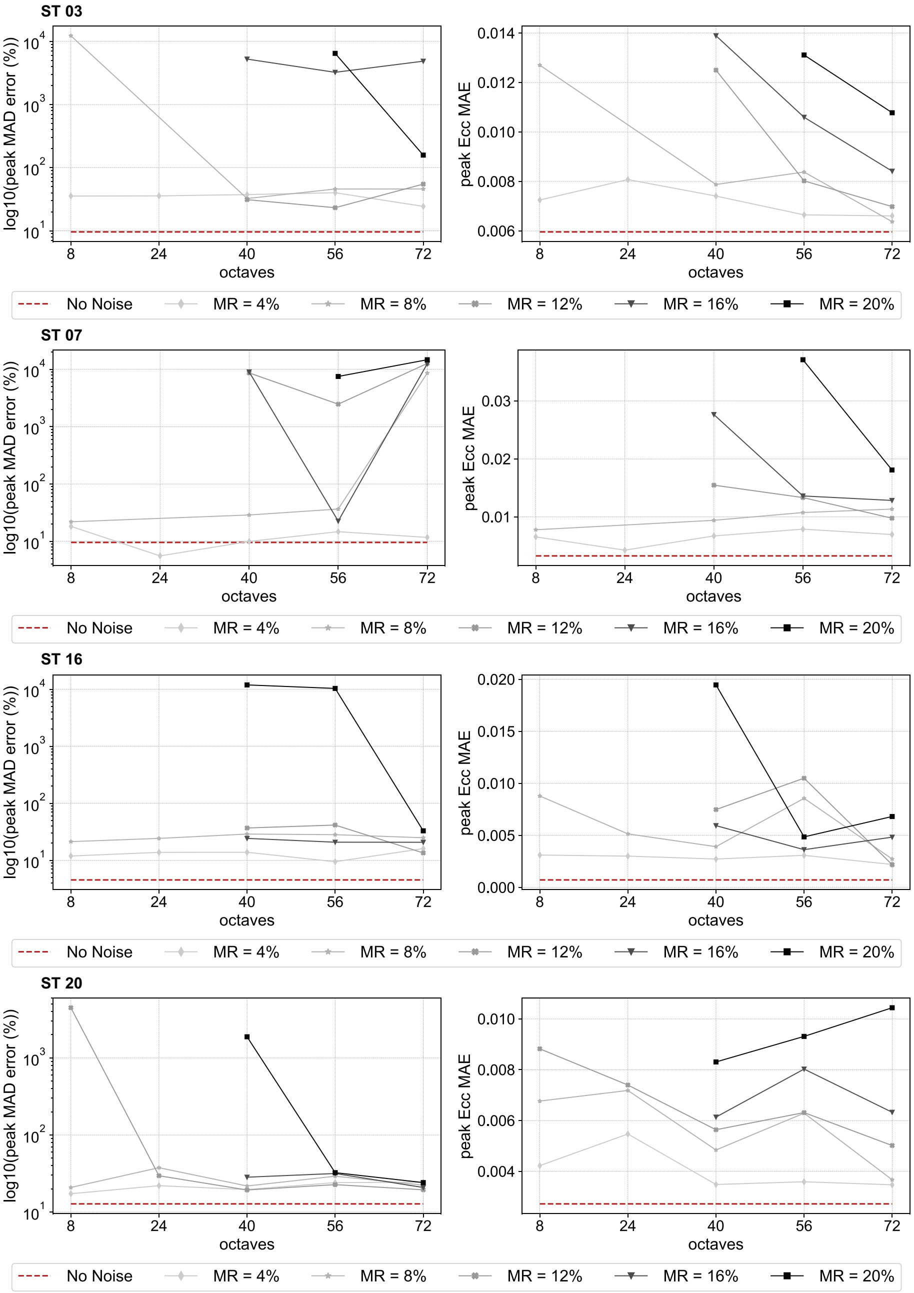

ST 03
ST 07
ST 16
ST 20
log10(peak MAD error (%))
peak Ecc MAE
octaves
8
24
40
56
72
No Noise
MR = 4%
MR = 8%
MR = 12%
MR = 16%
MR = 20%

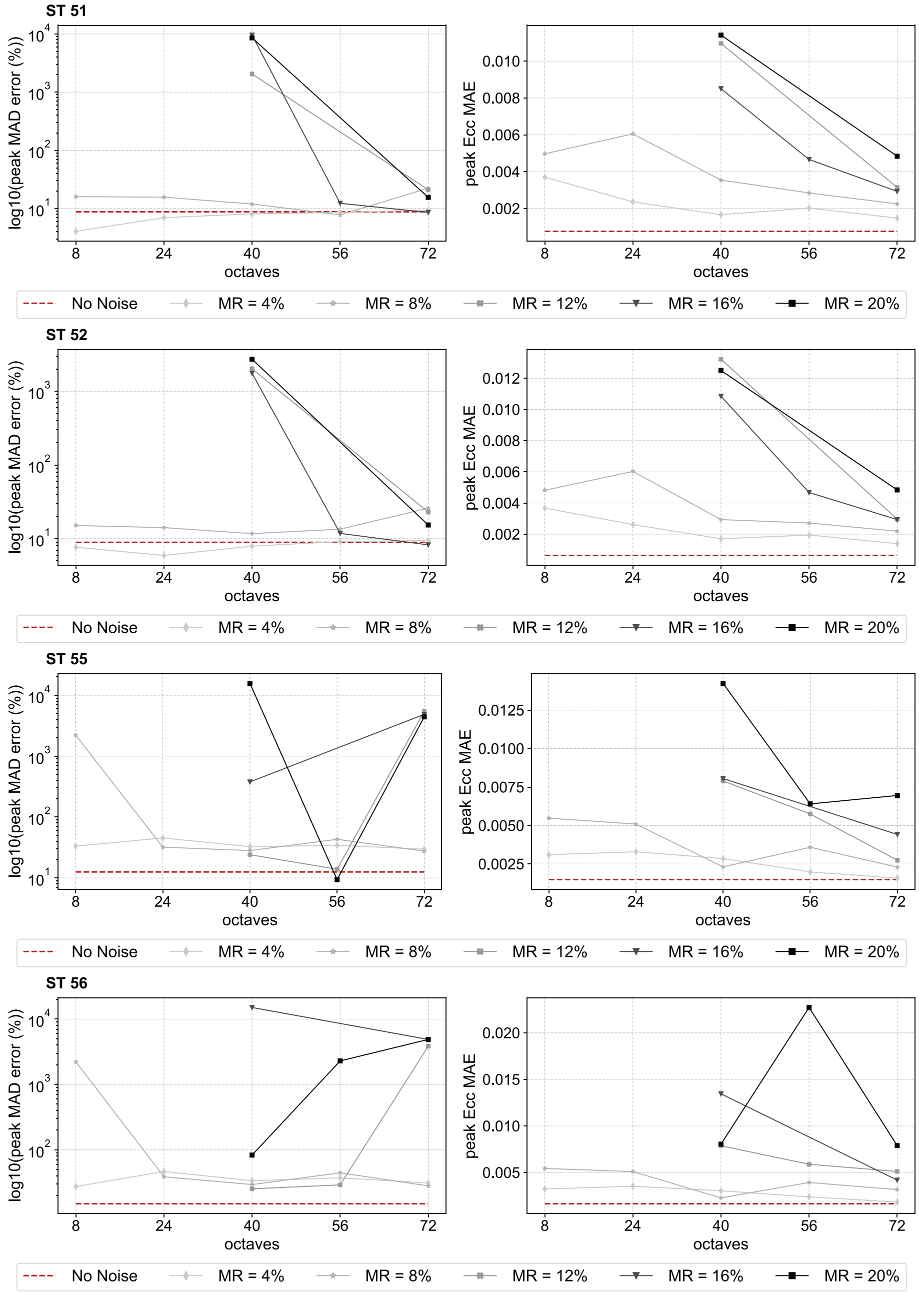

Fig S1_24. Results of our pipeline validation against noisy synthetic data of "Type 1" based on FE simulations with heterogeneous activation for different Perlin noise octaves and magnitude ratios (MR).

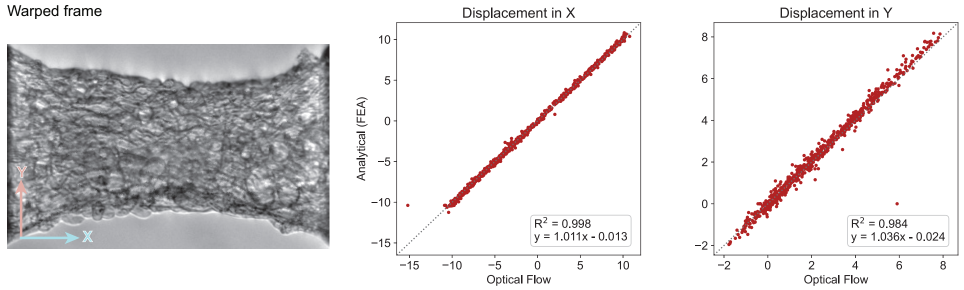

Fig S1_25. Results of our pipeline validation against synthetic data of "Type 2" for displacement outputs.

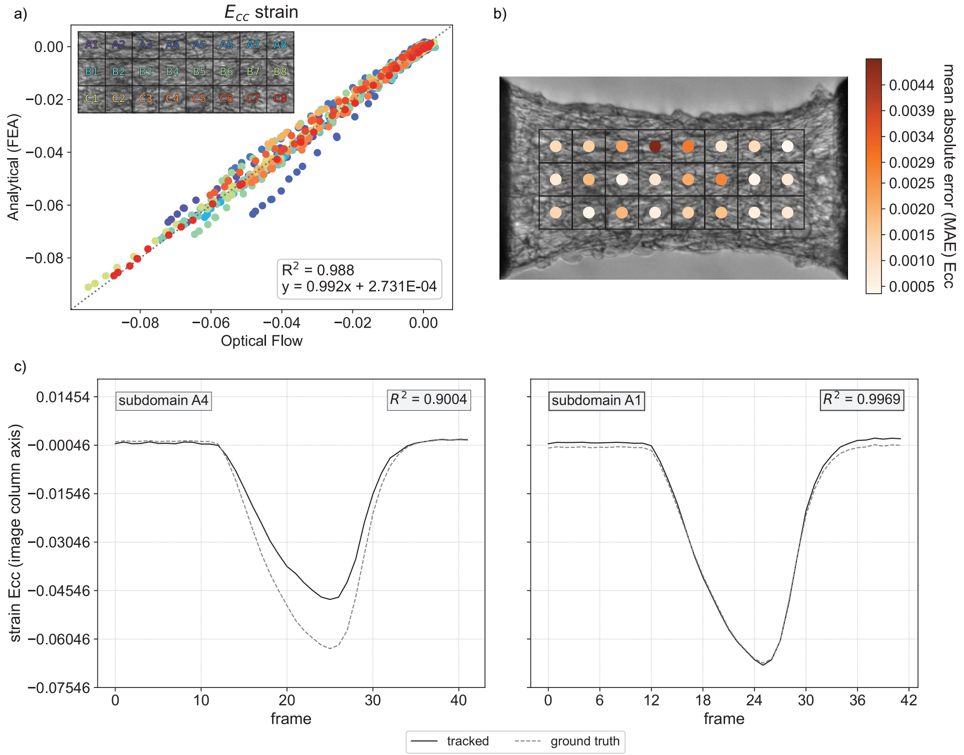

Fig S1_26. Results of our pipeline validation against synthetic data of "Type 2" for E_{cc} strain outputs: a) E_{cc} strains obtained analytically and by tracking are in good agreement across all subdomains; (b) the mean absolute errors in E_{cc} are below 0.0049 in all subdomains; (c) with the maximum error occurring in subdomain A4 and the median error occurring in subdomain A1.

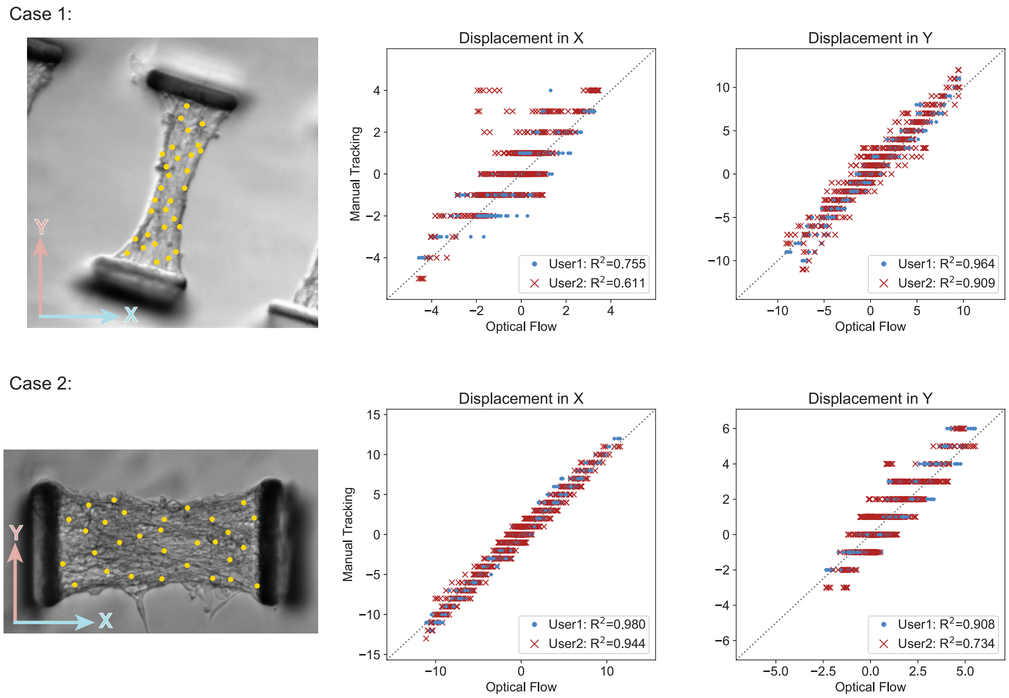

Fig S1_27. Results of our pipeline validation against manually tracked points for 2 different cases of "Type 2" data.

References

1. Geuzaine C, Remacle JF. Gmsh: A 3-D finite element mesh generator with built-in pre-and post-processing facilities. International journal for numerical methods in engineering. 2009;79(11):1309–1331. doi:https://doi.org/10.1002/nme.2579.

2. Javor J, Sundaram S, Chen CS, Bishop DJ. A Microtissue Platform to Simultaneously Actuate and Detect Mechanical Forces via Non-Contact Magnetic Approach. Journal of Microelectromechanical Systems. 2021;30(1):96–104. doi:10.1109/JMEMS.2020.3036978.

3. Alnæs M, Blechta J, Hake J, Johansson A, Kehlet B, Logg A, et al. The FEniCS project version 1.5. Archive of Numerical Software. 2015;3(100):9–23.

4. Logg A, Mardal KA, Wells G. Automated solution of differential equations by the finite element method: The FEniCS book. vol. 84. Germany: Springer Science & Business Media; 2012.

5. Pezzuto S, Ambrosi D, Quarteroni A. An orthotropic active–strain model for the myocardium mechanics and its numerical approximation. European Journal of Mechanics - A/Solids. 2014;48:83–96. doi:https://doi.org/10.1016/j.euromechsol.2014.03.006.

6. Gurev V, Pathmanathan P, Fattebert JL, Wen HF, Magerlein J, Gray RA, et al. A high-resolution computational model of the deforming human heart.

Biomechanics and modeling in mechanobiology. 2015;14:829–849. doi:https://doi.org/10.1007/s10237-014-0639-8.

7. Finsberg H, Xi C, Tan JL, Zhong L, Genet M, Sundnes J, et al. Efficient estimation of personalized biventricular mechanical function employing gradient-based optimization. International journal for numerical methods in biomedical engineering. 2018;34(7):e2982.

8. Holzapfel GA. Nonlinear solid mechanics: a continuum approach for engineering science; 2002.

9. Weiss JA, Maker BN, Govindjee S. Finite element implementation of incompressible, transversely isotropic hyperelasticity. Computer Methods in Applied Mechanics and Engineering. 1996;135(1):107–128. doi:https://doi.org/10.1016/0045-7825(96)01035-3.

10. Hood P, Taylor C. Navier-Stokes equations using mixed interpolation. Finite element methods in flow problems. 1974; p. 121–132.

11. Ambrosi D, Pezzuto. Active stress vs. active strain in mechanobiology: constitutive issues. Journal of Elasticity. 2012;107:199–212. doi:https://doi.org/10.1007/s10659-011-9351-4.

12. Zhivomirov H. A method for colored noise generation. Romanian journal of acoustics and vibration. 2018;15(1):14–19.

13. van der Walt S, Schönberger JL, Nunez-Iglesias J, Boulogne F, Warner JD, Yager N, et al. scikit-image: image processing in Python. PeerJ. 2014;2:e453. doi:10.7717/peerj.453.

14. Perlin K. An Image Synthesizer. Siggraph Computer Graphics. 1985;19(3):287–296. doi:10.1145/325165.325247.

15. Jilberto J, DePalma SJ, Lo J, Kobeissi H, Lejeune E, Baker BM, et al. A data–driven computational modeling for engineered cardiac microtissues. Acta Biomaterialia. 2023. doi: https://doi.org/10.1016/j.actbio.2023.10.025.
