# Supplementary material for: MicroBundleCompute: Automated segmentation, tracking, and analysis of subdomain deformation in cardiac microbundles
Source: PLoS One. 2024 Mar 26;19(3):e0298863. doi: 10.1371/journal.pone.0298863 (PMC10965069; doi:10.1371/journal.pone.0298863)
Supplement: S2 Appendix — The results of implementing “MicroBundleCompute” on a total of 24 experimental time-lapse images of cardiac microbundles, 11 examples of “Type 1,” 7 of “Type 2,” and 6 of “Type 3.” Table S2_1. A summary of the experimental conditions associated with each example movie. The details of experimental conditions include image acquisition parameters as well as pillar stiffness values for “Type 1” and “Type 2” data. Table S2_2. Additional details for each example of “Type 1” data. These details include example-specific information, code implementation details, and subdomain segmentation parameters. Table S2_3. Additional details for each example of “Type 2” data. These details include example-specific information, code implementation details, and subdomain segmentation parameters. Table S2_4. Additional details for each example of “Type 3” data. These details include example-specific information and subdomain segmentation parameters. Fig S2_1. Example outputs of “MicroBundleCompute” run on “Type 1” experimental data. The provided outputs include full-field mean absolute displacement and subdomain-averaged Green-Lagrange Ecc strain at the first tracked peak, as well as a time series plot of Ecc strain for the first tracked beat. Fig S2_2. Example outputs of “MicroBundleCompute” run on “Type 2” experimental data. The provided outputs include full-field mean absolute displacement and subdomain-averaged Green-Lagrange Ecc strain at the first tracked peak, as well as a time series plot of Ecc strain for the first tracked beat. Fig S2_3. Example outputs of “MicroBundleCompute” run on “Type 3” experimental data. The provided outputs include full-field mean absolute displacement and subdomain-averaged Green-Lagrange Ecc strain at the first tracked peak, as well as a time series plot of Ecc strain for the first tracked beat. (PDF) [file pone.0298863.s002.pdf]

MicroBundleCompute: Automated segmentation, tracking, and analysis of subdomain deformation in cardiac microbundles

S2 Appendix: Additional examples

In this Supplementary Document, we (1) provide the details necessary to reproduce our analysis on all examples in the "Microbundle Time-lapse Dataset," and (2) show the results of running "MicroBundleCompute" on all of these data. In brief, the "Microbundle Time-lapse Dataset" contains 24 experimental time-lapse images of cardiac microbundles. The dataset is hosted under a CC0 open-source license on the Dryad Digital Repository [1]. Consistent with our description of the experimental dataset in the "Experimental data" Section of the main paper document, we categorize these data as "Type 1" (11 examples), "Type 2" (7 examples), and "Type 3" (6 examples). A brief metadata summary for each type is given in Table (Table S2_1). These details are also elaborated on in the "Experimental data" Section, and documented as metadata for the "Microbundle Time-lapse Dataset." In Tables S2_2, S2_3, and S2_4, we provide the implementation details (mask type, first frame adjustment, and subdomain segmentation parameters) necessary to reproduce all results. For the subdomain segmentation parameters, we only include those that were changed from the default values provided within the "run_code.py" file located in the "MicroBundleCompute" GitHub repository https://github.com/HibaKob/MicroBundleCompute. Finally, in Figs S2_1, S2_2, and S2_3 we show representative outputs from running our code: full-field mean absolute displacement at the first tracked peak, subdomain averaged Green-Lagrange E_{cc} (horizontal) strain at the first tracked peak, and time series plots of E_{cc} strain for the first tracked beat. These results not only show the versatility of our framework, but also showcase the information that can be obtained from these data.

Table S2_1. A summary of the experimental conditions associated with each example movie.

Data type	Imaging modality	Post-seeding imaging	Microscope	Camera	Imaging magnification	Imaging frequency	Pillar stiffness
1	Brightfield	Example specific	Nikon Eclipse Ti	Evolve EMCCD	4 µm/pixel	30 Hz	2.677 N/m
2	Brightfield	7 days	Zeiss LSM800	Axiocam 503	0.908 µm/pixel	65 Hz	0.41 N/m
3	Brightfield†	Example specific	Nikon Eclipse Ti	Evolve EMCCD	Example specific	30 Hz	N/A

We note that the symbol † for "Type 3" "Imaging modality" is used to indicate that Example 1 is obtained via phase contrast microscopy, whereas the remainder of the examples are obtained via brightfield microscopy.

Table S2_2. A summary of the example-specific information, code implementation details and subdomain segmentation parameters for each example of "Type 1" data.

Type 1 data				
	Example-specific information	Implementation details		Subdomain segmentation parameters
Example	Post-seeding imaging	Mask	Adjust first frame	shrink_row
1	5 days	Automatic	N/A	default value (0.1)
2	5 days	Manual	N/A	default value (0.1)
3	5 days	Manual	Frame 10	default value (0.1)
4	5 days	Manual	Frame 10	default value (0.1)
5	5 days	Manual	Frame 4	default value (0.1)
6	5 days	Automatic	Frame 8	default value (0.1)
7	7 days	Automatic	N/A	default value (0.1)
8	7 days	Automatic	N/A	default value (0.1)
9	7 days	Automatic	N/A	0.2
10	7 days	Automatic	N/A	0.25
11	7 days	Automatic	N/A	default value (0.1)

Table S2_3. A summary of the example-specific information, code implementation details, and subdomain segmentation parameters for each example of "Type 2" data.

Type 2 data								
	Example-specific information		Implementation details			Subdomain segmentation parameters		
Example	Matrix stiffness	Matrix alignment	Mask	Adjust first frame	Image processing	pillar_clip_fraction	clip_rows	shrink_row
1	Soft	Aligned	Automatic	N/A	N/A	0.35	default value (False)	default value (0.1)
2	Soft	Aligned	Automatic	Frame 10	N/A	0.35	default value (False)	default value (0.1)
3	Soft	Aligned	Automatic	Frame 15	N/A	0.35	default value (False)	default value (0.1)
4	Soft	Aligned	Manual	N/A	Sharpen filter	0.35	default value (False)	default value (0.1)
5	Soft	Aligned	Automatic	N/A	N/A	0.3	True	0.25
6	Stiff	Aligned	Manual	N/A	N/A	0.3	default value (False)	default value (0.1)
7	Stiff	Random	Manual	Frame 15	N/A	0.35	default value (False)	0.25

Table S2_4. A summary of the example-specific information and subdomain segmentation parameters for each example of "Type 3" data.

Type 3 data										
	Example-specific information			Subdomain segmentation parameters						
Example	Image magnification	Post-seeding imaging	Tissue length	pillar_clip_fraction	clip_rows	shrink_row	shrink_col	tile_dim_pix	manual_sub	sub_extents
1*	1.6 µm/pixel	4 days	~ 0.8 mm	0.32	True	default value (0.1)	default value (0.1)	default value (40)	default value (False)	default value (None)
2	2.67 µm/pixel	8 days	~ 1.2 mm	0.25	True	default value (0.1)	default value (0.1)	default value (40)	default value (False)	default value (None)
3	2.67 µm/pixel	8 days	~ 0.5 mm	0.25 (N/A)	default value (False)	0.0	0.0	default value (40)	True	[255,295,170,427]
4	4 µm/pixel	8 days	~ 2 mm	0.25	True	default value (0.1)	default value (0.1)	default value (40)	default value (False)	default value (None)
5	2.67 µm/pixel	9 days	~ 1 mm	0.25	default value (False)	default value (0.1)	default value (0.1)	35	default value (False)	default value (None)
6	2.67 µm/pixel	9 days	~ 1.4 mm	0.25	True	default value (0.1)	default value (0.1)	default value (40)	default value (False)	default value (None)

We note that example 1 (marked by an asterisk) is actuated by applying sawtooth pressure waves with ∼ - 6 kPa peak amplitude (equivalent to ∼ 2.5% strain) using a microfluidic pump (Elveflow OB1) to stretch and release the tissue from one side at 0.5 Hz. All other examples are not subjected to any form of actuation. We also note that all masks for this data type were manually generated.

Example_1

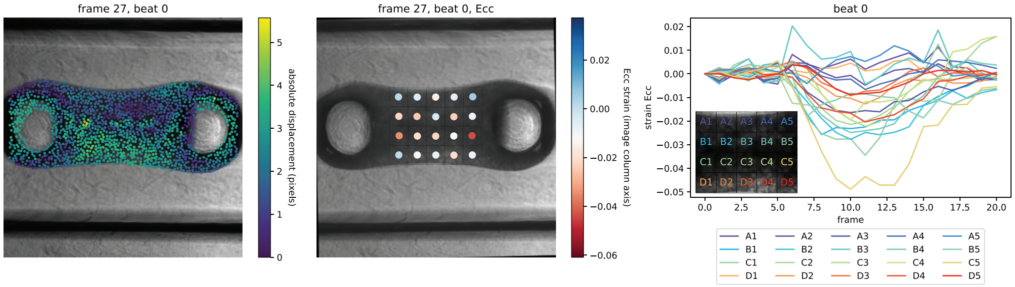

Example_2

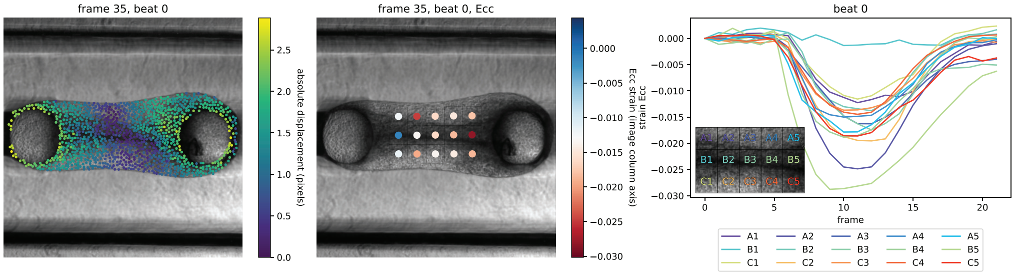

Example_3

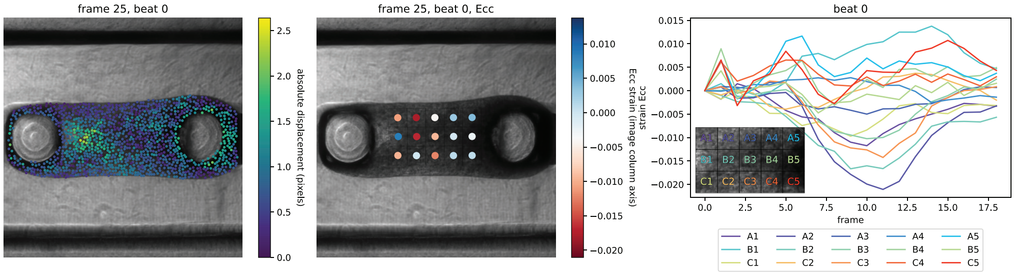

Example_4

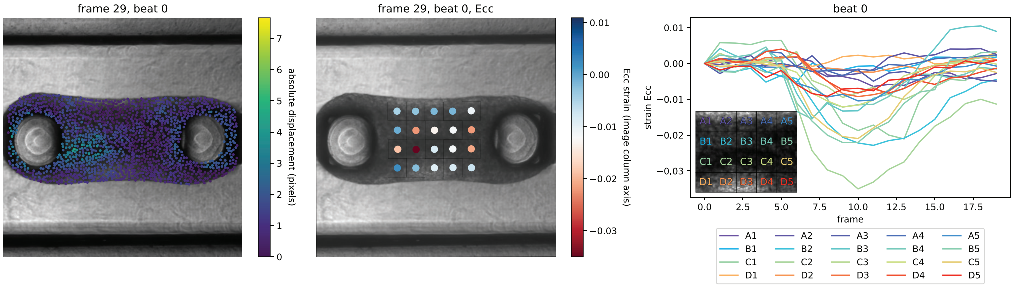

Example_5

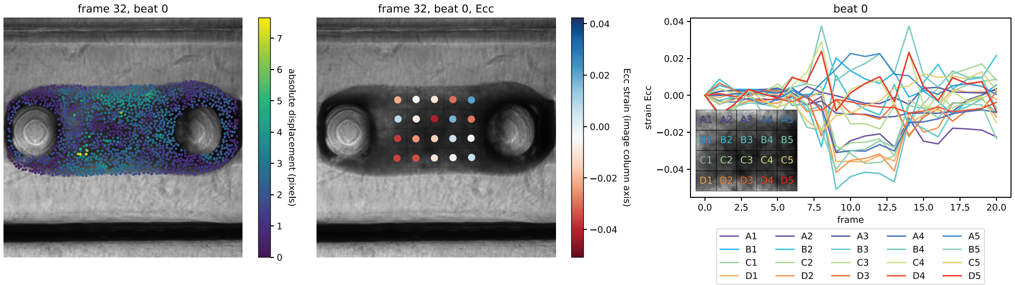

Example_6

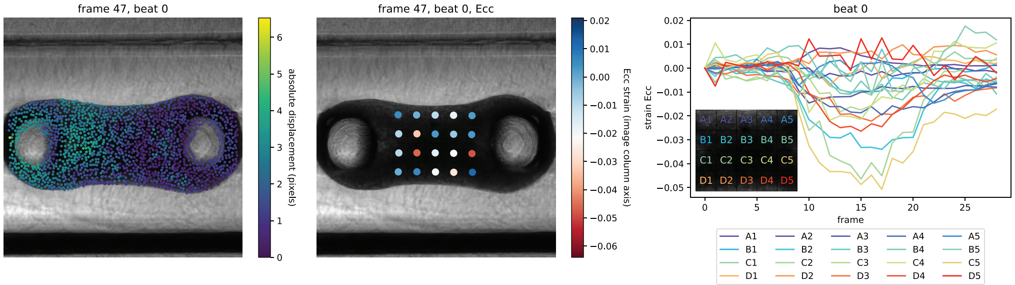

Example_7

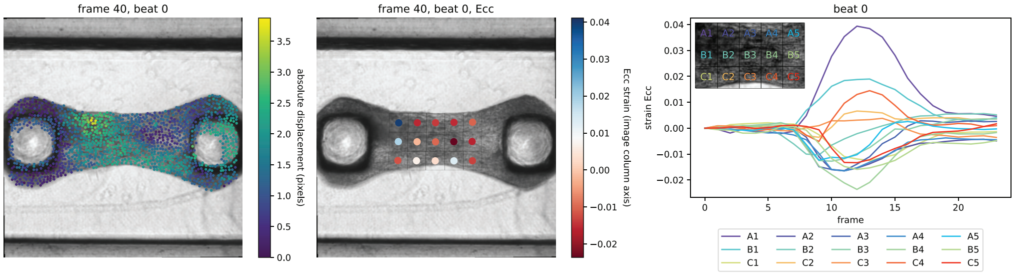

Example_8

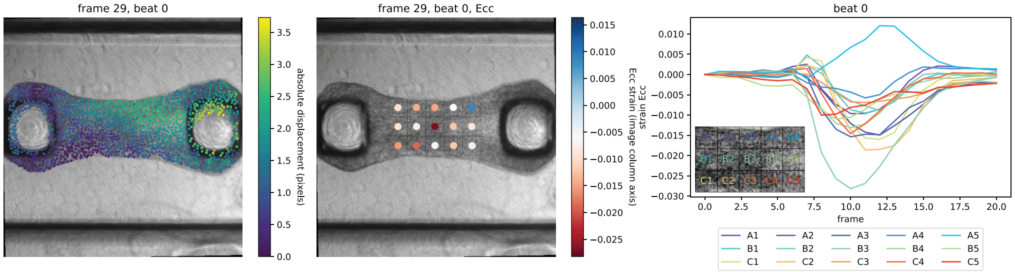

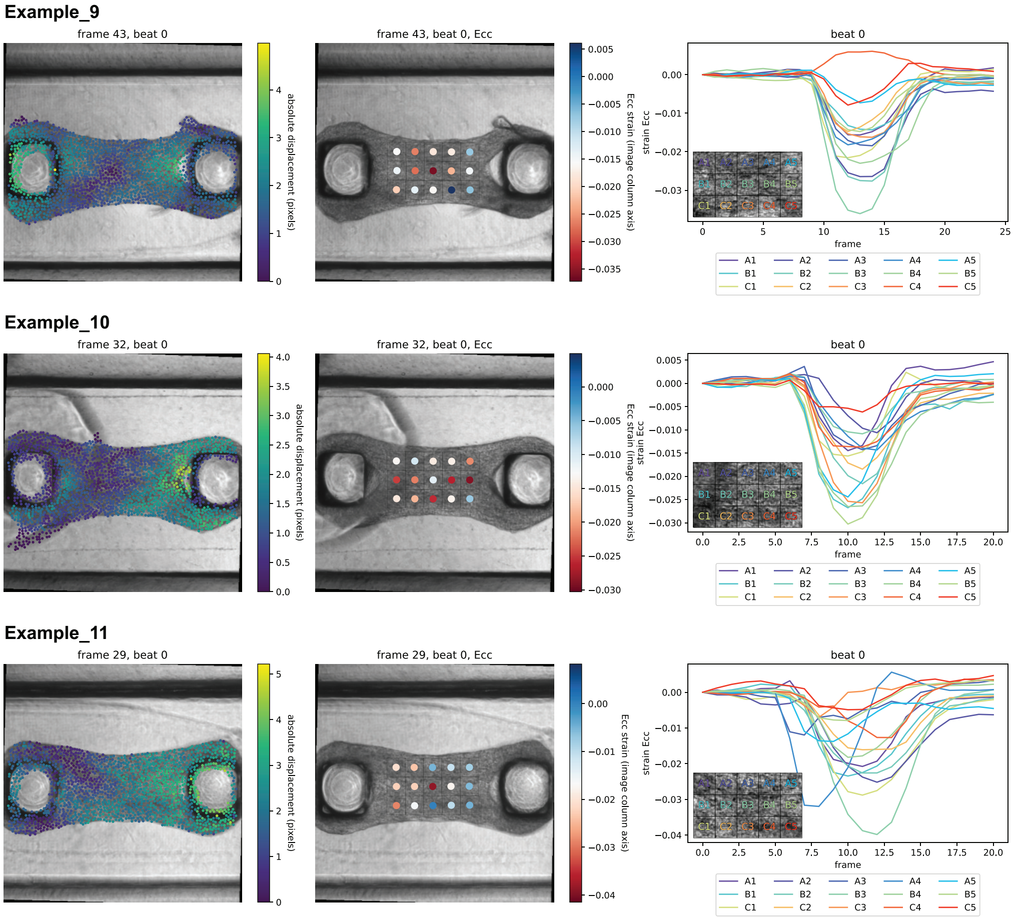

Fig S2_1. Example outputs of "MicroBundleCompute" run on "Type 1" experimental data. Here we show, in order from left to right, full-field mean absolute displacement and subdomain-averaged Green-Lagrange E_{cc} strain at the first tracked peak, as well as a time series plot of E_{cc} strain for the first tracked beat.

Example_1

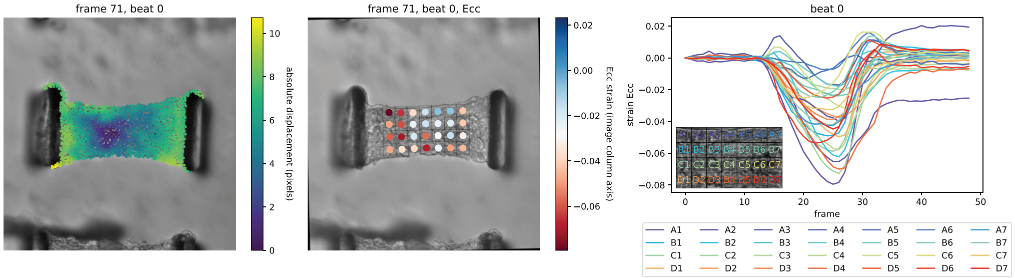

Example_2

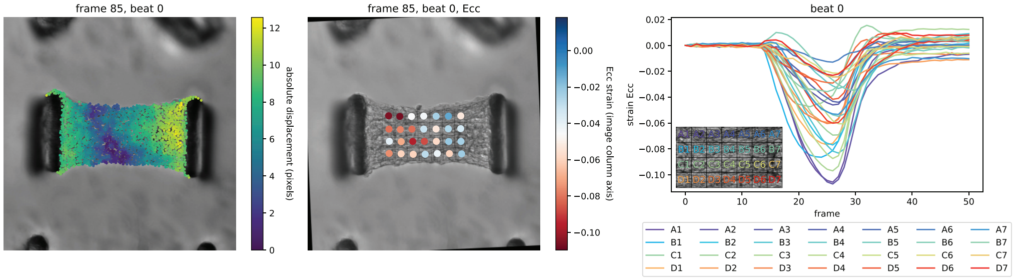

Example_3

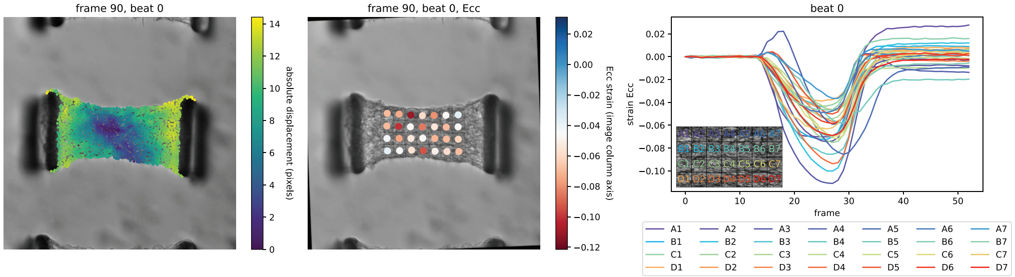

Example_4

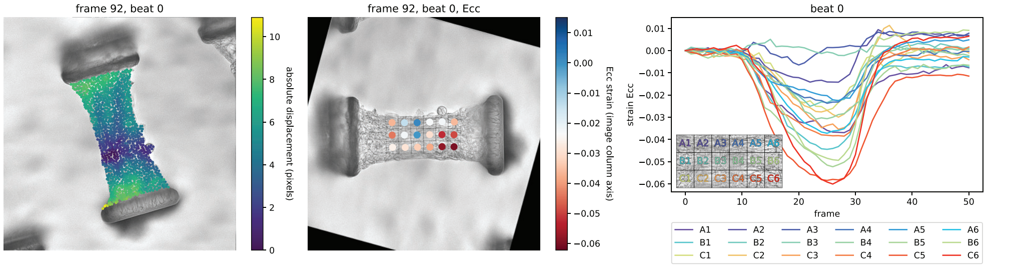

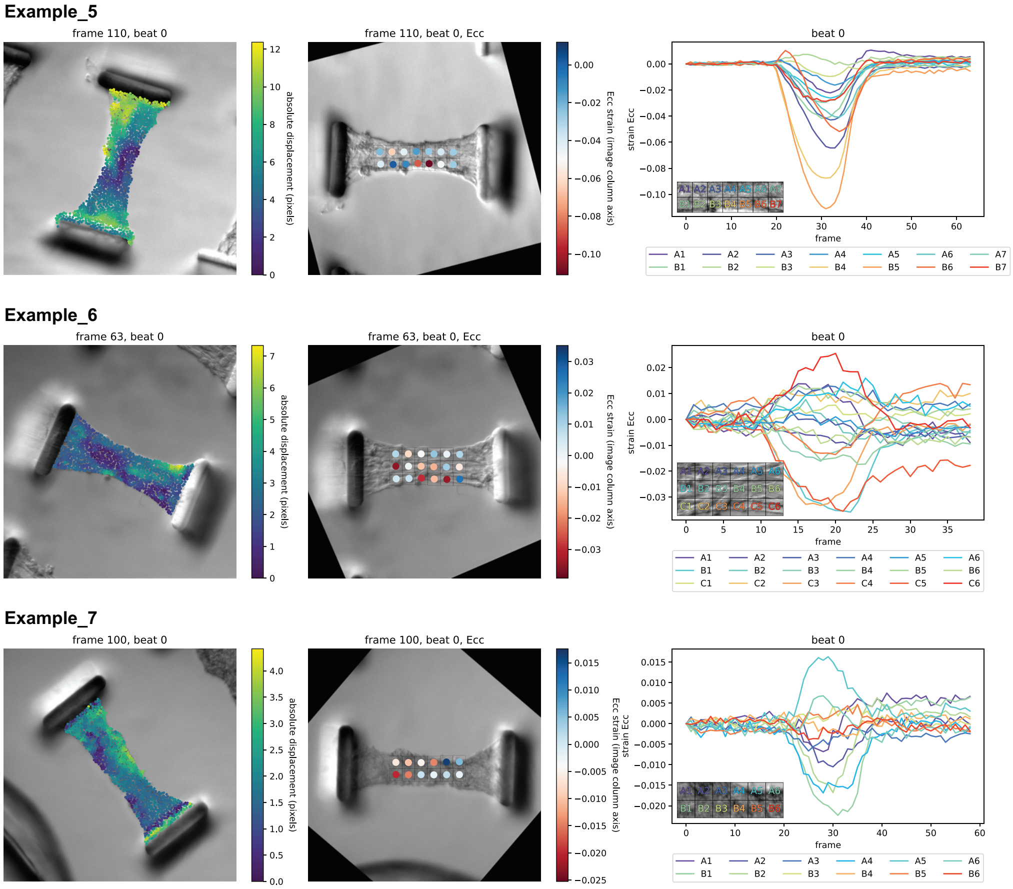

Fig S2_2. Example outputs of "MicroBundleCompute" run on "Type 2" experimental data. Here we show, in order from left to right, full-field mean absolute displacement and subdomain-averaged Green-Lagrange E_{cc} strain at the first tracked peak, as well as a time series plot of E_{cc} strain for the first tracked beat.

Example_1

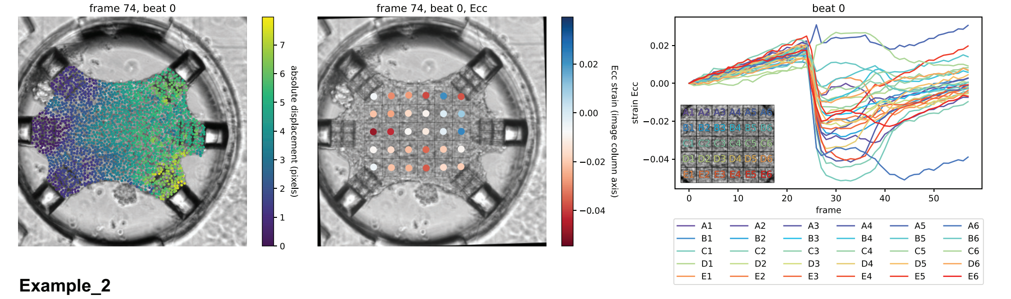

Example_2

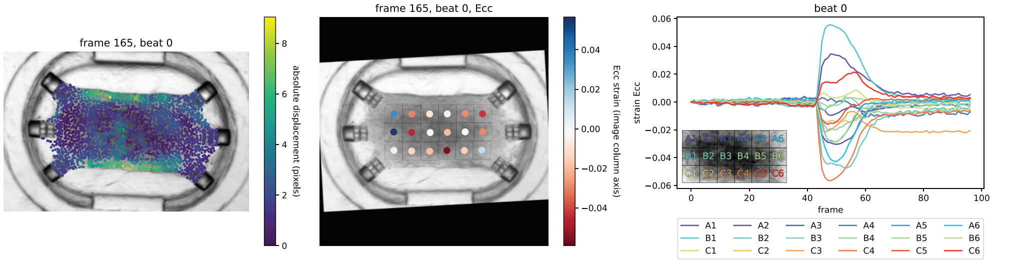

Example_3

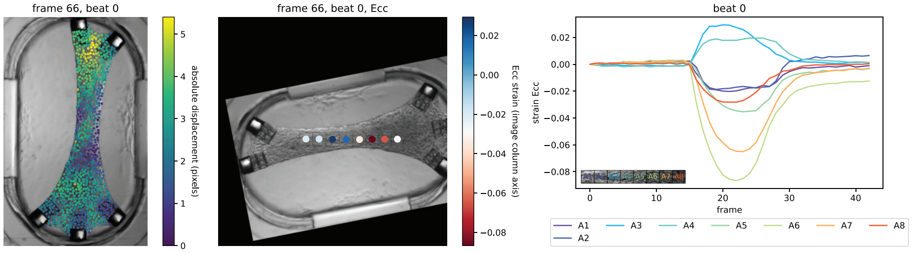

Example_4

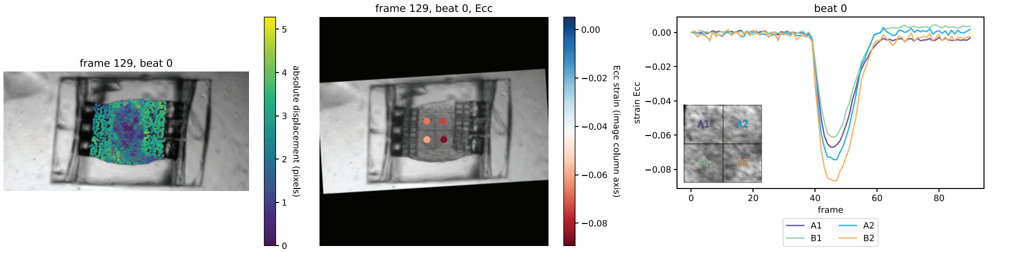

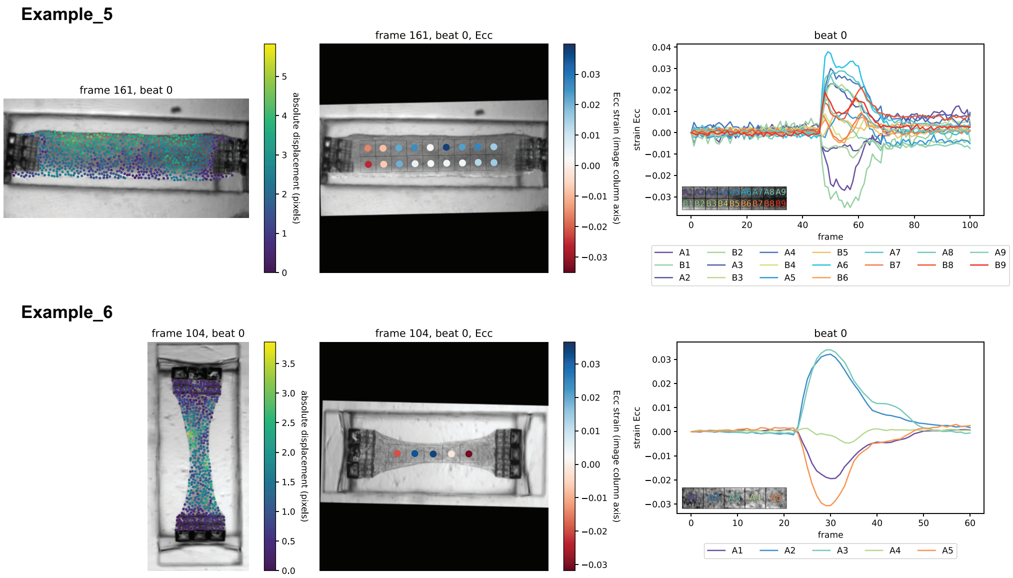

Fig S2_3. Example outputs of "MicroBundleCompute" run on "Type 3" experimental data. Here we show, in order from left to right, full-field mean absolute displacement and subdomain-averaged Green-Lagrange E_{cc} strain at the first tracked peak, as well as a time series plot of E_{cc} strain for the first tracked beat.

References

1. Kobeissi H, Jilberto J, Karakan MÇ, Gao X, DePalma SJ, Das SL, et al.. Microbundle Time-lapse Dataset; Dryad; 2023. doi:https://doi.org/10.5061/dryad.5x69p8d8g.
