# Supplementary material for: MicroBundleCompute: Automated segmentation, tracking, and analysis of subdomain deformation in cardiac microbundles
Source: PLoS One. 2024 Mar 26;19(3):e0298863. doi: 10.1371/journal.pone.0298863 (PMC10965069; doi:10.1371/journal.pone.0298863)
Supplement: S3 Appendix — The implementation of the pillar tracking pipeline within “MicroBundleCompute” is explained in more details and demonstrated on 11 examples of “Type 1” and 7 examples of “Type 2.” Fig S3_1. Pillar tracking on “Type 1” examples. Depicted results include pillar absolute force (μN) obtained on both pillars. Fig S3_2. Pillar tracking on “Type 2” examples. Depicted results include pillar absolute force (μN) obtained on both pillars. (PDF) [file pone.0298863.s003.pdf]

MicroBundleCompute: Automated segmentation, tracking, and analysis of subdomain deformation in cardiac microbundles
S3 Appendix: Pillar tracking

In this Supplementary Document, we elaborate on the basic pillar tracking feature that is included within our computational pipeline. In brief, we demonstrate how the functionality to track tissue deformation can be readily adapted and applied to compute the standard metrics of pillar displacement and subsequently cardiac microbundle twitch force. First, we describe our pipeline to extract pillar displacements, and then we describe our approach to convert these pillar displacements into twitch forces following standard equations from the literature (Section 1) [1]. In Section 2, we show the results obtained from running pillar tracking on our example data. Finally, in Section 3, we briefly describe our future plans to advance our pillar tracking functionality beyond the scope of this project.

1 Methods

Here we describe our straightforward approach to tracking microbundle pillars and outputting twitch forces. Briefly, the pillar tracking functionality requires either manually or externally generating separate masks of each pillar, and follows a similar pipeline to the microbundle tissue tracking described in the "Code" Section of the main paper document with one main difference: temporal segmentation is by default skipped. Instead, we directly compute the mean position of all tracked points at every time step, and subsequently derive the mean absolute displacement relative to the first frame, considered to be a valley frame. However, we retain the temporal segmentation as an optional step to remove any drift present in the tracked results, as explained in the "Temporal segmentation" Section of the main paper document. We briefly note that this approach is possible because we do not output full-field pillar displacement results.

Following standard approaches in the literature [1], the pillar directional and absolute forces can be computed from the obtained mean directional and absolute displacement or deflection results. Specifically, an approximation of the pillar force F can be found by applying Hooke's law:

$$F = k\delta \tag{1}$$

where the deflection δ refers to the mean tracked pillar displacement and the combined geometric and material pillar stiffness k is provided as an experimentally derived quantity.

Alternatively, if experimental testing data is not available, the poly(dimethylsiloxane) (PDMS) molded pillars can be approximated as cantilever beams [1, 2], and the stiffness k can be determined by the pillar geometry and material properties as:

$$k = \frac{6EI}{a^2(3L - a)} \tag{2}$$

where E is the material's elastic modulus, a is the location of force application, L is the cantilever length, and finally I is the moment of inertia defined as a function of the cantilever's geometry. For rectangular cross section beams, $I = \frac{wt^3}{12}$ where w is the pillar width and t is the pillar thickness while for circular cross section beams, $I = \frac{\pi D^4}{64}$

where D is the cylindrical pillar diameter. To compute mean tissue stress, pillar force is divided by tissue cross sectional area, where the latter area is a function of tissue width computed from the tissue mask geometry, and experimentally measured tissue depth.

Experimental platforms that are based on pillar constructs (i.e., the "Type 1" and "Type 2" data introduced prior) are amenable to pillar tracking. We share our example implementation as well as general guidelines to use this feature on the "MicroBundleCompute" GitHub page (https://github.com/HibaKob/MicroBundleCompute). For our examples of "Type 1" data, we run the code with a pillar stiffness of $k = 2.677\mu\text{N}/\mu\text{m}$ and length scale (ls) $4\mu\text{m/pixel}$. For the "Type 2" examples, we specify $k = 0.41\mu\text{N}/\mu\text{m}$ and $ls = 0.908\mu\text{m/pixel}$.

2 Results

Here, we present the results of implementing the pillar tracking pipeline with all 14 experimental examples of "Type 1" and "Type 2" data. Results are shown in Figs S3_1 and S3_2 and reveal that the absolute force is not always consistent among the two pillars. While imaging artifacts, present in both types of data, might marginally contribute to the discrepancy in measured force outputs, we believe that there are a number of mechanistic reasons that we have yet to investigate, as we explain in Section 3. Of note, "Type 1" data was run without temporal segmentation while "Type 2" data displayed non-trivial drift and required the temporal segmentation step. We attribute this difference to the out-of-plane motion that is more significantly visible in the substantially thinner "Type 2" tissues.

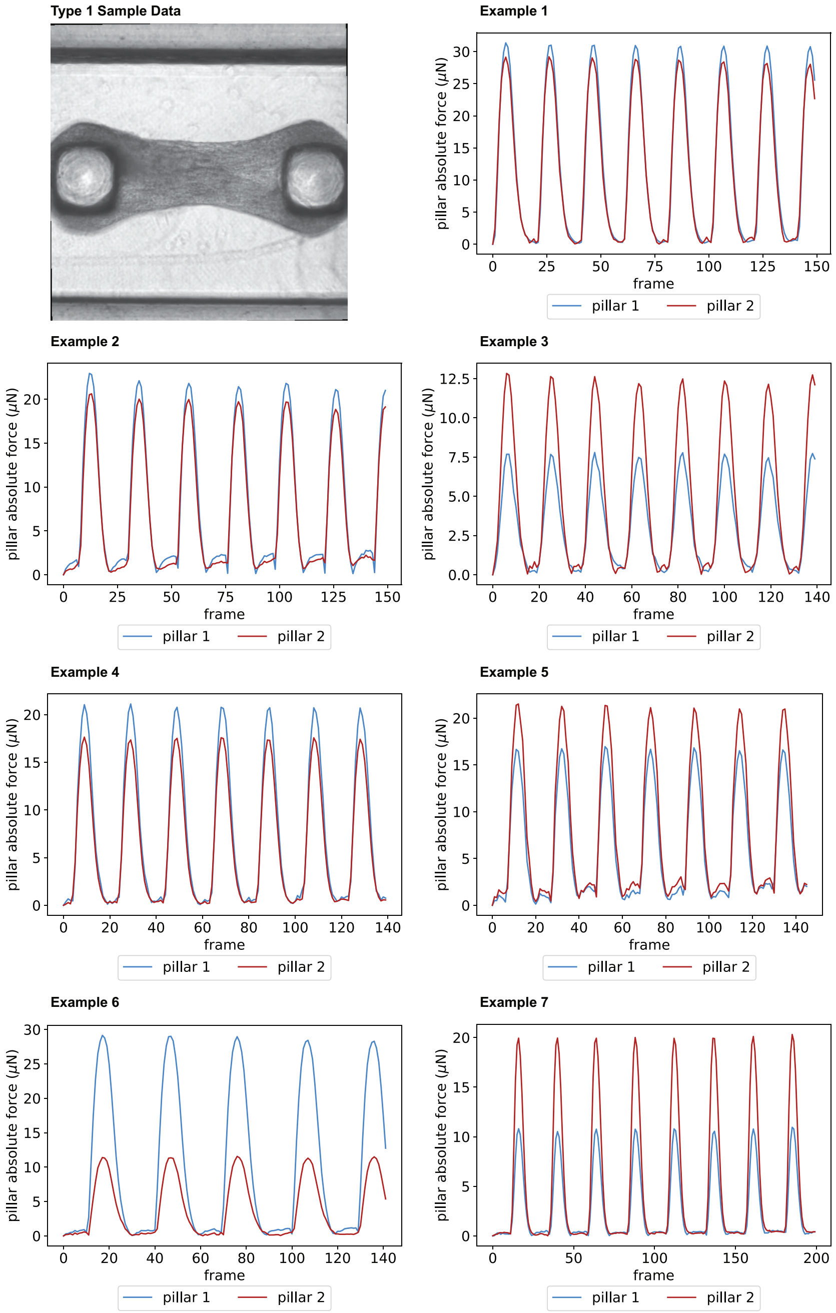
Type 1 Sample Data
Example 1
pillar absolute force (μN)
frame
pillar 1
pillar 2
Example 2
pillar absolute force (μN)
frame
pillar 1
pillar 2
Example 3
pillar absolute force (μN)
frame
pillar 1
pillar 2
Example 4
pillar absolute force (μN)
frame
pillar 1
pillar 2
Example 5
pillar absolute force (μN)
frame
pillar 1
pillar 2
Example 6
pillar absolute force (μN)
frame
pillar 1
pillar 2
Example 7
pillar absolute force (μN)
frame
pillar 1
pillar 2

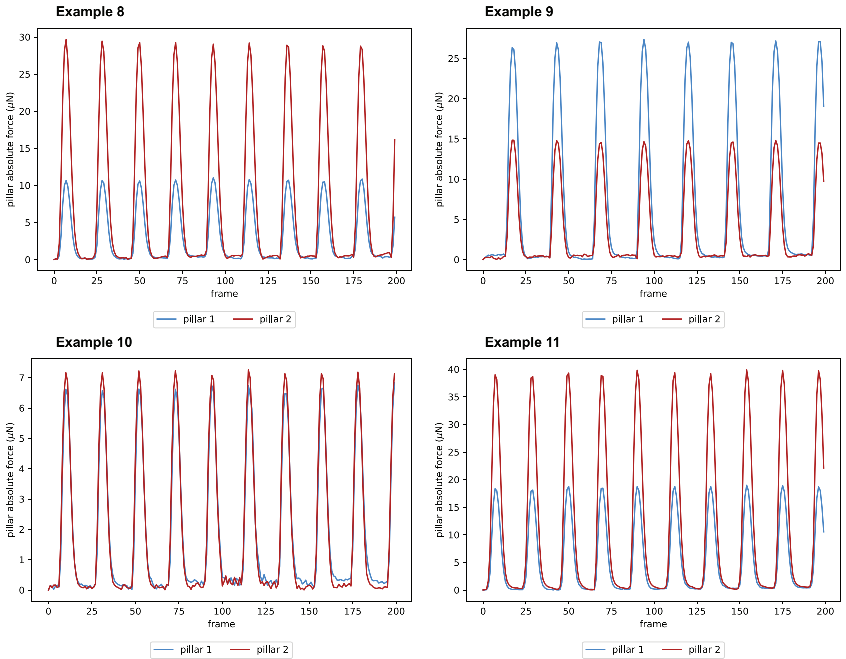

Fig S3_1. Pillar absolute force (μN) obtained by running the pillar tracking pipeline with "Type 1" data. We note that, in these cases,"pillar 1" and "pillar 2" in the legends consistently refer to the left and right pillars, respectively. However, in general, "pillar 1" would refer to the user-defined pillar mask saved as "pillar_mask_1.txt" and "pillar 2" to the pillar whose mask is defined by "pillar_mask_2.txt".

3 Future Work

In this document, we have provided a description of the pillar tracking functionality included within the "MicroBundleCompute" computational framework and have shared representative examples of implementing this functionality. We believe that, in its current state, the pillar tracking option would be useful to a number of researchers, given its ease of use. However, we have multiple plans to further improve our pipeline including:

- automatic pillar mask extraction
- validated outputs against synthetic data and against other tools currently available in the literature [3]
- generalized pillar tracking functionality that can be implemented on experimental setups beyond the scope of the "Type 1" and "Type 2" data examples shown here

In addition, by examining the time series plots of the pillar absolute force for both pillars in Figs S3_1 and S3_2, we can see that the deflections of two pillars contained within one experimental sample do not always match. There are multiple potential mechanistic explanations of this observation. For example, the tissue may be at different vertical positions on each pillar, pillars may have variable material or geometric properties, or there may be an asymmetry in tissue-pillar interaction that we do not currently understand. Further investigations are needed to both formulate solid

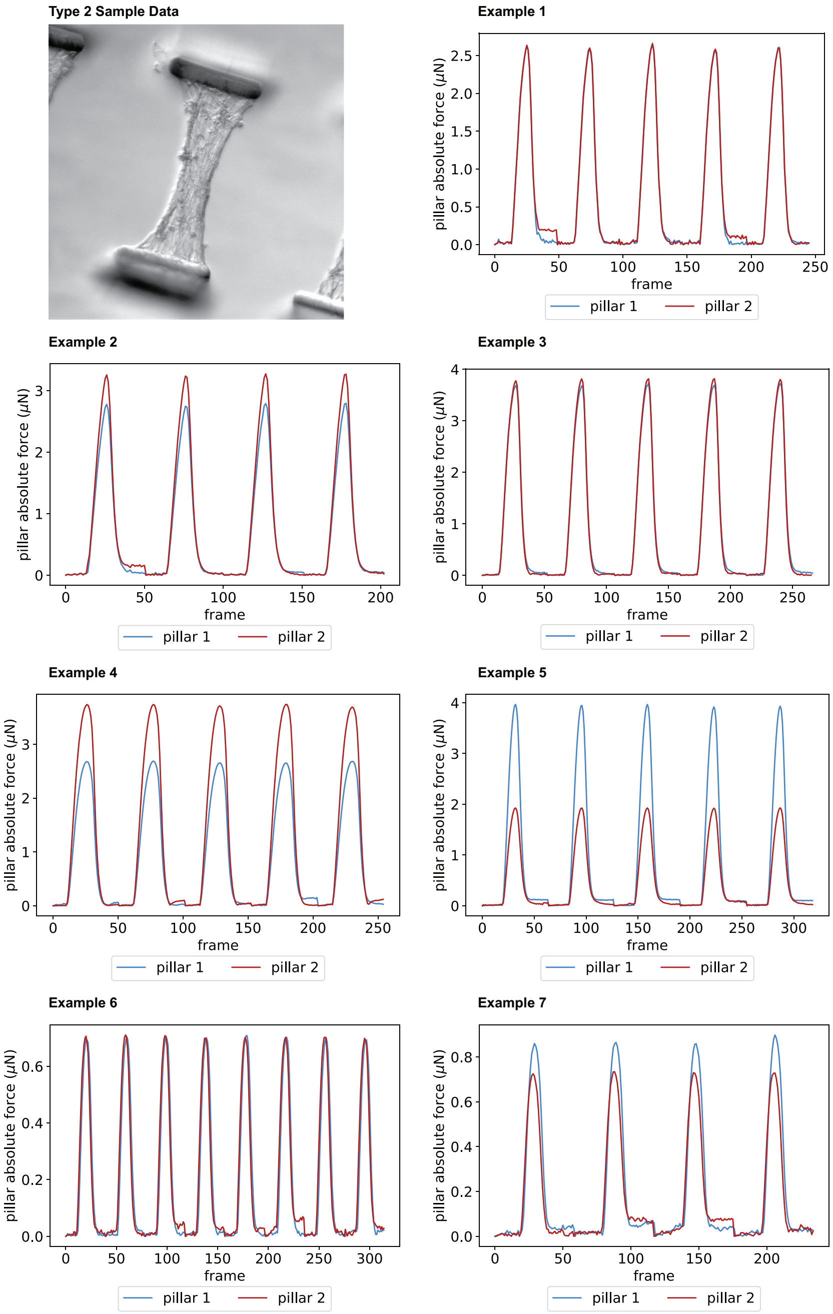

Fig S3_2. Pillar absolute force (μN) obtained by running the pillar tracking pipeline with "Type 2" data. We note that, in these cases, "pillar 1" refers to the user-defined pillar mask saved as "pillar_mask_1.txt" and "pillar 2" to the pillar whose mask is defined by "pillar_mask_2.txt".

explanations of this finding, and develop the appropriate statistical tools to address the associated uncertainty as a companion to our software.

References

1. Legant WR, Pathak A, Yang MT, Deshpande VS, McMeeking RM, Chen CS. Microfabricated tissue gauges to measure and manipulate forces from 3D microtissues. Proceedings of the National Academy of Sciences. 2009;106(25):10097–10102. doi:https://doi.org/10.1073/pnas.0900174106.

2. Das SL, Sutherland BP, Lejeune E, Eyckmans J, Chen CS. Mechanical response of cardiac microtissues to acute localized injury. American Journal of Physiology-Heart and Circulatory Physiology. 2022;323(4):H738–H748. doi:https://doi.org/10.1152/ajpheart.00305.2022.

3. Tamargo MA, Nash TR, Fleischer S, Kim Y, Vila OF, Yeager K, et al. milliPillar: a platform for the generation and real-time assessment of human engineered cardiac tissues. ACS biomaterials science & engineering. 2021;7(11):5215–5229. doi:https://doi.org/10.1021/acsbiomaterials.1c01006.
