# Supplementary figures and images for: MicroBundleCompute: Automated segmentation, tracking, and analysis of subdomain deformation in cardiac microbundles
Source: PLoS One. 2024 Mar 26;19(3):e0298863. doi: 10.1371/journal.pone.0298863 (PMC10965069; doi:10.1371/journal.pone.0298863)

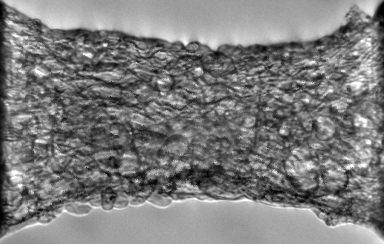

Supplement: S1 Movie — This movie provides the synthetic example generated based on “Type 2” data as described in S1 Appendix. (TIF) [file pone.0298863.s004.tif]
