# Supplementary material for: MicroBundleCompute: Automated segmentation, tracking, and analysis of subdomain deformation in cardiac microbundles
Source: PLoS One. 2024 Mar 26;19(3):e0298863. doi: 10.1371/journal.pone.0298863 (PMC10965069; doi:10.1371/journal.pone.0298863)
Supplement: Attachment — Submitted filename: Response to Reviewers.pdf [file pone.0298863.s017.pdf]

MicroBundleCompute: Automated segmentation, tracking, and analysis of subdomain deformation in cardiac microbundles - [PONE-D-23-28138]
Response to Reviewers

We would like to begin by thanking the Editor and the Reviewers for taking the time to evaluate our submission. We sincerely appreciate their detailed feedback and their consideration of the work. In response to this feedback, we have made several clarifying changes to the text and performed additional analyses. Specifically, we supplemented our pipeline validation studies with testing "MicroBundleCompute" on synthetic data with added shot noise. We have also added strain validation for the more complex "Type 2" synthetic example data to make the analysis more comprehensive. Finally, we compared the performance of "MicroBundleCompute" to the 2 "less automated" methods that we had cited in our manuscript. We include the findings of these additional analyses either as responses within this document or as updates to the main manuscript and accompanying supplementary documents. We note that changes made to the text in response to reviewer feedback are highlighted in red in the revised submission. We believe that these changes and clarifications are sufficient to address all of the reviewer's concerns.

Academic Editor Comments and Response

1. Please ensure that your manuscript meets PLOS ONE's style requirements, including those for file naming. The PLOS ONE style templates can be found at https://journals.plos.org/plosone/s/file?id=wjVg/PLOSOne_formatting_sample_main_body.pdf and https://journals.plos.org/plosone/s/file?id=ba62/PLOSOne_formatting_sample_title_authors_affiliations.pdf

- Thank you for sharing the relevant links. We have now updated to match the style guide.

2. Please update your submission to use the PLOS LaTeX template. The template and more information on our requirements for LaTeX submissions can be found at http://journals.plos.org/plosone/s/latex.

- Thank you for pointing this out. We now use the PLOS LaTeX template.

3. We note that the grant information you provided in the "Funding Information" and "Financial Disclosure" Sections do not match.

When you resubmit, please ensure that you provide the correct grant numbers for the awards you received for your study in the "Funding Information" Section.

- Thank you for pointing this out. It is now corrected.

4. Thank you for stating the following financial disclosure: [This work was supported by the CELL-MET Engineering Research Center National Science Foundation ECC-1647837. See:https://www.bu.edu/cell-met/ andhttps://nsf.gov/awardsearch/showAward?AWD_ID=1647837]. Please state what role the funders took in the study. If the funders had no role, please state: ""The funders had no role in study design, data collection and analysis, decision to publish, or preparation of the manuscript."" If this statement is not correct you must amend it as needed. Please include this amended Role of Funder statement in your cover letter; we will change the online submission form on your behalf.

When you resubmit, please ensure that you provide the correct grant numbers for the awards you received for your study in the "Funding Information" Section.

- Thank you for pointing this out. It is now updated.

5. We note that Figure 1, 2, 3, 4A, 5, 6, 7, S1_1, S1_7, S1_8, S1_9, S1_10, S1_11, S1_12, S1_13, S1_14, S1_15, S1_16, S1_17, S1_18, S1_19, S1_20, S1_21, S1_22, S1_25, S1_26, S2_1, S2_2, S2_3, S3_1, S3_2 and S1 to S13. in your submission contain copyrighted images. All PLOS content is published under the Creative Commons Attribution License (CC BY 4.0), which means that the manuscript, images, and Supporting Information files will be freely available online, and any third party is permitted to access, download, copy, distribute, and use these materials in any way, even commercially, with proper attribution. For more information, see our copyright guidelines: http://journals.plos.org/plosone/s/licenses-and-copyright.

We require you to either (1) present written permission from the copyright holder to publish these figures specifically under the CC BY 4.0 license, or (2) remove the figures from your submission:

A. You may seek permission from the original copyright holder of Figure 1, 2, 3, 4A, 5, 6, 7, S1_1, S1_7, S1_8, S1_9, S1_10, S1_11, S1_12, S1_13, S1_14, S1_15, S1_16, S1_17, S1_18, S1_19, S1_20, S1_21, S1_22, S1_25, S1_26, S2_1, S2_2, S2_3, S3_1, S3_2 and S1 to S13 to publish the content specifically under the CC BY 4.0 license.

We recommend that you contact the original copyright holder with the Content Permission Form (`http://journals.plos.org/plosone/s/file?id=7c09/content-permission-form.pdf`) and the following text: "I request permission for the open-access journal PLOS ONE to publish XXX under the Creative Commons Attribution License (CCAL) CC BY 4.0 (`http://creativecommons.org/licenses/by/4.0/`). Please be aware that this license allows unrestricted use and distribution, even commercially, by third parties. Please reply and provide explicit written permission to publish XXX under a CC BY license and complete the attached form."

Please upload the completed Content Permission Form or other proof of granted permissions as an ""Other"" file with your submission.

In the figure caption of the copyrighted figure, please include the following text: "Reprinted from [ref] under a CC BY license, with permission from [name of publisher], original copyright [original copyright year]."

B. If you are unable to obtain permission from the original copyright holder to publish these figures under the CC BY 4.0 license or if the copyright holder's requirements are incompatible with the CC BY 4.0 license, please either i) remove the figure or ii) supply a replacement figure that complies with the CC BY 4.0 license. Please check copyright information on all replacement figures and update the figure caption with source information. If applicable, please specify in the figure caption text when a figure is similar but not identical to the original image and is therefore for illustrative purposes only.

- Thank you for pointing this out. To clarify, all elements of the abovementioned figures were generated by the authors of this paper. Critically, these data were uploaded to the Open Access Dryad digital repository (`https://doi.org/10.5061/dryad.5x69p8d8g`) with CC0 1.0 Licenses for the images, which means that anyone can "copy, modify, distribute, and perform the work, even for commercial purposes, all without asking permission." Therefore, we automatically have permission to re-use and publish these data.

6. Please include captions for your Supporting Information files at the end of your manuscript, and update any in-text citations to match accordingly. Please see our Supporting Information guidelines for more information: `http://journals.plos.org/plosone/s/supporting-information`.

- Thank you for pointing out that this was missing. This is fixed in the revised manuscript.

7. We notice that your supplementary [figures/tables] are included in the manuscript file. Please remove them and upload them with the file type 'Supporting Information'. Please ensure that each Supporting Information file has a legend listed in the manuscript after the references list.

- Thank you for pointing out that this was missing. This is fixed in the revised manuscript.

8. We are unable to open your Supporting Information file [_MACOSX]. Please kindly revise as necessary and re-upload.

- Thank you for pointing this out. This file is an artifact, and if it remains in the new submission it can be ignored.

Referee 1 Comments and Response

This work describes an open-source Python tool, MicroBundleCompute that computationally tracks the deformation from movie data of in vitro hiPSC-CM and predicts the physical properties such as strain. The tool is validated on synthetic data from FEA simulations mimicing the experimental setup. It is also demonstrated on various experimental data. The manuscript is well written with detailed explanations and many examples. The software is also well implemented with clear tutorials. While the methodological novelty is rather limited, I believe this work provides a versatile and reliable computational tool for high-throughput evaluation of hiPSC-CM experimental data. I only have some minor comments.

1. It would be helpful to add validation results on synthetic data for type 2 and type 3 as well as done for type 1 in Figure 4.

- Thank you for this comment – we apologize that it was not clearer in the manuscript that we have performed additional validation on "Type 2" data reported in S1 Appendix. Specifically, we have performed less extensive validation on "Type 2" data including both synthetic and manually tracked examples. We have updated the revised submission to clarify this and also added additional validation for strain outputs to make this analysis more comprehensive. The additional study of strain is added in S1 Appendix, Fig S1_26. For the "Type 3" data, we do not include separate validation studies because the tissue preparation methods are very similar to the ones used for "Type 1" data which results in similar tissue textures. In addition, the "Type 3" data platform geometry is versatile enough that there is not necessarily a representative boundary condition (for example, "Type 3" Examples 4 and 5 exhibit similar behavior to "Type 1" data). We hope the addition of more "Type 2" validation addresses this concern.

2. Two other "less automated" methods were mentioned in Introduction, "Huebsch, Nathaniel, et al. "Automated video-based analysis of contractility and calcium flux in human-induced pluripotent stem cell-derived cardiomyocytes cultured over different spatial scales." Tissue Engineering Part C: Methods 21.5 (2015): 467-479." and "Méry, Adrien, et al. "Light-driven biological actuators to probe the rheology of 3D microtissues." Nature Communications 14.1 (2023): 717." Some comparison should be performed with these methods on both synthetic data and experimental data.

- Thank you to the reviewer for this thoughtful suggestion. As a brief note, ***we intend to publish the "Review" and our "Response to Reviewers" document alongside this manuscript.*** Here, in our "Response to Reviewers" document, we will share the outputs of using both software on 2 synthetic examples, one featuring *super*-pixel displacements, ST_1, and one with entirely *sub*-pixel displacements, ST_3, and highlight the main aspects of our experience when implementing them and how they compare to our software, "MicroBundleCompute."

  First, we note that both of these alternative software are designed to give contraction rate outputs (i.e. contraction velocity, strain rate, etc.), while "MicroBundleCompute" outputs displacements and displacement-derived metrics. To be able to compare between the 3 tools, we set the frame rate or time step as 1 frame per unit time for "Huebsch, Nathaniel, et al." Motion_Tracking software and PIVlab used in "Méry, Adrien, et al." to obtain what we believe represents the displacement per a single frame without further post-processing the results and avoiding the introduction of additional errors into the process. The results of exploring these alternative options are as follows:

1. **"Huebsch, Nathaniel, et al.": Motion_Tracking**

   For the software developed by Huebsch et al., we first note that it was not clear to us if there is a recommended version of MATLAB for running the software (we found only a tutorial provided as a supplementary document). For reference, we ran the tests using MATLAB R_2023a which was the version installed on our machines. As a brief note, the graphical user interface (GUI) had some minor differences as compared to the screenshot included within the software tutorial. We provide a screenshot of the interface along with the parameters we used in Fig 1 below. We also faced minor difficulties in loading the movie frames into the software, where we received the error shown in Fig 2 multiple times. Our workaround was to click on "Reset all" after selecting the desired folder and then selecting the folder again.

   Although we checked the box in the GUI to indicate "Displacement/Strain" as measurement output, we could only obtain contraction rate results as shown in Figs 3 and 4 (with ST_1 as the representative example). Nonetheless, following the approach mentioned above (frame 1 as the reference frame, one frame per unit time), we can treat these outputs as displacements per single frame results.

   In Fig 5, we compare the mean absolute "displacement" output of the "Huebsch, Nathaniel, et al." motion tracking software to the known ground truth for synthetic examples ST_1 and ST_3, respectively. At peak displacements, the errors fall below 8.9% for ST_1 and 77.3% for ST_3 (for reference, these errors compare to 2.5% and 9.6% respectively with "MicroBundleCompute"). Computed R^2 values show higher agreement between tracked and ground truth mean absolute displacement for ST_1 with a minimum value of 0.984 compared to example ST_3 where R^2 values fall below 0.424 (for reference, these values compare to 0.998 and 0.990 respectively with "MicroBundleCompute").

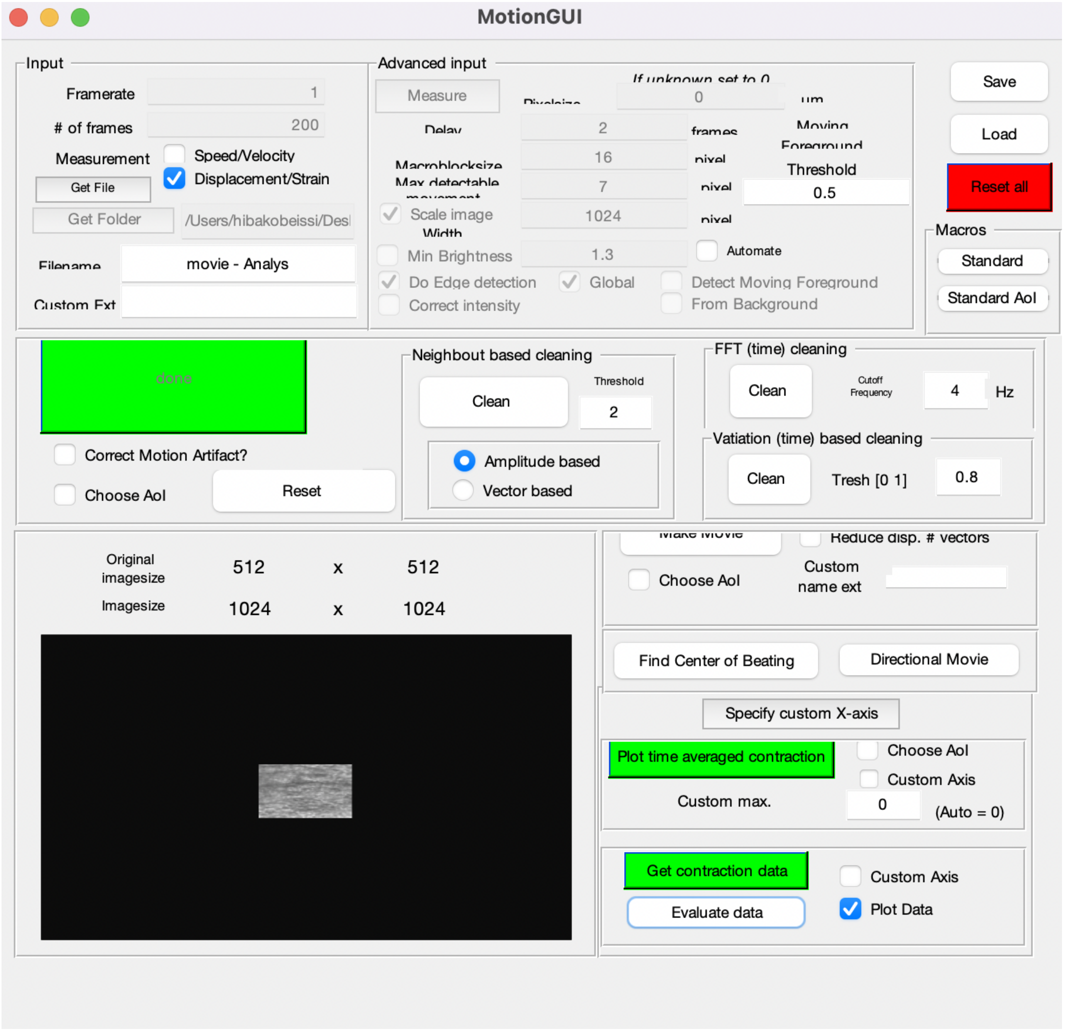

Figure 1: Screenshot of the motion tracking GUI developed by “Huebsch, Nathaniel, et al.”

```
Dot indexing is not supported for variables of this type.

Error in MotionGUI>GETFOLDER_Callback (line 105)
    ScaleWidth = str2double(get(handles.ScaleWidth,'String'));

Error in gui_mainfcn (line 95)
        feval(varargin{:});

Error in MotionGUI (line 20)
    gui_mainfcn(gui_State, varargin{:});

Error in matlab.graphics.internal.figfile.FigFile/read>@(hObject,eventdata)MotionGUI('GETFOLDER_Callback',

Error while evaluating UIControl Callback.
```

Figure 2: Screenshot of the obtained error when loading the movie folder using the software from “Huebsch, Nathaniel, et al.”

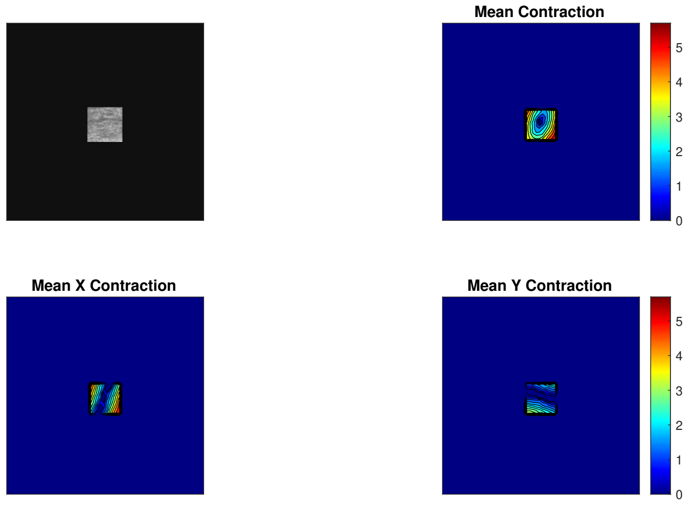

Figure 3: Screenshot of the obtained mean contraction outputs from "Huebsch, Nathaniel, et al." for example ST_1.

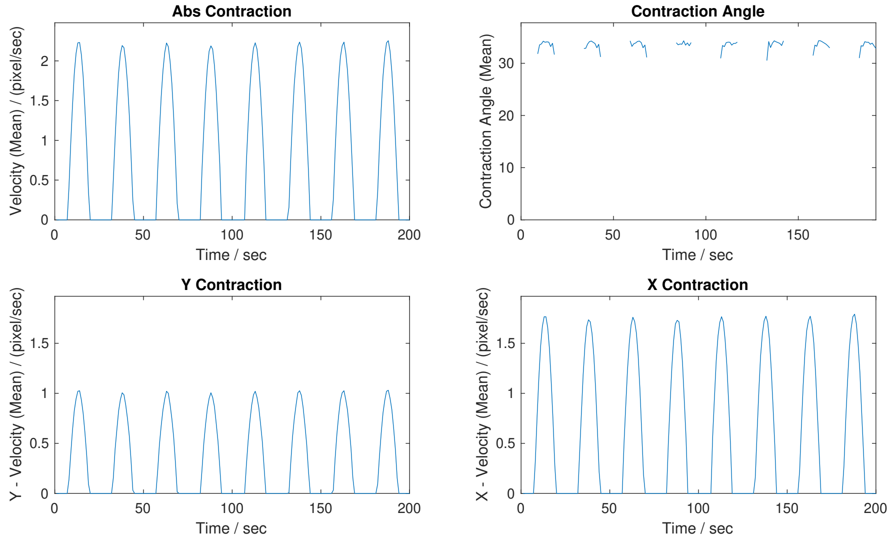

Figure 4: Screenshot of the obtained velocity outputs from "Huebsch, Nathaniel, et al." for example ST_1.

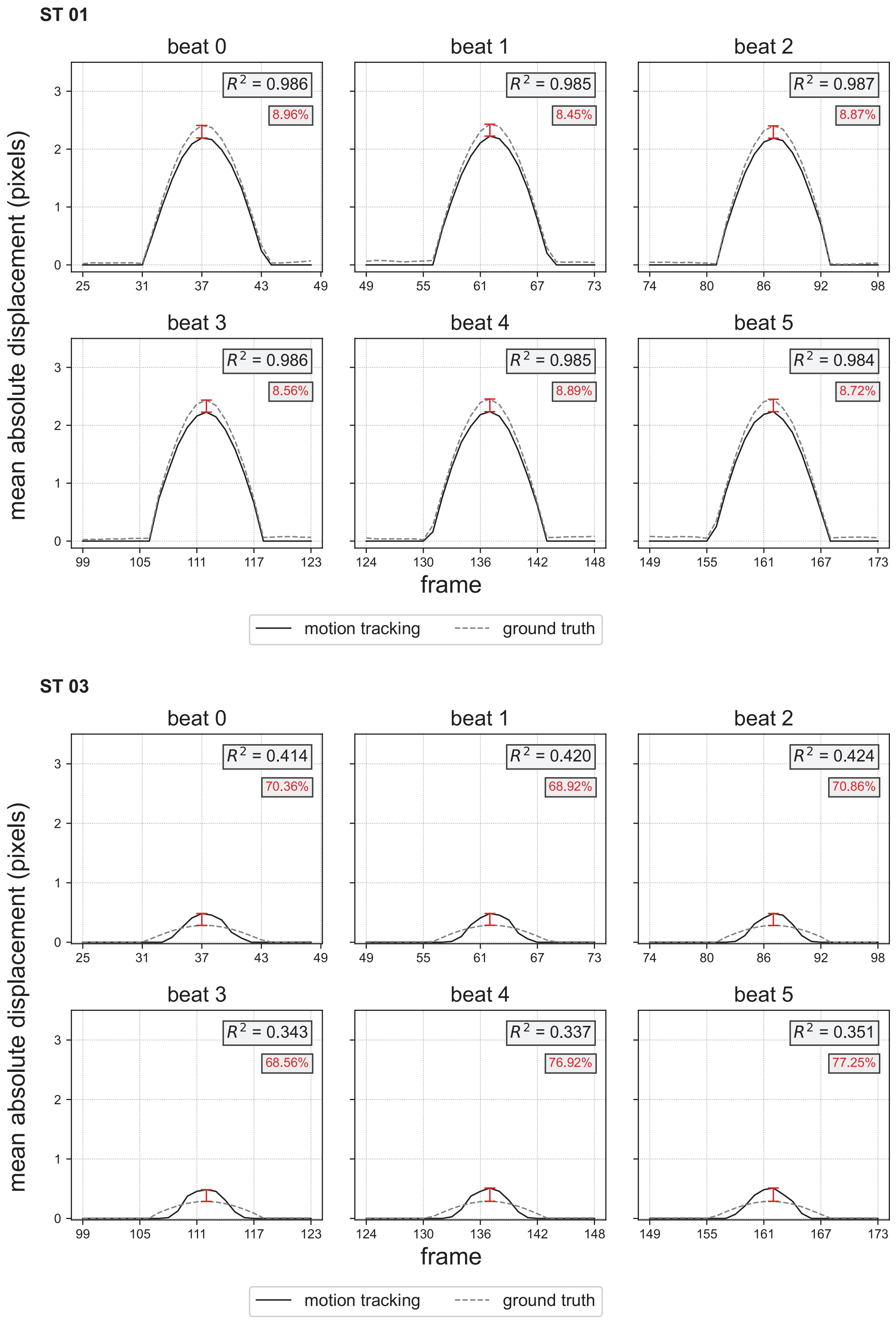

Figure 5: Errors on mean absolute displacement for synthetic examples ST_1 and ST_3 analyzed with the motion tracking software developed by Huebsch, Nathaniel, et al.

2. **“Méry, Adrien, et al.”: PIVlab**

   Following the same approach and assumptions, we extract mean absolute displacement results by running PIVlab with synthetic examples ST_1 and ST_3.

   In Fig 6, we show that at peak displacements, the errors fall below 11.2% for ST_1 and 13.8% for ST_3 (for reference, these errors compare to 2.5% and 9.6% respectively with “MicroBundleCompute”). Computed R^2 values show good agreement between tracked and ground truth mean absolute displacement for both ST_1 and ST_3 with minimum values of 0.977 and 0.962, respectively (for reference, these values compare to 0.998 and 0.975 respectively with “MicroBundleCompute”).

In brief, both of these software are designed to measure velocity. Through setup parameter selection, we are able to obtain displacements from both of these software as a point of comparison with “MicroBundleCompute.” However, we stop short of performing a similar comparison for strain outputs for the following reasons: 1) it was not possible to obtain strains with the “Huebsch, Nathaniel, et al.” Motion_Tracking software (the program did not respond to the “Displacement/Strain” box checked on the GUI), 2) for the PIVlab software, we were not entirely confident that their definitions of output strain would match our approach (i.e., simple strain rate, and simple shear rate), and 3) to perform a direct comparison, we would have to calculate the subdomain-averaged strains which might introduce additional errors into the comparisons pipeline. Based on these factors, we do not feel confident including these comparisons as part of the revised manuscript or the supplementary documents. Therefore (in addition to referencing these software as examples of alternative approaches) we have decided to restrict our direct comparison to the “Response to the Reviewers” document which we intend to publish alongside the manuscript.

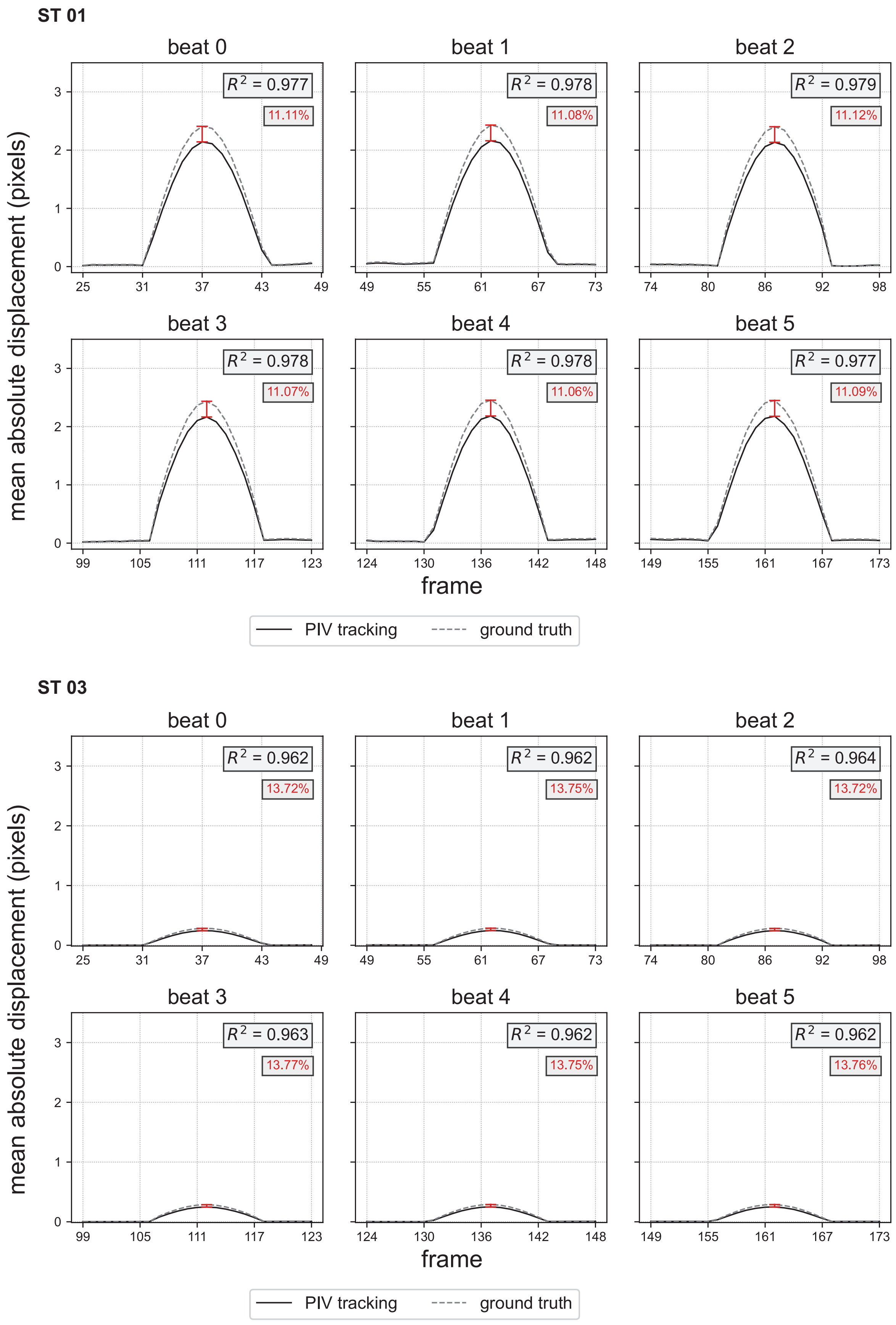

Figure 6: Errors on mean absolute displacement for synthetic examples ST_1 and ST_3 analyzed with PIVlab.

Referee 2 Comments and Response

The authors present a method for quantifying the contraction of cardiomyocyte microbundles, a problem which is currently very topical, with the recent rise of engineered heart tissue studies. The authors further present a method for generating simulated videos of contracting microbundles with solid modelling background. The paper is well written and the methods thoroughly documented. With the software released as modular and open source software, it is my belief that the paper and software will prove to be very useful for researchers working in the field. The language is impeccable.

In general, I only have few remarks regarding the manuscript.

1. The addition of Perlin noise for the simulated data is a good call. However, I am not convinced it fully recapitulates the noise sources stemming from the imaging process. The addition of shot noise could induce another source of error commonly present in the data. The authors could discuss if the way the Perlin noise is applied is sufficient to simulate this source of error.

- Thank you for pointing this out – it had not occurred to us to consider shot noise. Visually, high octave Perlin noise appears similar to shot noise (compare Fig S1_4 in S1 Appendix to Fig 7 in this document). To investigate further, we perform a small study that we have enclosed in this “Response to Reviewers” document that we intend to publish alongside the manuscript. Specifically, we add shot noise to a single synthetic example, ST_1, and report the influence of shot noise on ground truth recovery in Fig 7. To accomplish this, we introduce shot noise as a Poisson distribution and define different values for λ as the ratio of the frame intensity to “lambda ratio”, a term we use to describe the factors that range between 1 and the maximum intensity value in the first frame of the movie (see Fig 7). In Fig 7, we show that percent peak mean absolute displacement (MAD) error and peak E_{cc} strain mean absolute error (MAE) values fall within the same range as the ones we obtained when Perlin noise was introduced. Overall, we really appreciate the suggestion and chance to ensure that our code is robust, and believe that this additional study along with our plan to publish the “Response to Reviewers” addresses the reviewer comment. In addition, we now mention “shot noise” as an alternative to Perlin noise in the revised manuscript highlighted in red.

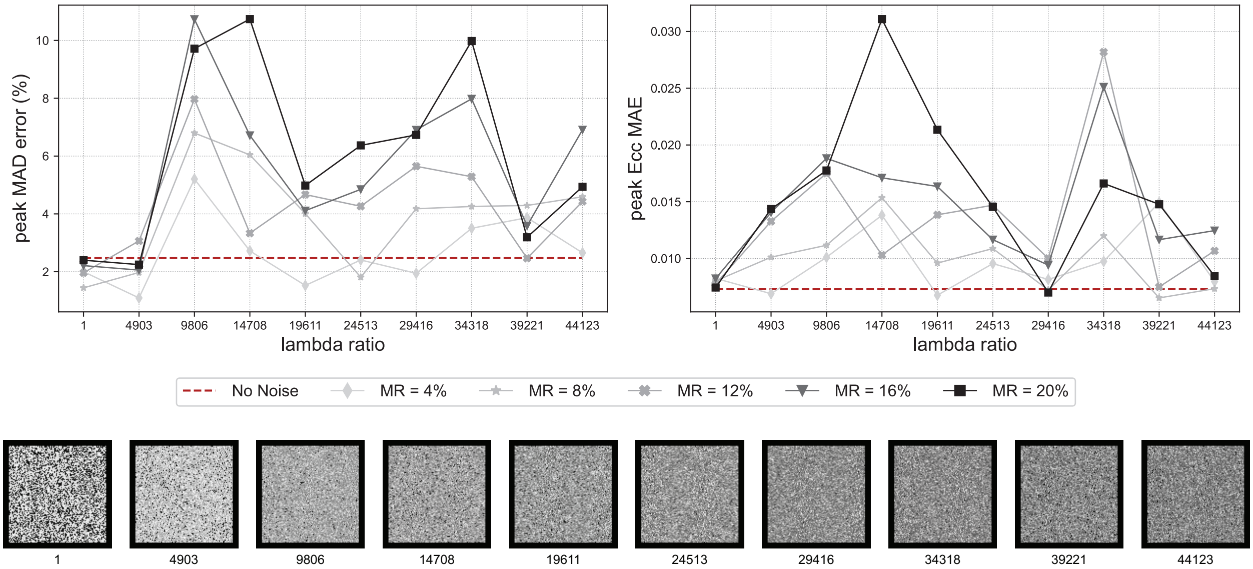

Figure 7: Results of validation against noisy synthetic data based on ST_1 for different shot noise lambda values and magnitude ratios (MR).

2. The authors estimate the microbundle thickness to be a 400 um in the FEM simulation, instead of varying as a function of distance from the micropillars. I would like the authors to discuss the impact of the design decision on the model validity, or to provide a more detailed justification for the choice.

- Thank you for pointing out that this is a major limitation of our approach to synthetic data generation for "Type 1" data. Keeping the thickness of the tissue constant was primarily motivated by our desire for simplicity – in other words, we wanted to ensure that we had enough detail to mimic the beating behavior of the cardiac microbundle, while keeping the approach relatively simple as to keep the focus on the key contribution of the manuscript: the data analysis software. To this end, we simply used a thickness of 400 μm as it is reported as the average experimentally measured thickness in the middle region in the relevant literature for "Type 1" data. Notably, we are particularly interested in computing the strains in this region, where the effects of the applied boundary conditions and changes in tissue thickness towards the edges become less significant. However, despite the relative simplicity of the "Type 1" synthetic data generation, our synthetic "Type 2" data (see S1 Appendix "Synthetic dataset" Section) generation is much more involved, and discussed in extensive detail in the manuscript "Jilberto J, et al. A data–driven computational modeling for engineered cardiac microtissues. Acta Biomaterialia. 2023 (in press)." To address the concern about the simplicity of the "Type 1" example not being challenging enough, in the revised version of our manuscript, we have added strain data comparisons for the more complex "Type 2" example data (see S1 Appendix Fig S1_26). Overall, we believe that the FEA models used (especially including the addition of the more complex "Type 2" strain validation) are sufficient for framework validation, though our simple "Type 1" model certainly does not fully capture the complexity of these systems. We now discuss the limited goal of our computational model (to inform the beating behavior of generated synthetic data), and the potential limitations of its simplicity in our revised manuscript in "Synthetic data generation" Section highlighted in red.

3. The impact of the current study may remain obscure to a reader if they're not well acquainted with the field. In particular, I feel the conclusion should more clearly reflect the advantages of cross-system compatibility how improvements in the field relate to tissue engineering studies.

- Thank you for pointing this out. We really appreciate this feedback, and the reminder to emphasize the benefits of cross-system compatibility for the broader cardiac tissue engineering field. We have updated the "Conclusion" Section of the revised manuscript to further clarify the impact of our work.

4. While the thoroughly reported supplementary material is informative, in my opinion it could be more organized for easier reading. In its current form, it is difficult to follow and appreciate the 15 pages of error calculations (pages 44 to 59). I am not convinced this way of reporting the findings is necessary, or if e.g. the MAE in subdomains could simply be stated numerically for more compact presentation.

- Thank you for pointing this out. We note that in addition to the detailed error representations that we included in the supplementary document (which we prefer to keep for the sake of completeness), we also provided 4 tables that briefly summarized the findings of the validation analysis. In the revised S1 Appendix, we now add an extra table (Table S1_3) to provide a more detailed summary of the error calculations in a more compact presentation as suggested by the reviewer.

Referee 3 Comments and Response

The manuscript titled "MicroBundleCompute: automated segmentation, tracking, and analysis of subdomain deformation in cardiac microbundles" presented an open-source computational tool able to analyze the beating of cardiac microbundles and provide quantification on their mechanical metrics. The authors performed an extensive validation on several experimental datasets and conclude that due to its ease of use and low computational demand this computational tool could be broadly used to analyze beating microbundles. The paper is well written, however, there are some major concerns to address:

1. Could the authors explain in more detail how the three-dimensional microbundle geometry is generated from the 2D coordinates? Authors refer to a reasonable tissue thickness of 400 um to extrude the 2D surface, but is that value is dependent on the different experimental dataset. Cross-sections of the tissues should be used instead to measure the tissue diameter and use that as input. Could the authors elaborate on how to adapt this parameter to each tissue type?

- Thank you for pointing this out. The model the reviewer is referring to is designed based on "Type 1" data only. We consider a constant tissue thickness of 400 μm since it is, on average, the experimentally reported thickness at the middle region of the microbundle for "Type 1" data [1]. It is important to note that our FEA model for "Type 1" data is quite minimal and is designed simply to mimic the inhomogeneous beating behavior of a cardiac microbundle. It is beyond the scope of this paper to create an entirely accurate computational model of these systems – rather, our main goal is to use the output of the model to generate synthetic examples of beating microbundles with known ground truth and that exhibit realistic behavior sufficient to validate our framework. For the "Type 2" data, we had the opportunity to include a more accurate and a more detailed FEA representation of the microbundles that is described at length in this recently published work [2]. We base the generation of synthetic data of "Type 2" on this more complex model, as we mention in the revised manuscript. We have updated the revised manuscript to clarify that our model is an oversimplified one and has a single purpose: inform the beating behavior of computationally derived *synthetic data for model validation.* Changes to the manuscript are present in "Synthetic data generation" Section and are highlighted in red.

1. Javor, Josh, et al. "A microtissue platform to simultaneously actuate and detect mechanical forces via non-contact magnetic approach." Journal of Microelectromechanical Systems 30.1 (2020): 96-104. `10.1109/JMEMS.2020.3036978`
2. Jilberto Javiera, et al. "A data–driven computational modeling for engineered cardiac microtissues." Acta Biomaterialia. 2023(in press)

2. In page 13, authors model the cardiac tissue as a nearly-incompressible transversely isotropic hyperelastic material. How does this take into account different cell composition of the microbundles and the different ECM, which may change over time and depending on the cellular composition? Have the authors considered these implications?

- Thank you for pointing this out. As stated in our response to Comment 1, the FEA model for "Type 1" data is an oversimplified representation developed for the main purpose of creating synthetic data for model validation. However, for "Type 2" data, we have the opportunity to include a recently published and more sophisticated computational model (with a more accurate representation of cell composition and ECM components), whose implementation lies outside the scope of our manuscript but is described in detail in [1]. In the revised version of the manuscript, we also added strain validation with these more complicated synthetic data to ensure that our pipeline would still function in the presence of more complex tissue behavior. These results are shown in S1 Appendix Fig S1_26. Hence, we have

performed our validation studies against both a simplistic model and a more complicated one and found that the "MicroBundleCompute" framework is able to handle both. In conjunction with our response to Comment 1, we have updated the revised manuscript to clarify that our FEA model is an oversimplified one and has a single purpose: inform the beating behavior of computationally derived *synthetic data for model validation.*

1. Jilberto Javiera, et al. "A data–driven computational modeling for engineered cardiac microtissues." Acta Biomaterialia. 2023(in press)

3. In page 18, "By design, our computational framework requires a minimum of 3 beats to be present...". How do the authors plan to account for experimental conditions where tissues decrease considerably their frequency of contraction? How long do the videos need to be?

- Thank you for pointing out that this was a potentially limiting requirement. To clarify, we impose this requirement so that we have enough information to adequately identify relaxed frames and to be able to meaningfully compute beat period and beat amplitude. For reference, to find valleys (the most relaxed tissue positions), we perform preliminary tracking to find peaks (most contracted positions) first and then we define valleys as the midpoints between 2 consecutive peaks. In future iterations of the software, we plan to update this approach and identify valleys directly. Of note, our real example dataset that we have analyzed with "MicroBundleCompute" contains microbundles beating at frequencies ranging between ~ 0.2 Hz and ~ 1.65 Hz. This means that it would take a maximum of 15 seconds to capture 3 beats. We have updated the revised manuscript to clarify the motivation behind this restriction in our software implementation, and our intention to improve it in future iterations. Changes to the manuscript are present in "Temporal segmentation" Section and are highlighted in red.

4. In page 20, point 2. How do the authors guarantee that the tissues are in the relaxed position?

- Thank you to the reviewer for raising this crucial point. With our pipeline, we test whether or not the video starts from a relaxed position (valley frame) and then warn the user if the input video does not start from a detected valley frame. To implement our test for starting from a valley frame, we assume that the tissue should be undergoing contractile deformation in the standard case, but do not want to prevent the analysis of exceptional cases (e.g., we want to also accommodate cases where the tissue is actuated and stretched purposefully such as example 1 from "Type 3" data). Therefore, we do not perform the valley frame adjustment automatically but rather require the user to input the frame number of the first valley if the warning appears. Based on the user input, the original movie is automatically adjusted by removing all frames that precede the set first valley frame number. We plan to improve this aspect of the software in the future for enhanced automation as the need arises. We have updated the revised manuscript to reflect our future plans. Changes are highlighted in red in the "Conclusion" Section.

5. In page 24, "From a methodological perspective,...". Does that mean that the user requires a specific training? The fact that a user has to observe the samples and choose the subdomain size per video suggests that each video has to be analyzed manually and not automatically, which hinders the high-throughput claim of the software. Can the authors elaborate on this?

- Thank you for bringing this concern up. We meant here that the user should be mindful of such choices and perhaps perform manual examination of subdomain size once per dataset. For example, the user could select a single movie from a batch of 100 to confirm that the subdomain division is appropriate based on the observed displacement field. We have provided additional clarifications in the revised manuscript concerning this point. We apologize that this was not initially clearer. Changes are highlighted in red in "Synthetic data examples" Section.

6. In page 25, "time series strain plots highlight the synchrony (or lack of synchrony)...". Can the authors elaborate on this point? Where the tissues electrically paced? What is the lowest and the highest frequency that the software can track?

- Thank you for letting us know that this statement is not clear. None of the tissue examples in our dataset are electrically paced although as we note in the manuscript and in S2 Appendix, example 1 from "Type 3" data is actuated by applying sawtooth pressure waves with ~ -6 kPa peak amplitude using a microfluidic pump. Particularly, in the quoted phrase from our manuscript, we were comparing the spatial synchrony/lack of synchrony in contraction among the different subdomain regions (i.e. are all regions contracting at the same time or is there a lag?). For reference, our real example dataset that we have analyzed with "MicroBundleCompute" contains microbundles beating at frequencies ranging between ~ 0.2 Hz and ~ 1.65 Hz. We have not performed an extensive study to check the limitation of the software in terms of highest/lowest trackable frequency, but we have not encountered an example where the pipeline breaks due to beating frequency. We have clarified this in the revised manuscript, highlighted in red in "Experimental data examples" Section.

7. Force measurements from pillar tracking are not well described per type of experimental set up. How do the authors plan on implementing this functionality according to each type of microbundle that the users might have (including different geometry of the pillars and young modulus)?

- Thank you for pointing this out. Since pillar tracking is not the main focus of the current manuscript, we include more details about this functionality in S3 Appendix where the process is described in detail. We accommodate for different pillar configurations following 2 approaches: 1) the user can specify a circular or a rectangular pillar cross section, input the relevant geometry parameters as well as the Young's modulus of the material, and then the software computes the pillar stiffness by approximating the pillars as beams, or alternatively, 2) the user can directly input an experimentally measured stiffness value for the pillars. We note that the latter approach is more common in practice among experimentalists. Currently, pillar tracking is possible with "Type 1" and "Type 2" data – the 2 pillar platforms. "Type 3" data incorporates more complex pillar configurations that we plan to accommodate in future iterations of the pillar tracking functionality, which is still a work in progress. We note the reference to S3 Appendix in the main manuscript, now highlighted in red.

8. In figure 6a-II there are some purple dots outside of the tissue region. How does the author exclude these data or avoid to take into account artifacts in the strain calculations?

- Thank you for pointing this out. We calculate the strains only within the region marked by the dashed white box where marker points within this region only are considered for strain calculations. As mentioned in the manuscript in "Subdomain spatial segmentation" section, we define a rectangular subdomain, specified based on the tissue mask, for more reliable strain calculations which are carried out within smaller subdivisions of the defined subdomain. All data points outside this subdomain are excluded from these calculations. We now note this briefly in the manuscript in the caption of Fig 6, highlighted in red.

Minor concerns:

1. In abstract: "requires little to no parameter tuning" is a misleading sentence that should be adjusted.

- Thank your for pointing out that this statement is not clear. We have rephrased this sentence in the revised manuscript and made it more specific.

2. In abstract: "runs quick on a personal computer". Compared to what? Should be rephrased to "low computational demand"

- Thank you for pointing this out. We have rephrased this part of the sentence in the revised manuscript.

3. It is not clear if the software has an UI to benefit the scientific community, can the authors elaborate on that? Besides, is the software compatible only with windows or can it work on macOS and linux?

- Thank you for pointing this out. The software does not come with a graphical user interface, but we provide an elaborate tutorial regarding installing the software and running it on GitHub (`https://github.com/HibaKob/MicroBundleCompute`). We also provide a python script, `run_code.py`, which serves as a practical user interface for running the software. From a practical perspective, we prefer to avoid having a graphical interface to accompany the software because 1) it might give rise to unnecessary errors especially regarding version compatibility, and 2) it might require additional steps from the user which hinders the automated analysis process. Additionally, as indicated on the GitHub page, we have successfully tested our software on 3 operating systems, ubuntu, macOS, and Windows, using GitHub Actions.

4. In page 12 "Overall, these approaches are suitable when only basic information...". Previous sentence mentioned software also provide absolute values of force. This sentence may be misleading to the scientific community.

- Thank you for your remark. We are not sure that we understand this comment exactly – for context, we consider outputs that comprise single quantity of interest *mean values* (e.g., mean displacement, total force, average velocity) as "basic information" because they lump the information that one can get from full-field data into a single value per frame. In the revised manuscript, we have re-phrased "basic information" as "averaged values" for clarity.

5. In page 14 and 15, authors refer to recording of time-lapse videos of tissue contractions at 30Hz or 65Hz. Those are very low frame rates to record the tissues. Can the authors explain these choices?

- Thank you for pointing this out. We believe that for our application, tracking tissue contractility and extracting mechanical metrics, a rate of 30 Hz or above should suffice, especially given that these cardiac tissues naturally beat at ~ 0.4 Hz. Please refer to the following previous work where capturing time-lapse videos was performed at comparable frame rates [1,2,3]. We note that had we been extracting time-related data, for example time-to-peak or relaxation time, rather than spatial information, or had we been interested in capturing conduction velocity, calcium transients or action potentials, higher frame rates would have been recommended for more accurate quantification [4].

  1. Boudou, Thomas, et al. "A microfabricated platform to measure and manipulate the mechanics of engineered cardiac microtissues." Tissue Engineering Part A 18.9-10 (2012): 910-919. `10.1089/ten.tea.2011.0341`
  2. Hansen, Katrina J., et al. "Optical method to quantify mechanical contraction and calcium transients of human pluripotent stem cell-derived cardiomyocytes." Tissue Engineering Part C: Methods 23.8 (2017): 445-454. `http://doi.org/10.1089/ten.tec.2017.0190`
  3. Tamargo, Manuel Alejandro, et al. "milliPillar: a platform for the generation and real-time assessment of human engineered cardiac tissues." ACS biomaterials science & engineering 7.11 (2021): 5215-5229. `https://doi.org/10.1021/acsbiomaterials.1c01006`
  4. Sala, Luca, et al. "MUSCLEMOTION: a versatile open software tool to quantify cardiomyocyte and cardiac muscle contraction in vitro and in vivo." Circulation research 122.3 (2018): e5-e16. `https://doi.org/10.1161/CIRCRESAHA.117.312067`

6. In tracking region mask generation. It is not clear if the user will have to do it manually or if it is automatically generated by the software. In the case that is generated by the software, which is the criteria to select between the 3 segmentation methods? Which one is the most accurate method to create the mask?

- Thank you for pointing this out. Both automatic and manual mask generation options are available. In fact, we allow the user to input a manually or externally generated mask in case the 3 mask segmentation functions that we provide within the software fail. There is no optimum or most accurate method among these 3 options as each one might be suitable for different imaging conditions. Furthermore, for each real example, we indicate whether the mask was generated automatically or manually in S2 Appendix. We have updated the revised manuscript to clarify this aspect more, and additional detail is provided in the implementation tutorial on GitHub. Changes are in "Tracking region mask generation" Section highlighted in red.

7. In page 24, "output reliable mechanical metrics in real experimental settings on condition. . . .". Is the software able to give as an output the noise level of the input sample and show the user the internal modifications done by the software?

- Thank you for this valuable remark. Currently, the software does not have this functionality of outputting the noise level. In future iterations of the software, we would like to address automated quality control and data pre-processing. We have updated the revised manuscript to reflect our future plans. Changes made to the "Conclusion" Section are highlighted in red.

8. In page 24, "while the remaining single example is a phase contrast video". Why did the authors choose phase contrast?

- Thank you for pointing this out. We include a phase contrast video because it was readily available to us within the dataset typically generated by co-authors in their experimental workflow, and because it provided us with an opportunity to showcase the additional versatility of the software by demonstrating that it can be used with data obtained from imaging modalities other than brightfield microscopy.
